# Supplementary material for: The Nedd4L ubiquitin ligase is activated by FCHO2-generated membrane curvature
Source: EMBO J. 2024 Oct 14;43(23):8. doi: 10.1038/s44318-024-00268-1 (PMC11612235; doi:10.1038/s44318-024-00268-1)
Supplement: Supplementary file 1 — Appendix [file 44318_2024_268_MOESM1_ESM.pdf]

Appendix

The Nedd4L ubiquitin ligase is activated by FCHO2-generated membrane curvature

Yasuhisa Sakamoto, Akiyoshi Uezu, Koji Kikuchi, Jangmi Kang, Eiko Fujii, Toshiro Moroishi, Shiro Suetsugu, and Hiroyuki Nakanishi

Table of Contents

Appendix Figure S1. Original pictures of Fig. 1D…..………………………………....2

Appendix Figure S2. Appendix Figure S1. Failure to detect the accumulation of Nedd4L…………………………………………………………………………….…..3

Appendix Figure S3. Inability of Nedd4L to ubiquitinate PY-motif mutated mSA-αENaC………………………………………………………………………………4

Appendix Figure S1

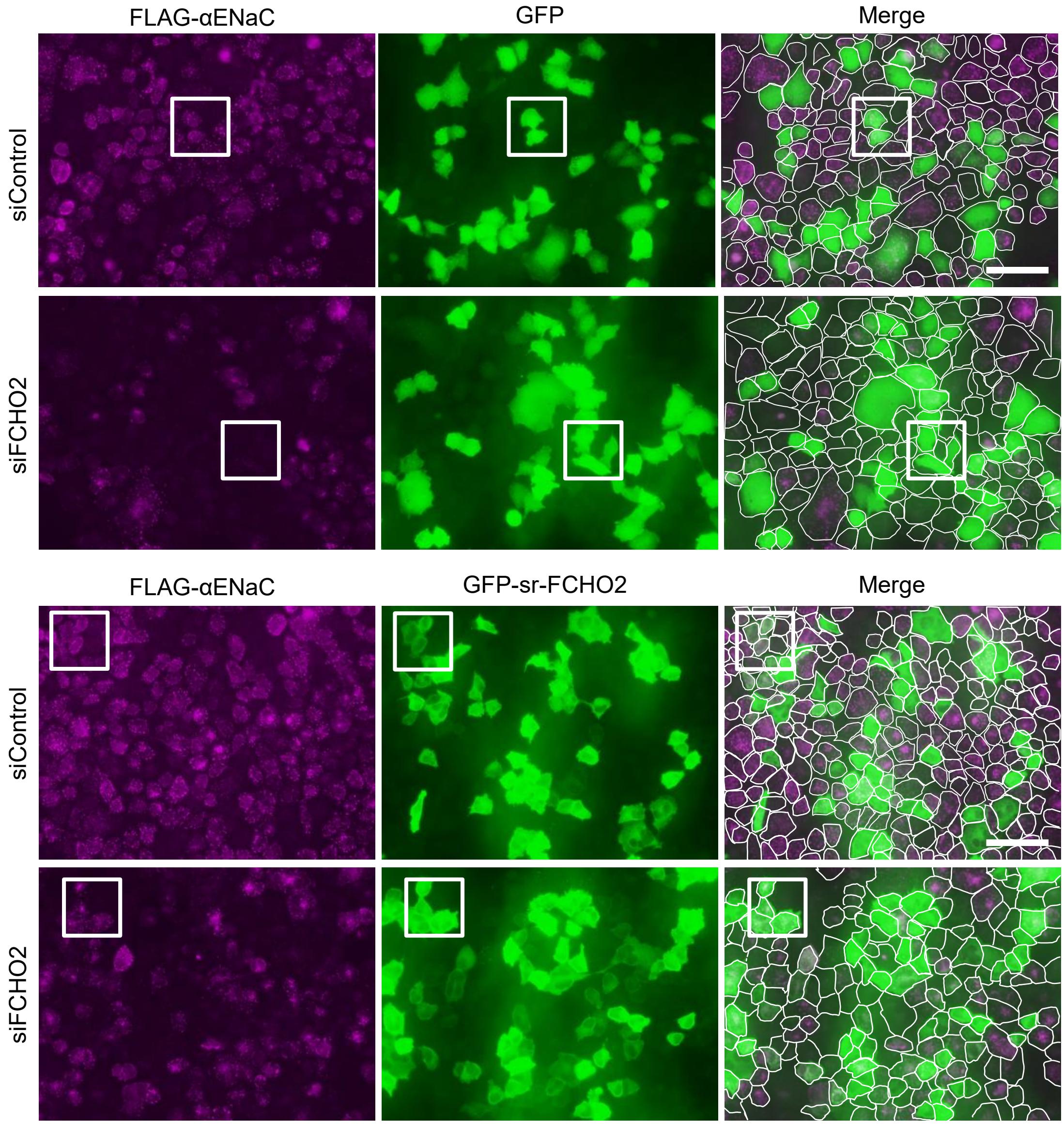

Appendix Figure S1. Original pictures of Fig. 1D. Solid boxed correspond to the images of Fig. 1D. The border of each cell is delineated by a solid line. Scale bars, 100 μm.

Appendix Figure S2

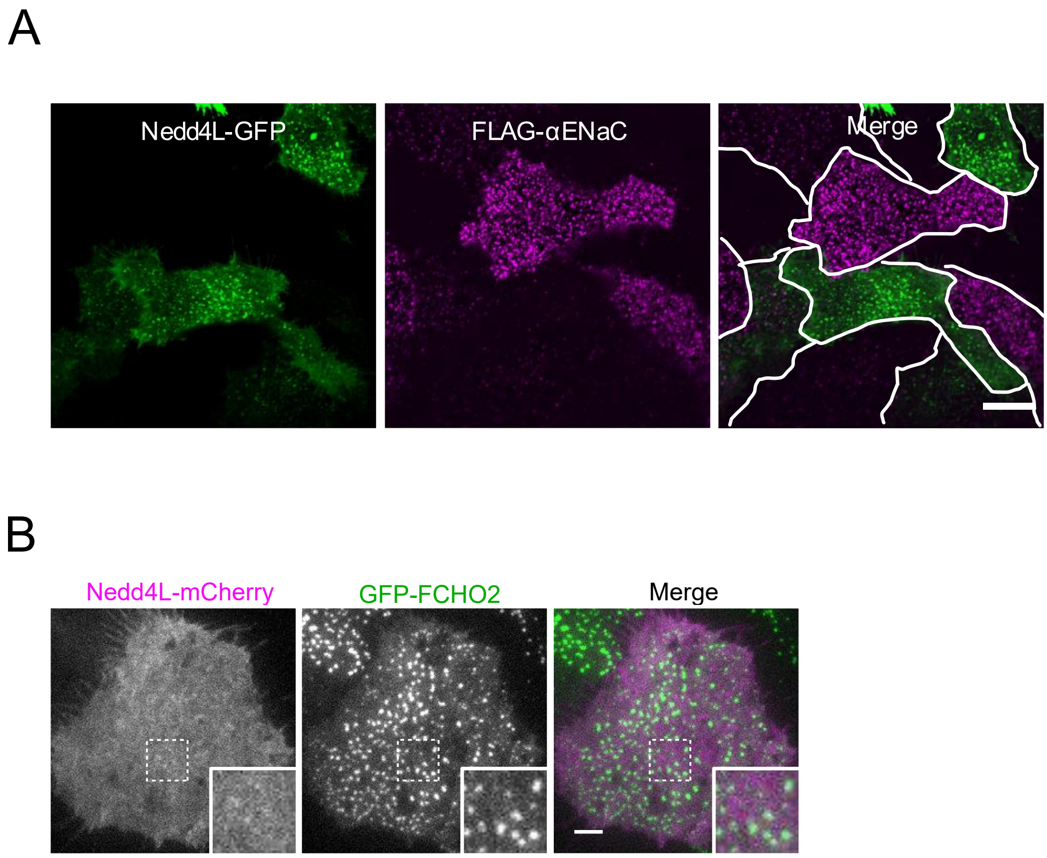

Appendix Figure S2. Inability to detect the accumulation of Nedd4L

A, Inability to detect the co-localization of Nedd4L with cell-surface αENaC. Nedd4L-GFP was expressed in αβγENaC-HeLa cells. Cell-surface αENaC was labeled with anti-FLAG antibody at 4°C, followed by immunofluorescence microscopy. The border of each cell is delineated by a solid line. Scale bar, 10 µm

B, Inability to detect the accumulation of Nedd4L-mCherry at GFP-FCHO2-positive spots. Nedd4L-mCherry and GFP-FCHO2 were expressed in wild-type HeLa cells, followed by TIRF microscopy. Scale bar, 2 µm.

Appendix Figure S3

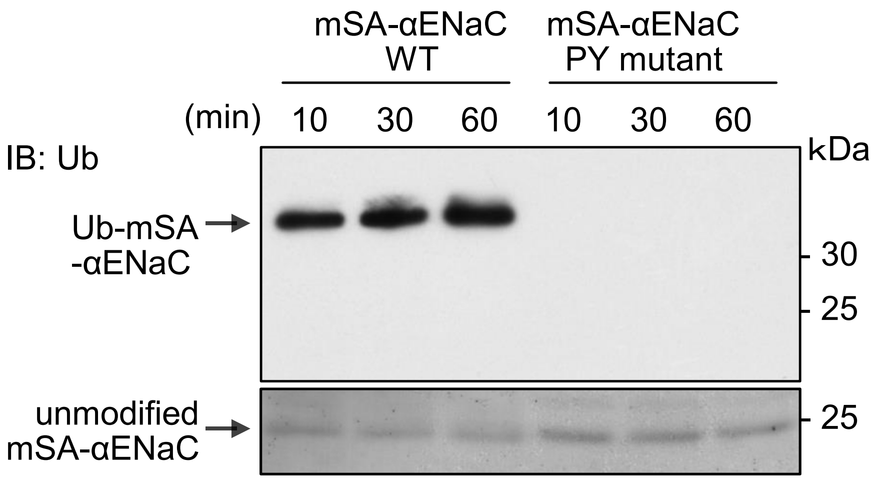

Appendix Figure S3. Inability of Nedd4L to ubiquitinate PY-motif mutated mSA-αENaC. An *in vitro* ubiquitination assay was performed at 0.7 μM Ca^{2+} using brain-lipid liposomes (~50% PS) associated with either wild-type or PY-motif mutated (Y644A in αENaC) mSA-αENaC. Incubation was carried out for the indicated periods of time. Samples were analyzed with immunoblotting (IB) (upper panel) and Ponseau-S staining (lower panel).
