# Supplementary figures and images for: The Nedd4L ubiquitin ligase is activated by FCHO2-generated membrane curvature
Source: EMBO J. 2024 Oct 14;43(23):8. doi: 10.1038/s44318-024-00268-1 (PMC11612235; doi:10.1038/s44318-024-00268-1)

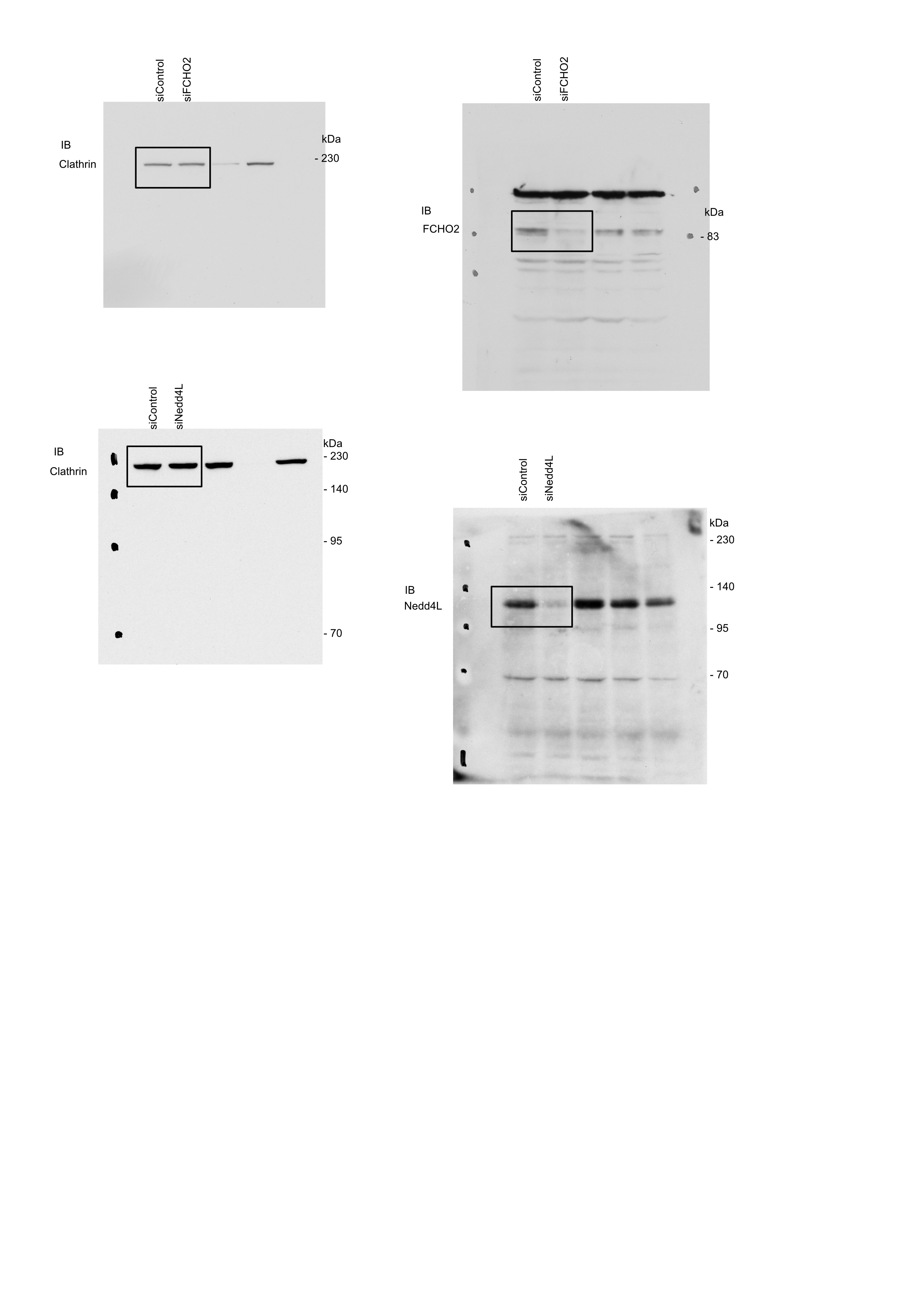

Supplement: Supplementary file 4 — Source data Fig. 1 [file 44318_2024_268_MOESM4_ESM.zip › EMBOJ-2023-114687_SourceDataForFigure1/1A/Fig1A_western.tiff]

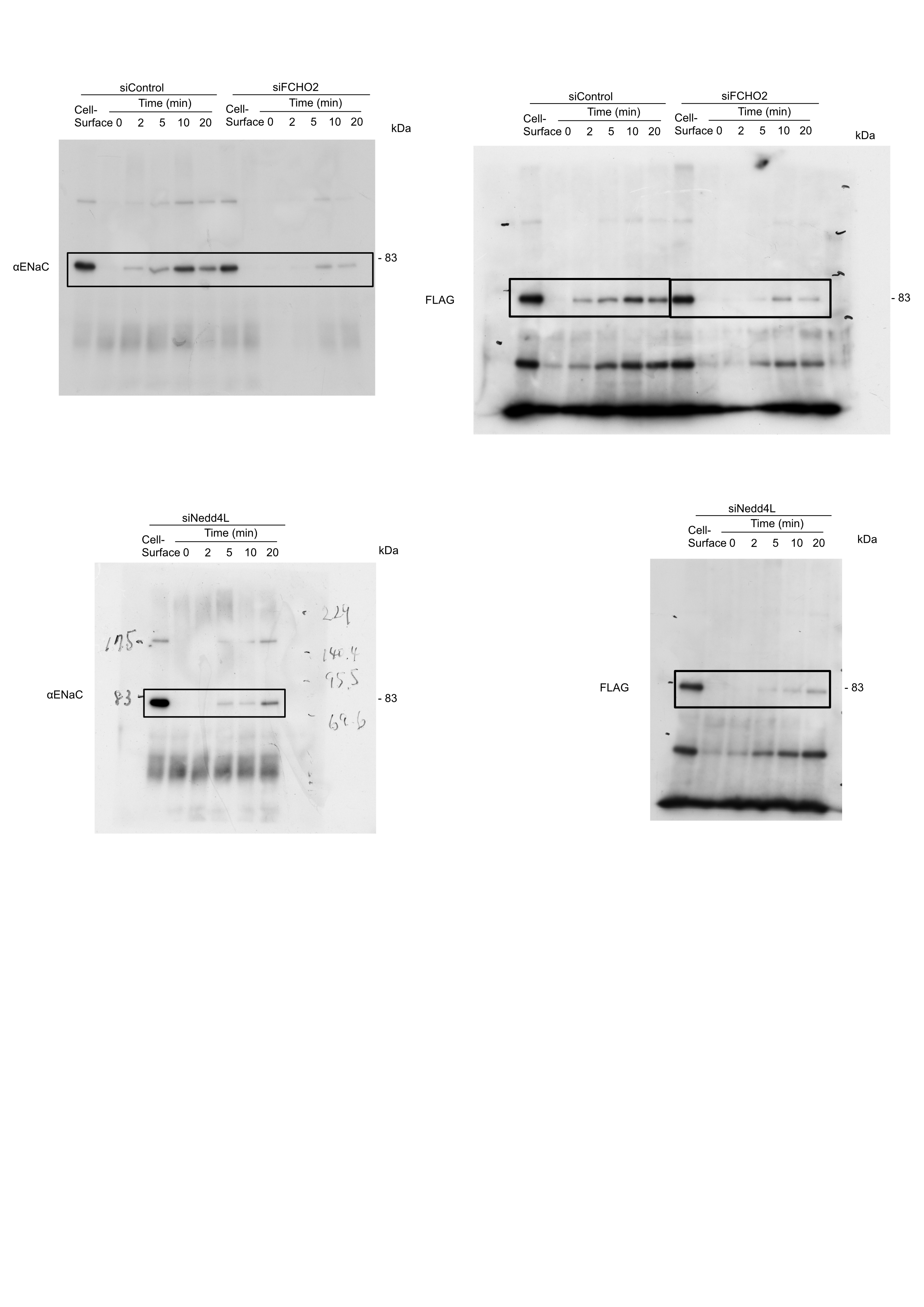

Supplement: Supplementary file 4 — Source data Fig. 1 [file 44318_2024_268_MOESM4_ESM.zip › EMBOJ-2023-114687_SourceDataForFigure1/1B/Fig1B_1.tiff]

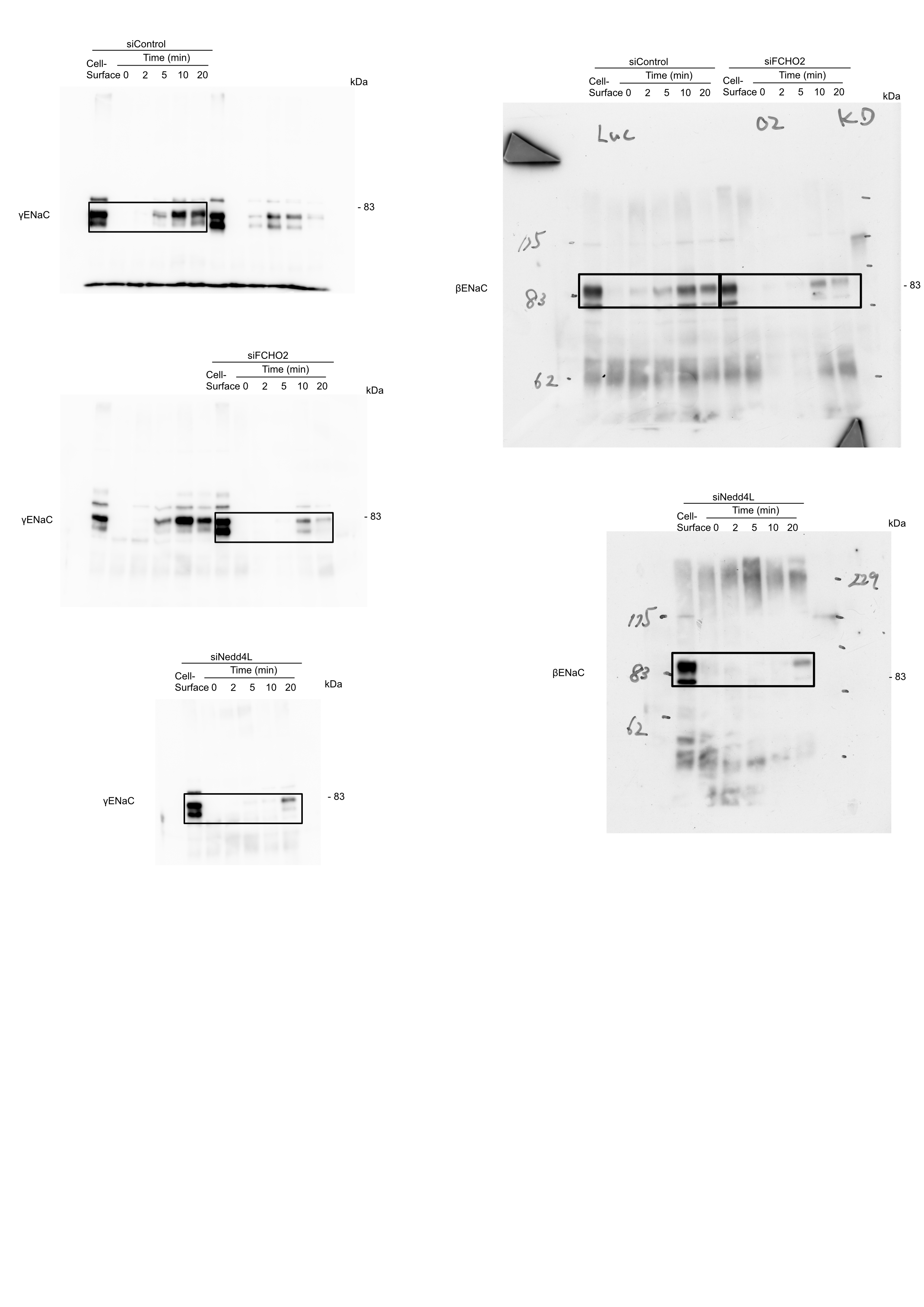

Supplement: Supplementary file 4 — Source data Fig. 1 [file 44318_2024_268_MOESM4_ESM.zip › EMBOJ-2023-114687_SourceDataForFigure1/1B/Fig1B_2.tiff]

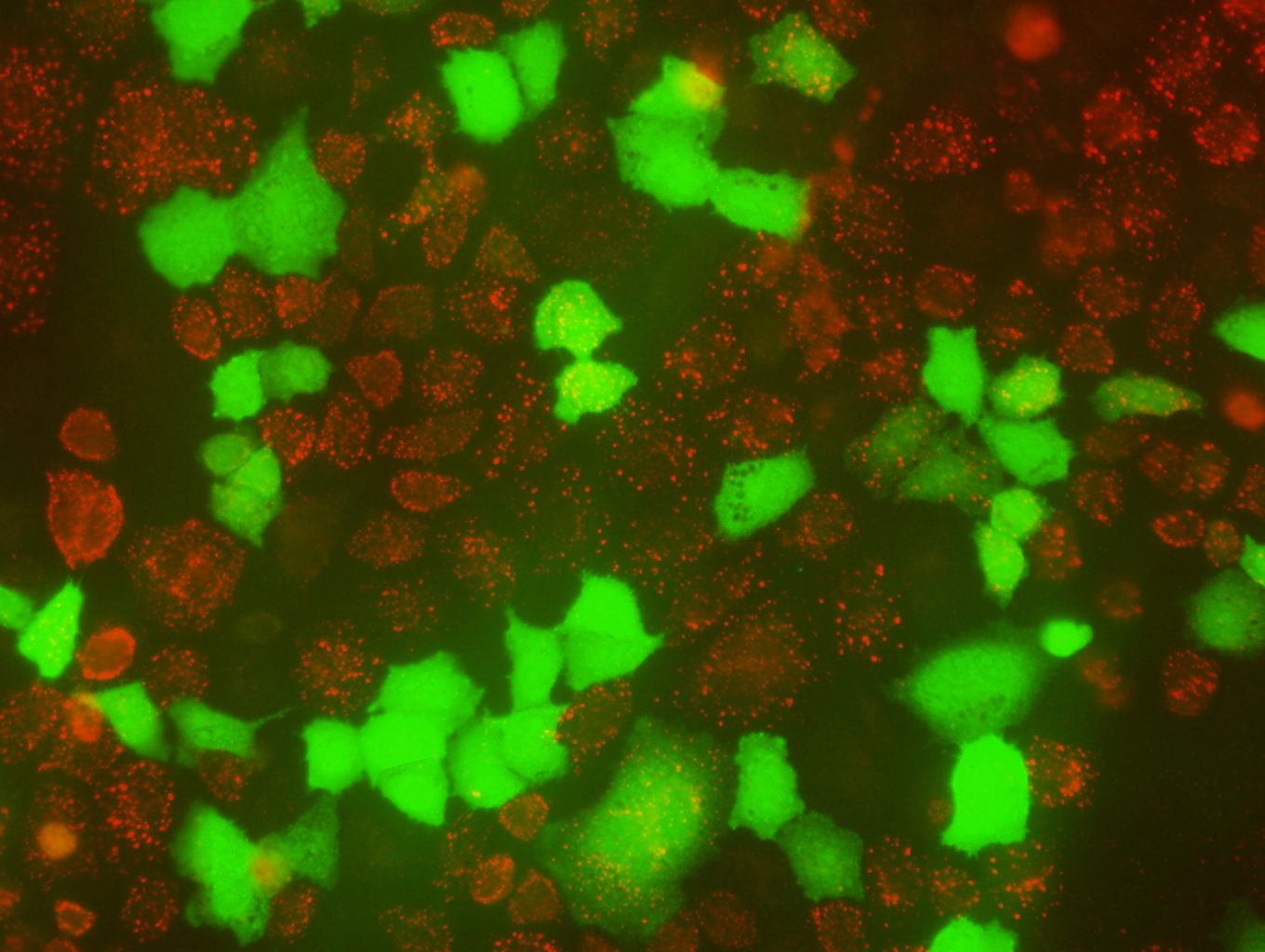

Supplement: Supplementary file 4 — Source data Fig. 1 [file 44318_2024_268_MOESM4_ESM.zip › EMBOJ-2023-114687_SourceDataForFigure1/1D/GFP x siControl.tif]

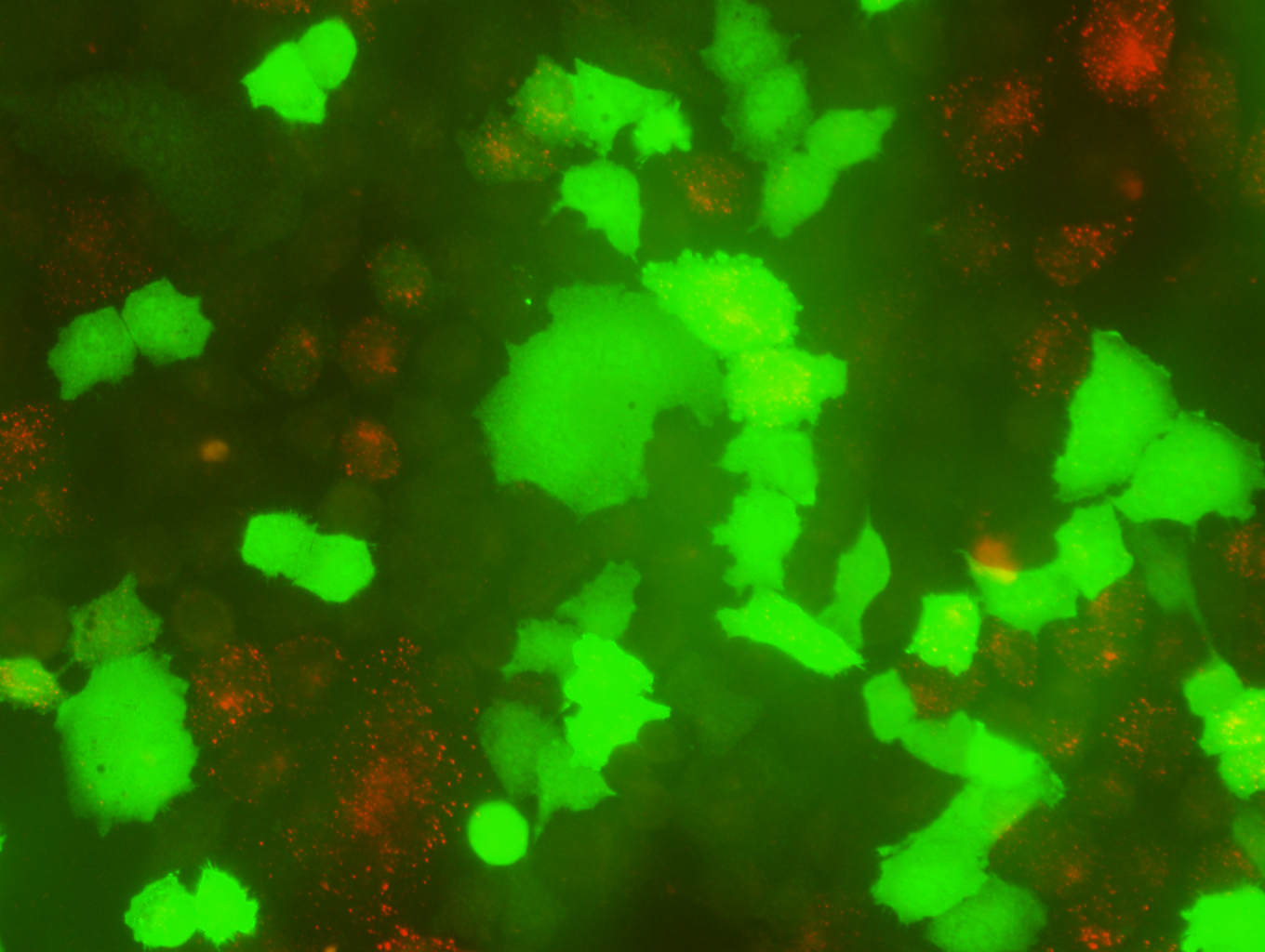

Supplement: Supplementary file 4 — Source data Fig. 1 [file 44318_2024_268_MOESM4_ESM.zip › EMBOJ-2023-114687_SourceDataForFigure1/1D/GFP x siFCHO2.tif]

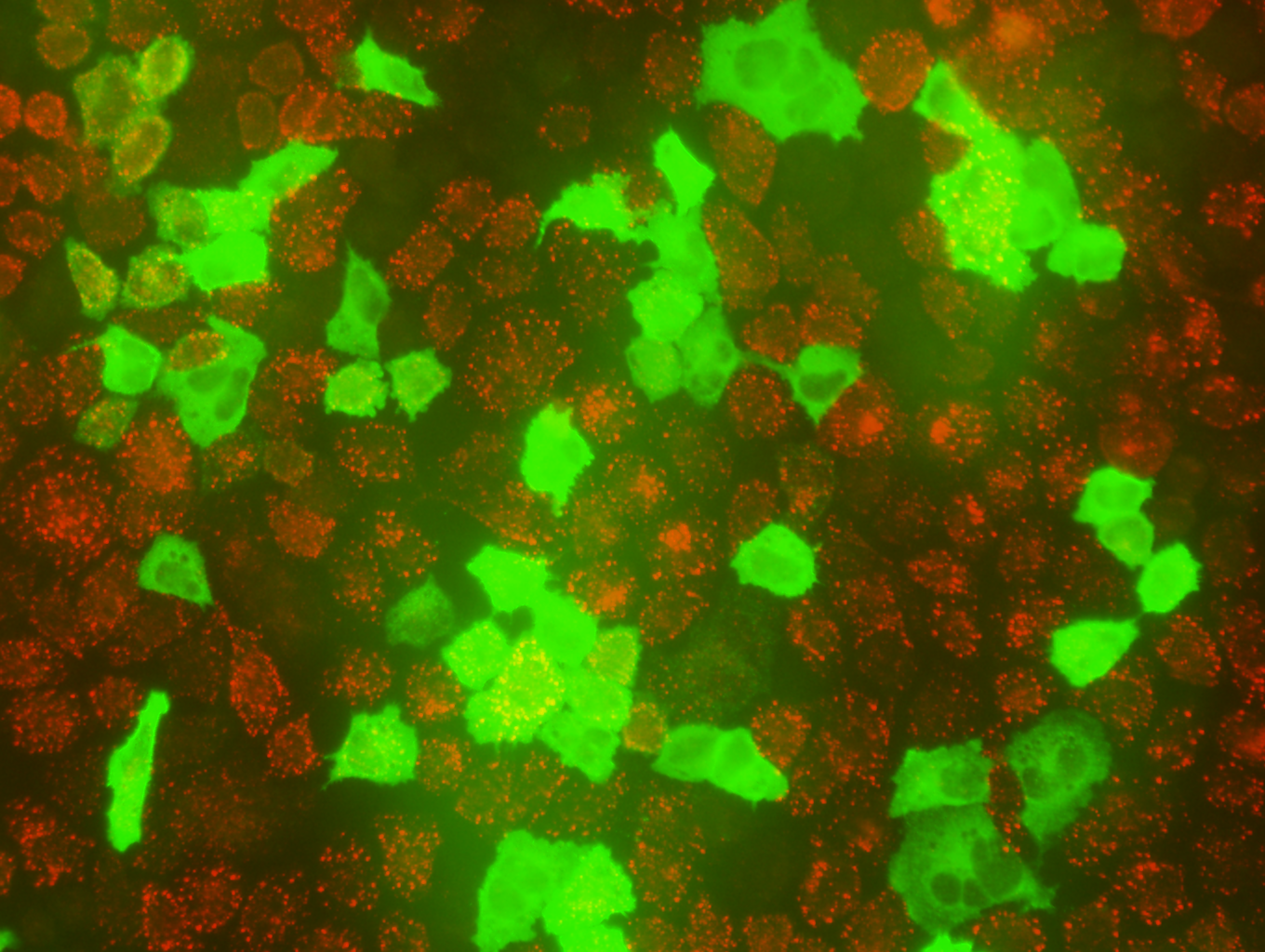

Supplement: Supplementary file 4 — Source data Fig. 1 [file 44318_2024_268_MOESM4_ESM.zip › EMBOJ-2023-114687_SourceDataForFigure1/1D/GFPsrFCHO2 x siControl.tif]

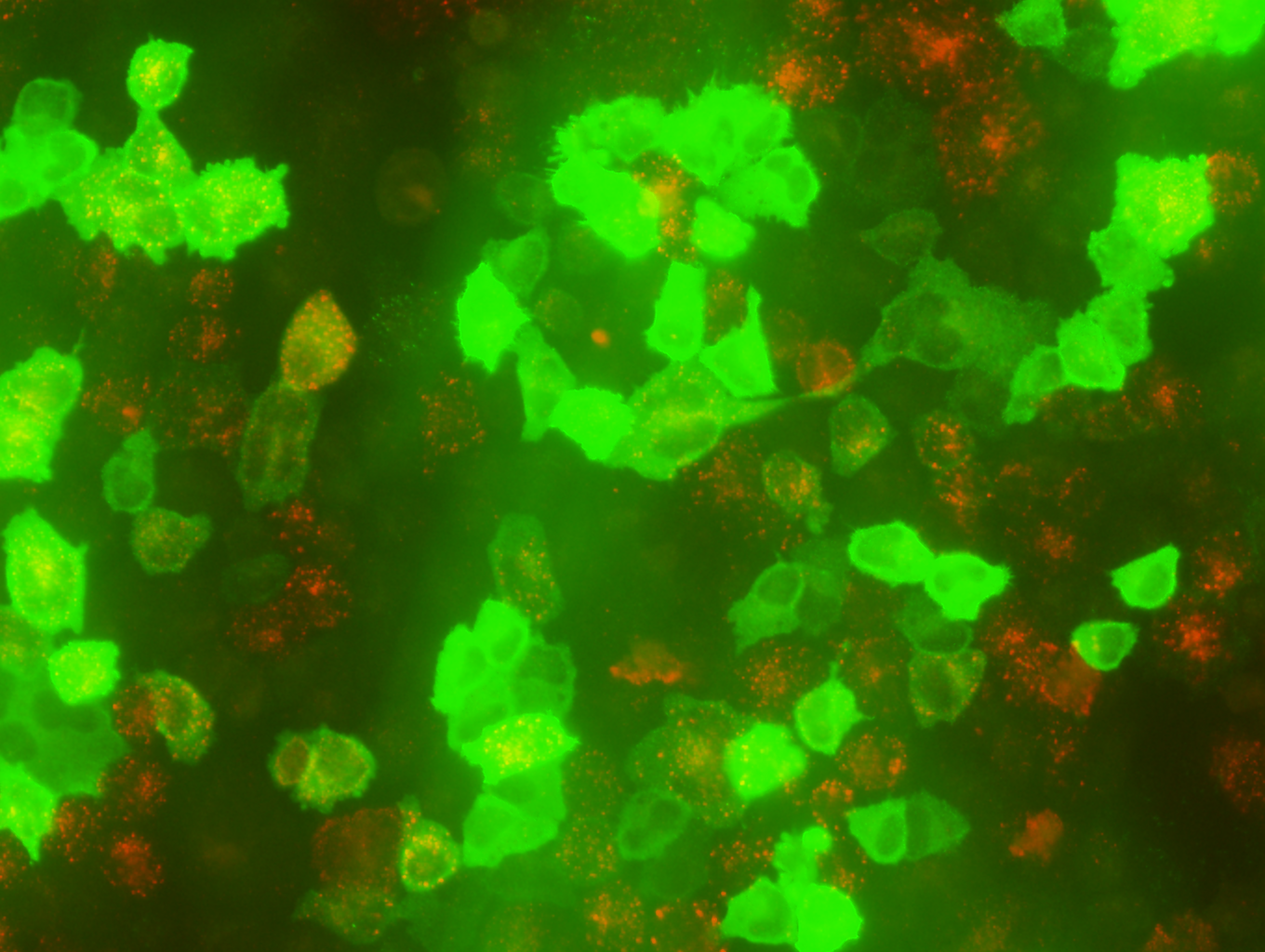

Supplement: Supplementary file 4 — Source data Fig. 1 [file 44318_2024_268_MOESM4_ESM.zip › EMBOJ-2023-114687_SourceDataForFigure1/1D/GFPsrFCHO2 x siFCHO2.tif]

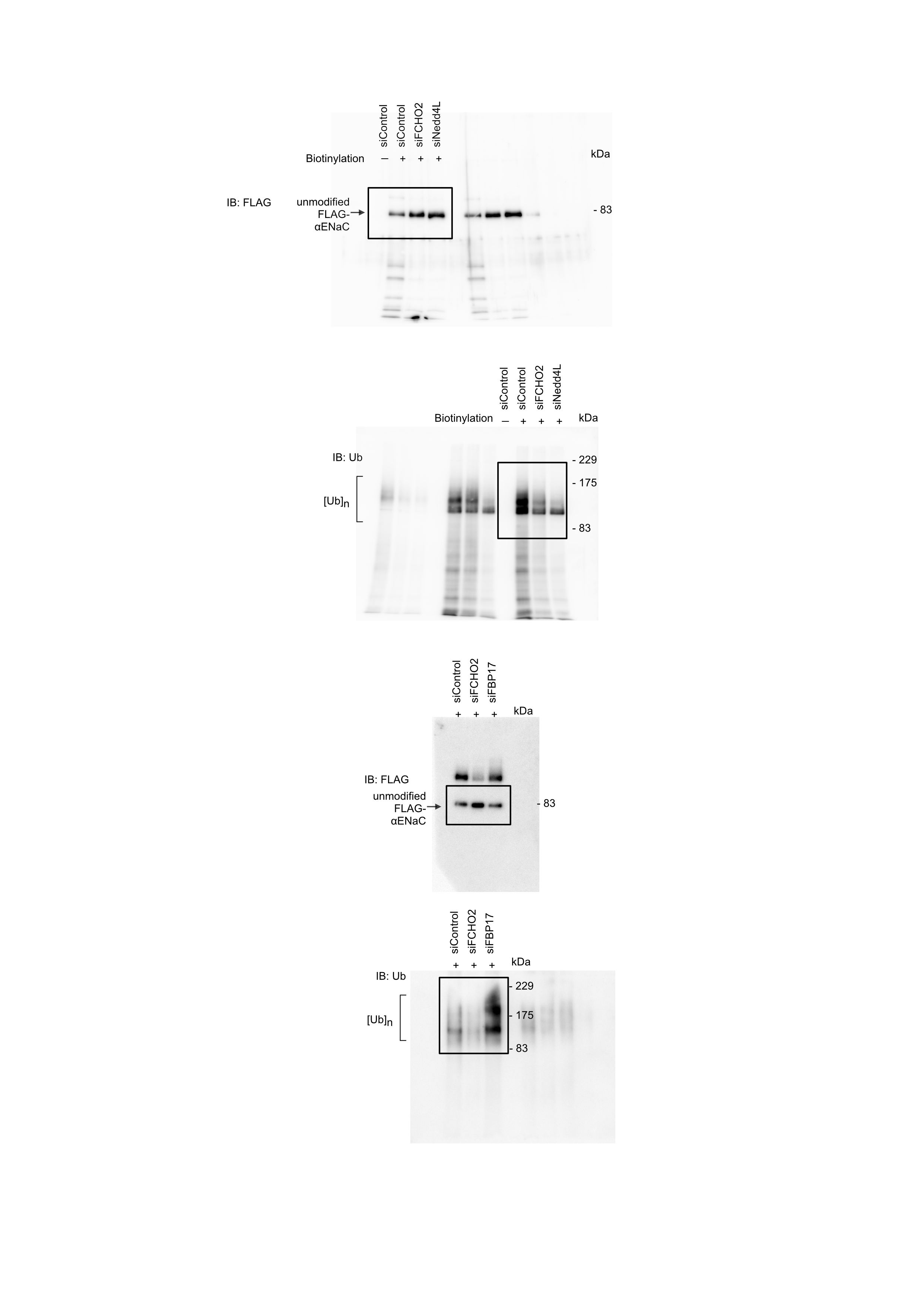

Supplement: Supplementary file 4 — Source data Fig. 1 [file 44318_2024_268_MOESM4_ESM.zip › EMBOJ-2023-114687_SourceDataForFigure1/1E/Fig1E.tiff]

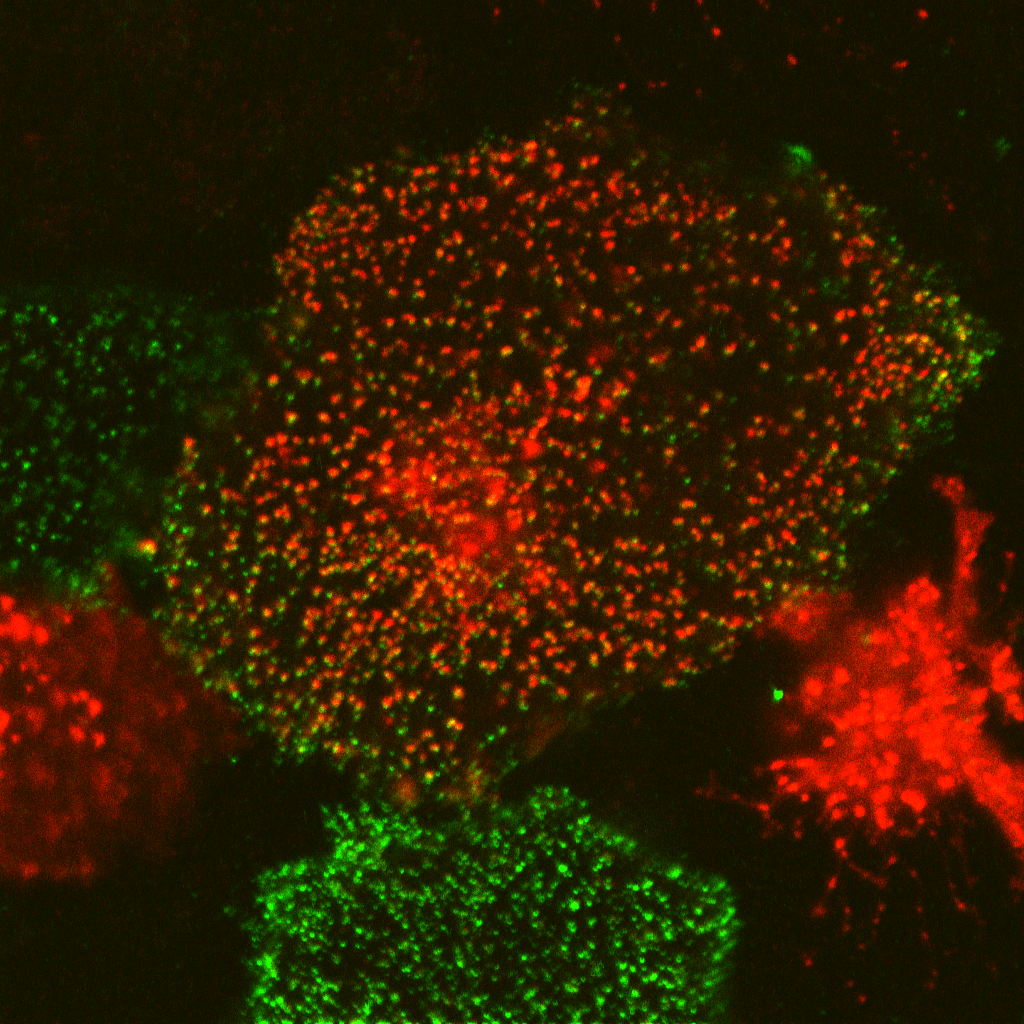

Supplement: Supplementary file 5 — Source data Fig. 2 [file 44318_2024_268_MOESM5_ESM.zip › Figure 2/2A/ENaC x Clathrin.tif]

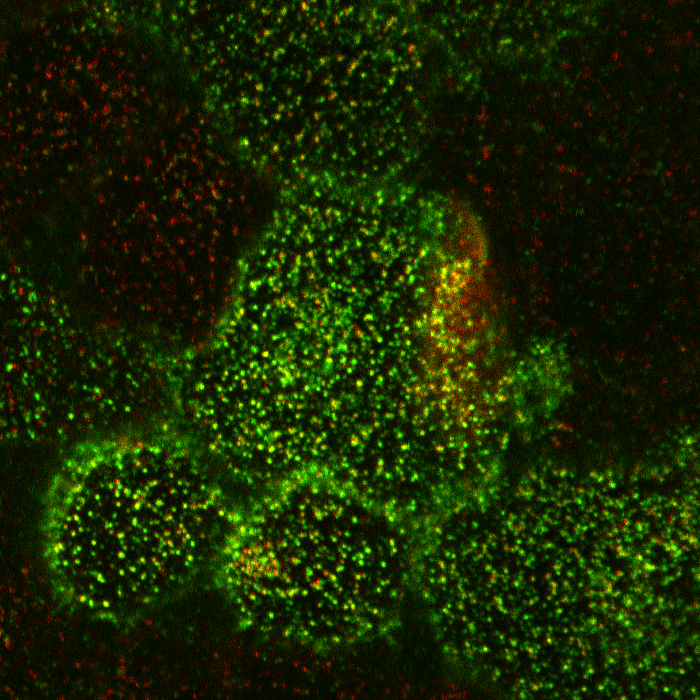

Supplement: Supplementary file 5 — Source data Fig. 2 [file 44318_2024_268_MOESM5_ESM.zip › Figure 2/2A/ENaC x FCHO2.tif]

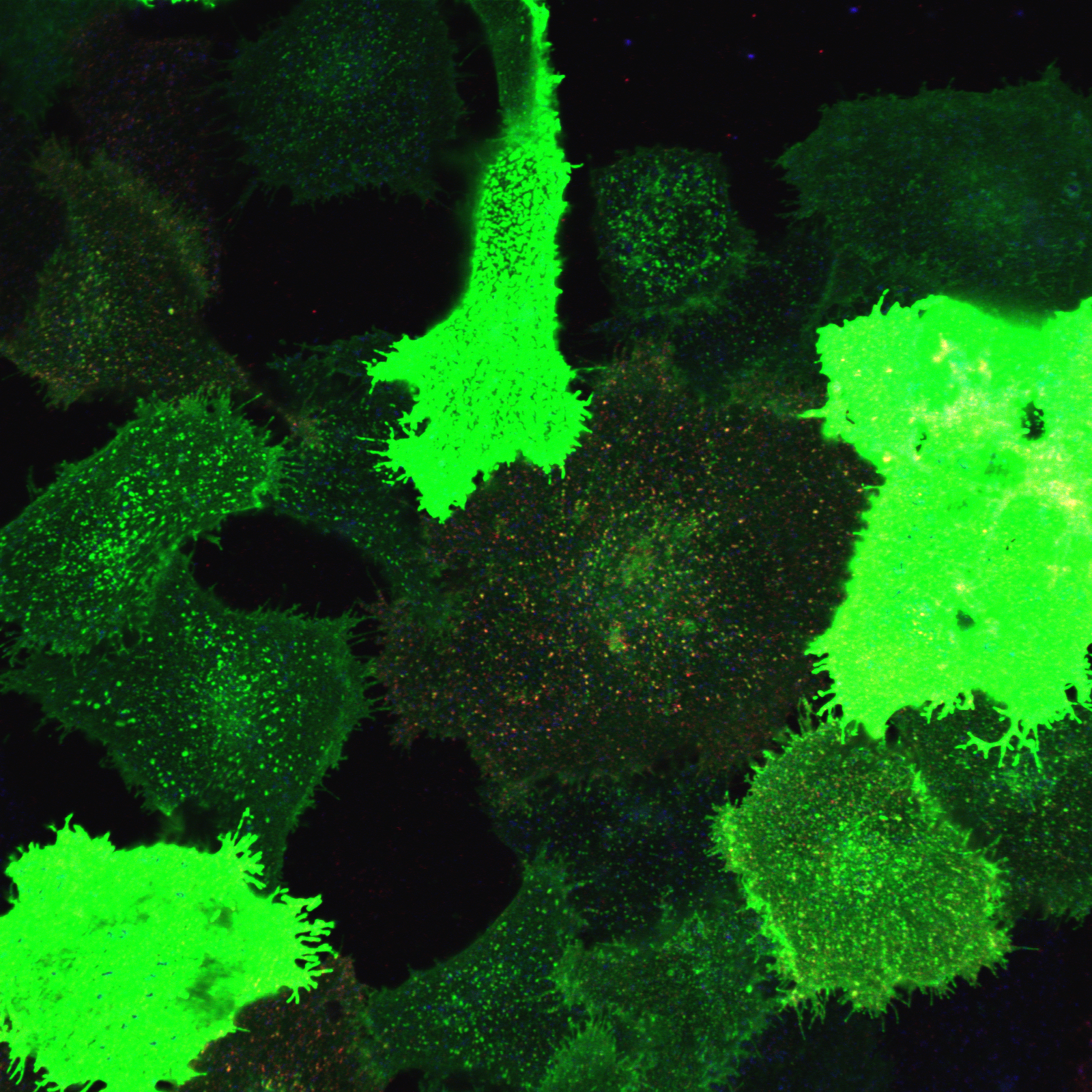

Supplement: Supplementary file 5 — Source data Fig. 2 [file 44318_2024_268_MOESM5_ESM.zip › Figure 2/2B/Nedd4LC922A x alphaENaC x FCHO2.tif]

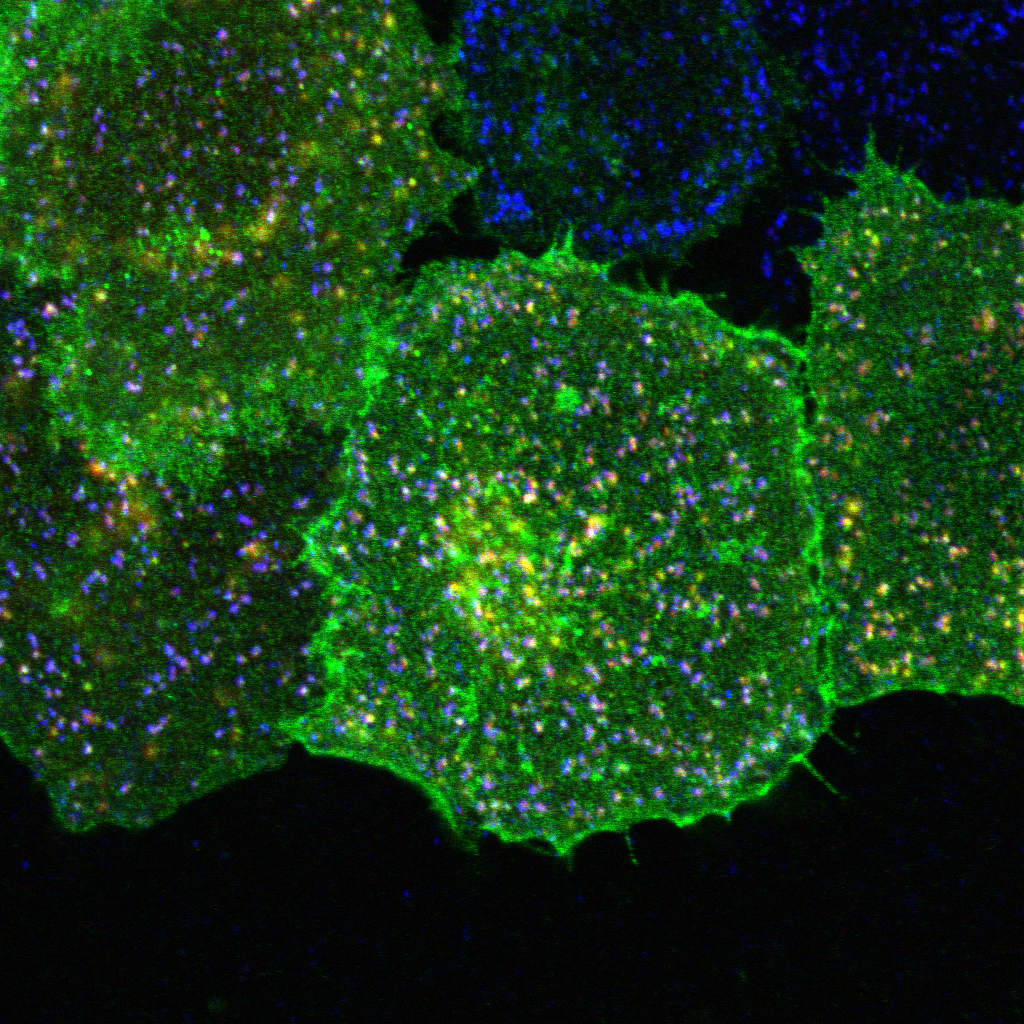

Supplement: Supplementary file 5 — Source data Fig. 2 [file 44318_2024_268_MOESM5_ESM.zip › Figure 2/2C/Nedd4L x Clathrin x FCHO2.tif]

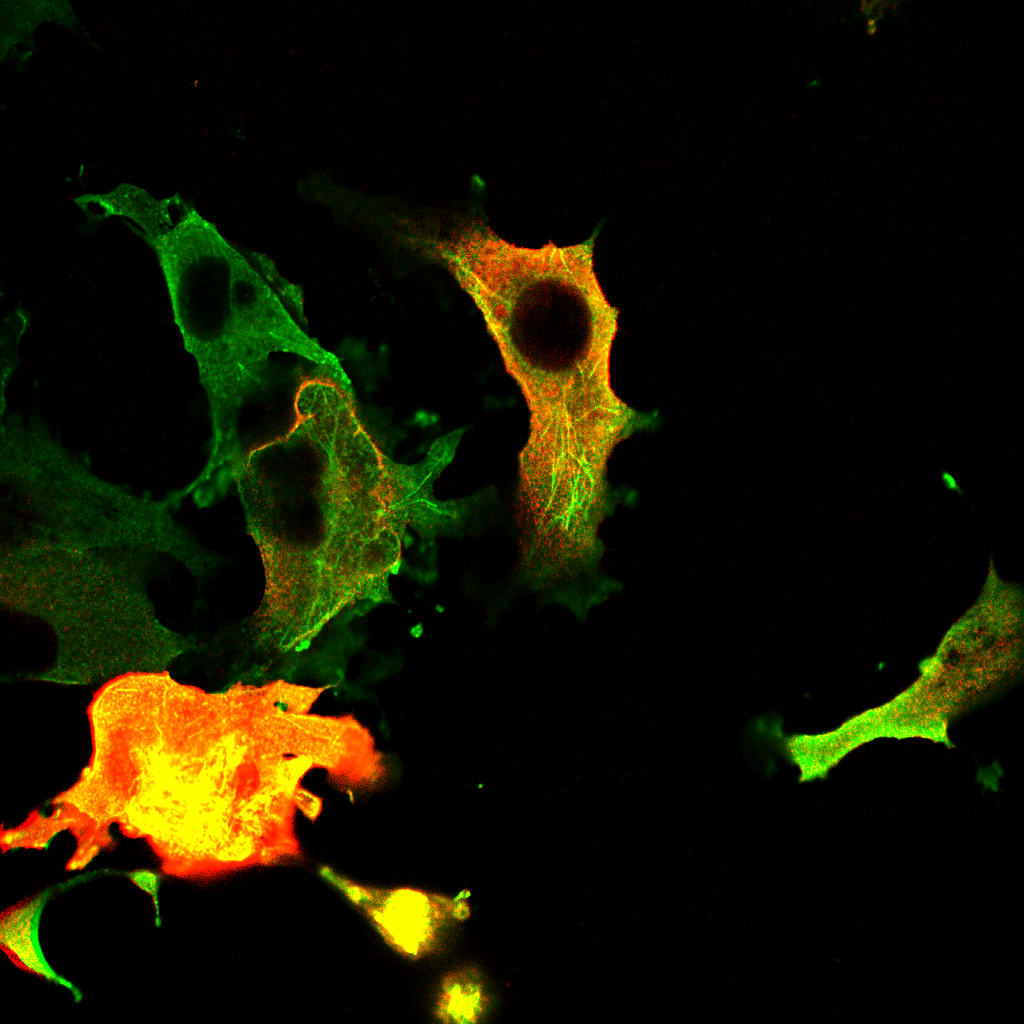

Supplement: Supplementary file 7 — Source data Fig. 4 [file 44318_2024_268_MOESM7_ESM.zip › EMBOJ-2023-114687_SourceDataForFigure4/4A/Amphiphysin x Nedd4L.tif]

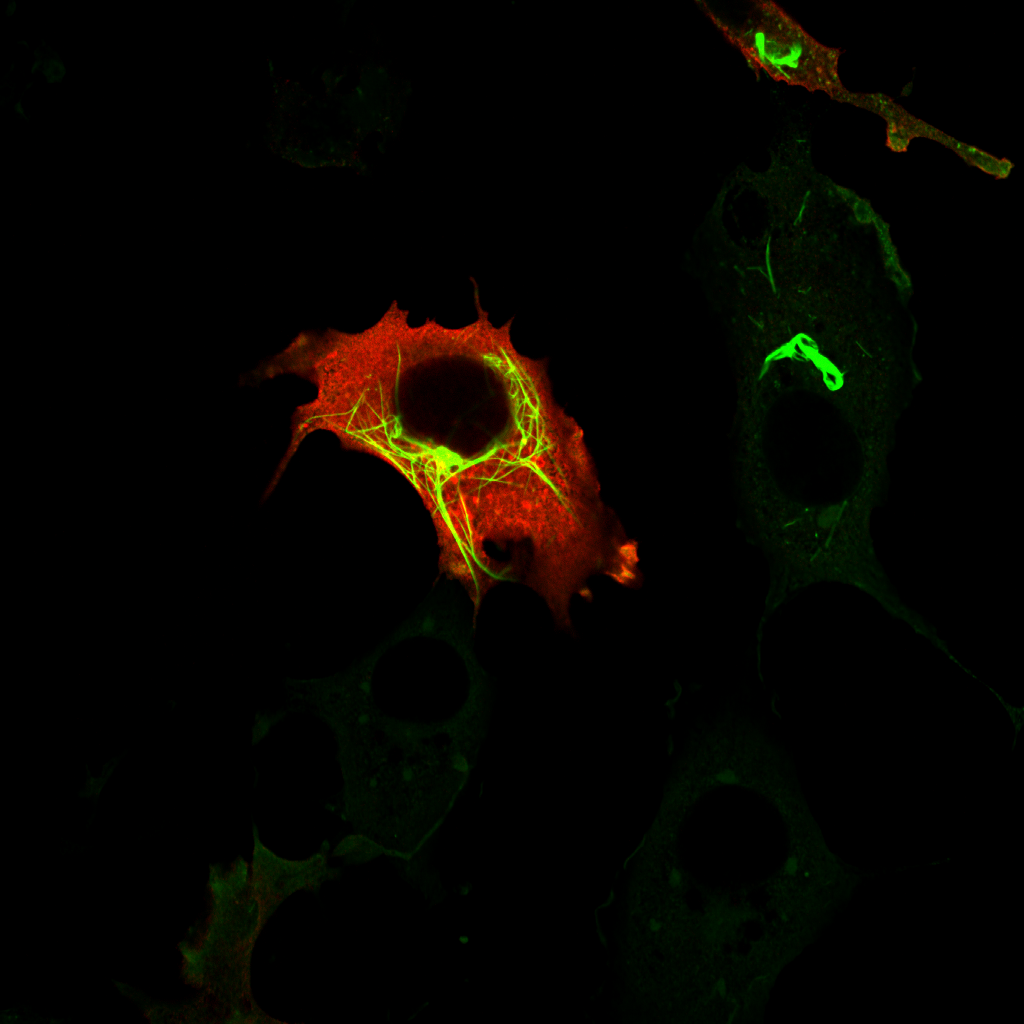

Supplement: Supplementary file 7 — Source data Fig. 4 [file 44318_2024_268_MOESM7_ESM.zip › EMBOJ-2023-114687_SourceDataForFigure4/4A/FBP17 x Nedd4L.tif]

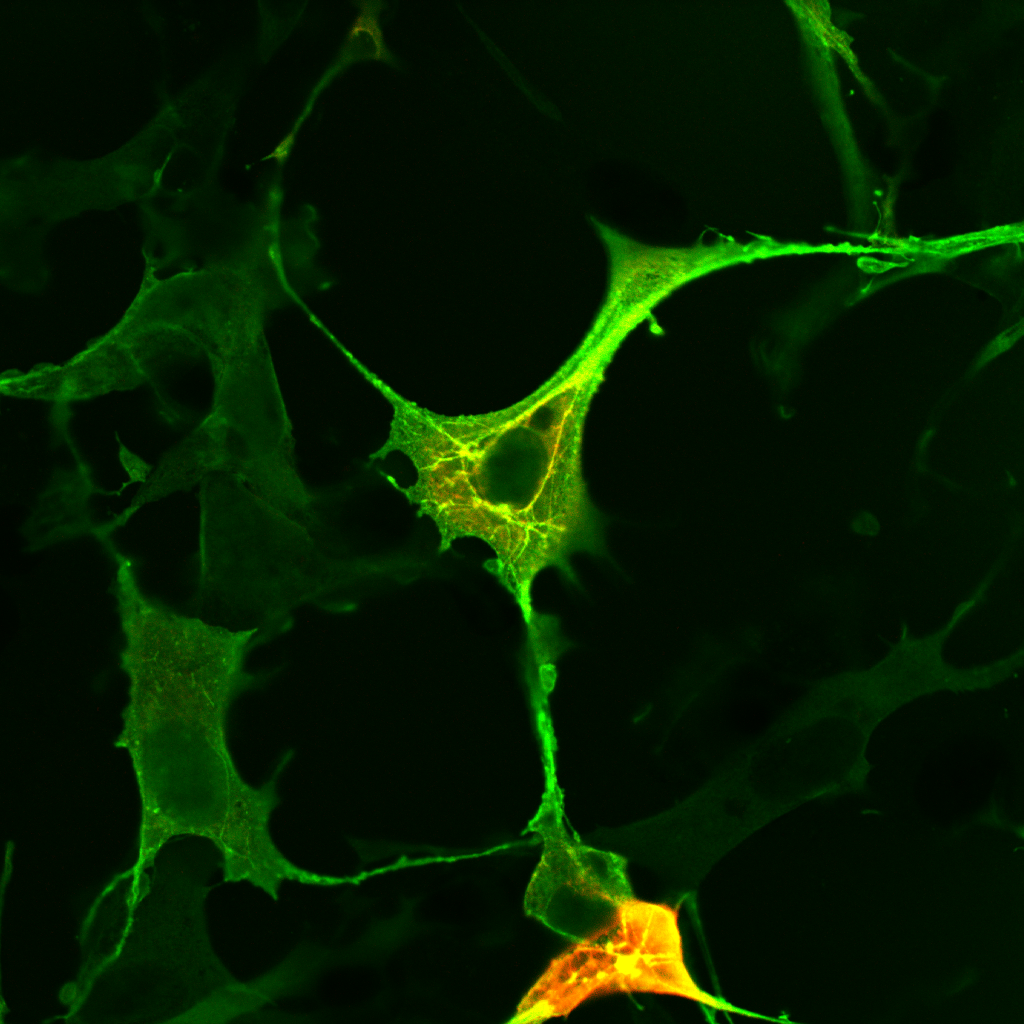

Supplement: Supplementary file 7 — Source data Fig. 4 [file 44318_2024_268_MOESM7_ESM.zip › EMBOJ-2023-114687_SourceDataForFigure4/4A/FCHO2 x Nedd4L.tif]

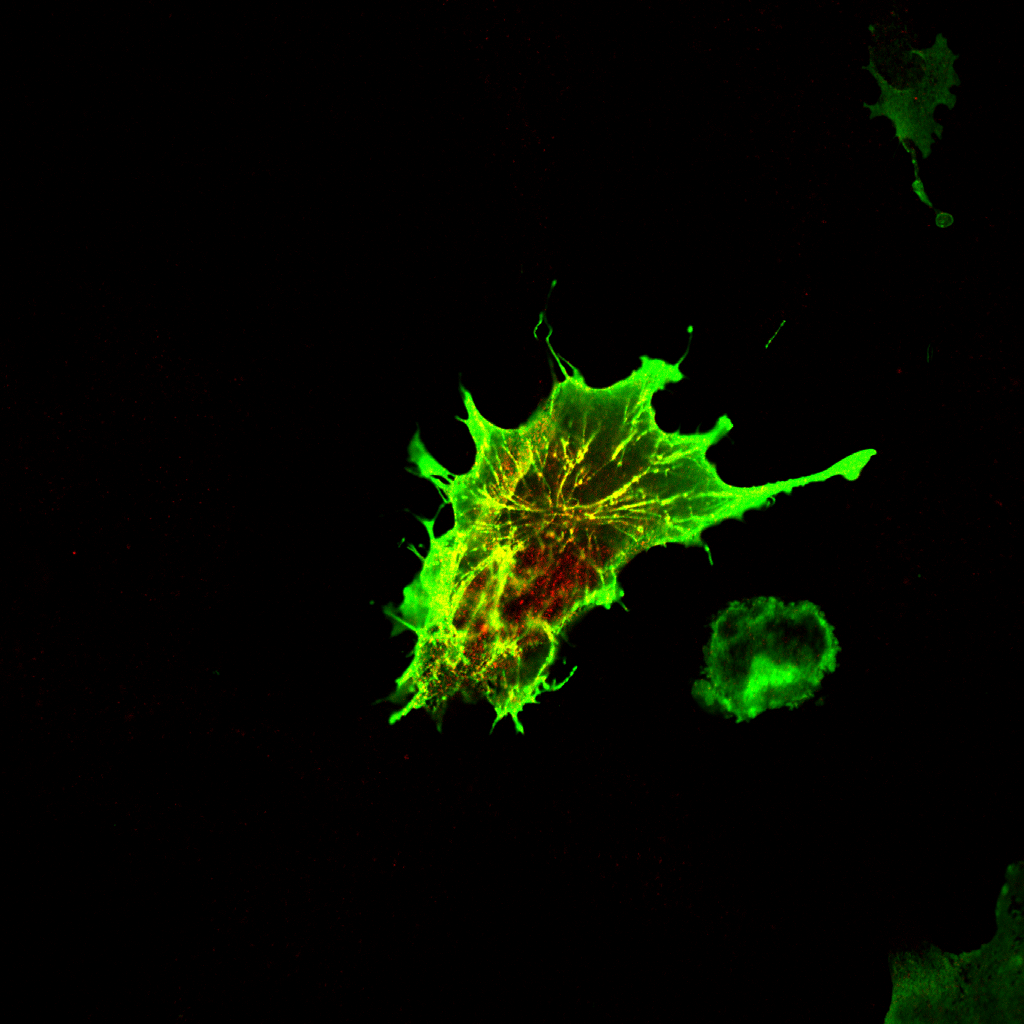

Supplement: Supplementary file 7 — Source data Fig. 4 [file 44318_2024_268_MOESM7_ESM.zip › EMBOJ-2023-114687_SourceDataForFigure4/4A/FCHO2 x Nedd4LC2.tif]

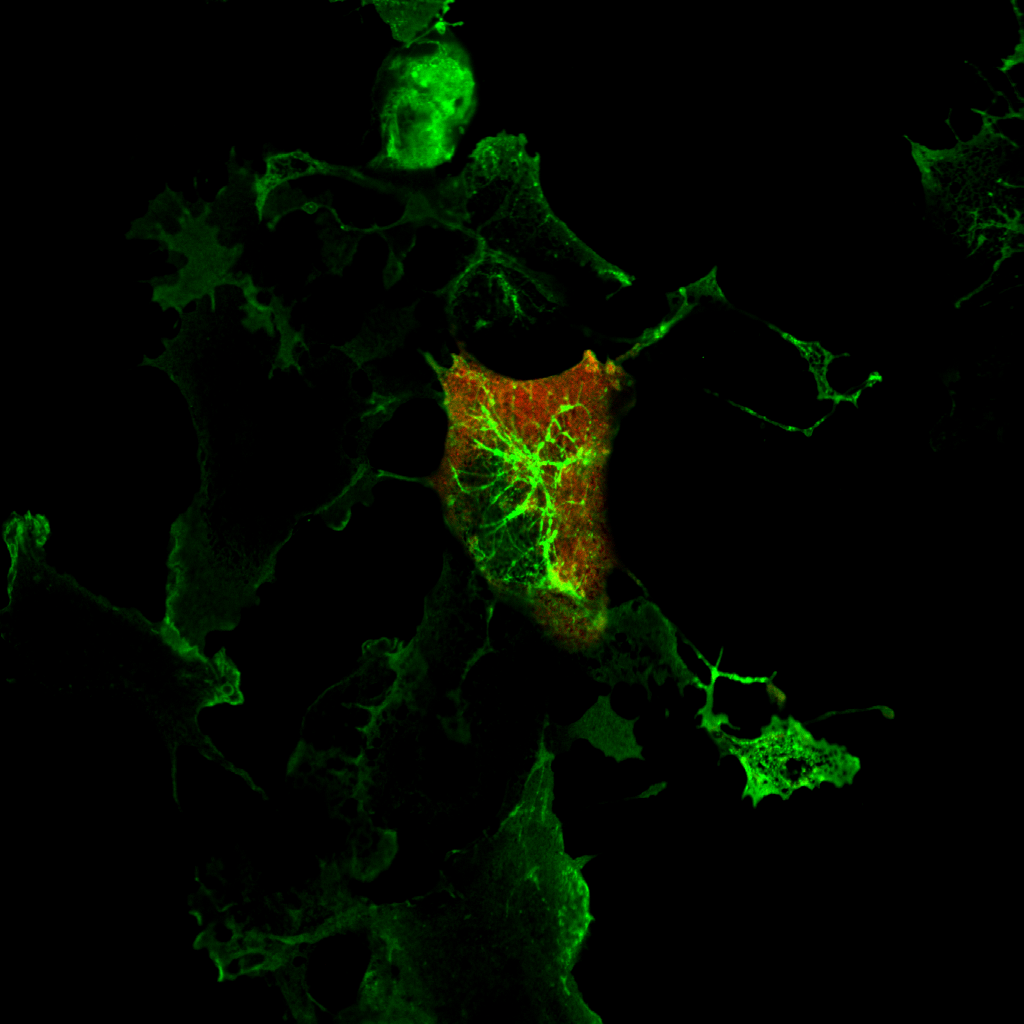

Supplement: Supplementary file 7 — Source data Fig. 4 [file 44318_2024_268_MOESM7_ESM.zip › EMBOJ-2023-114687_SourceDataForFigure4/4A/FCHO2 x Nedd4LdeltaC2.tif]

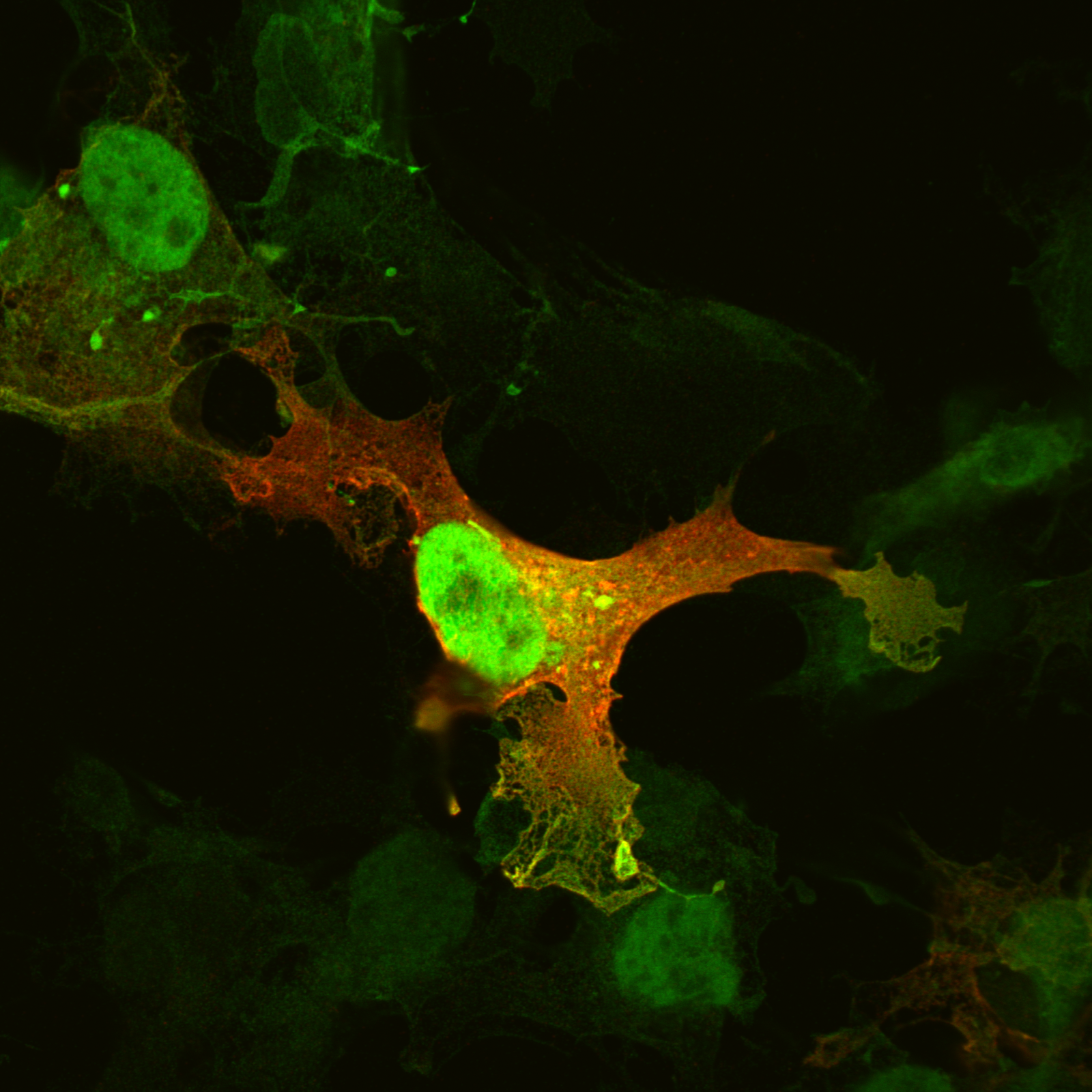

Supplement: Supplementary file 7 — Source data Fig. 4 [file 44318_2024_268_MOESM7_ESM.zip › EMBOJ-2023-114687_SourceDataForFigure4/4A/GFP x Nedd4L.tif]

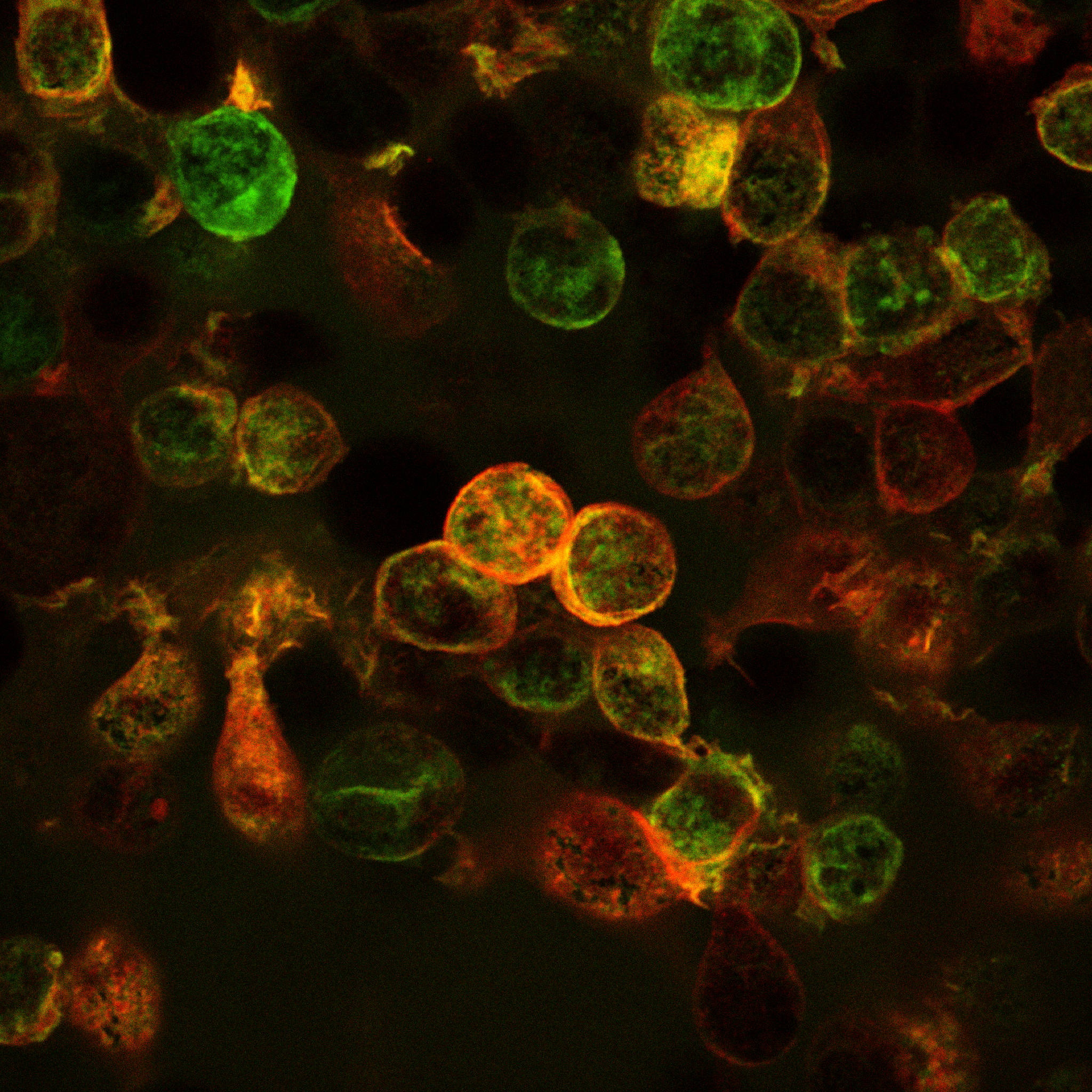

Supplement: Supplementary file 7 — Source data Fig. 4 [file 44318_2024_268_MOESM7_ESM.zip › EMBOJ-2023-114687_SourceDataForFigure4/4B/Amphiphysin x Nedd4L.tif]

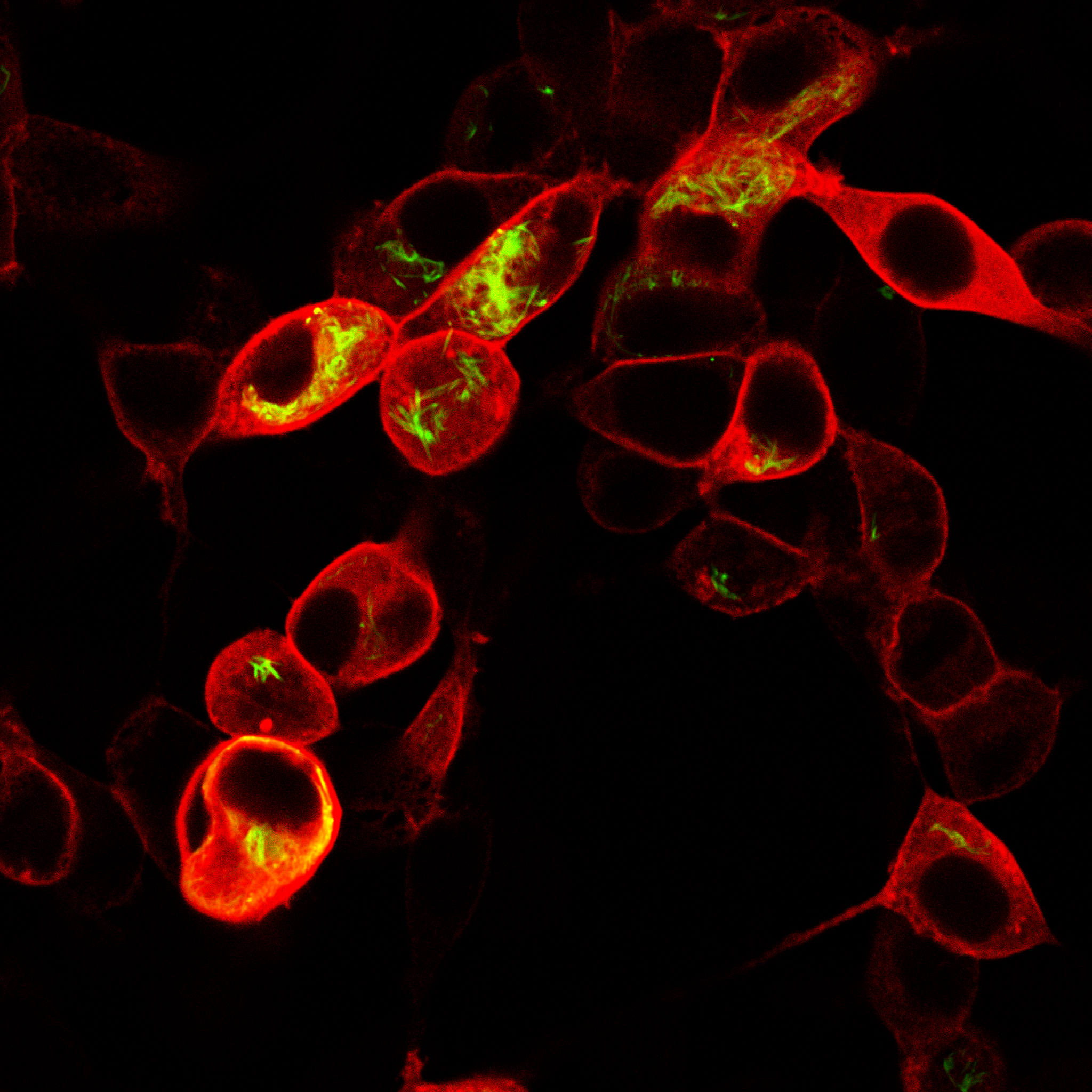

Supplement: Supplementary file 7 — Source data Fig. 4 [file 44318_2024_268_MOESM7_ESM.zip › EMBOJ-2023-114687_SourceDataForFigure4/4B/FBP17 x Nedd4L.tif]

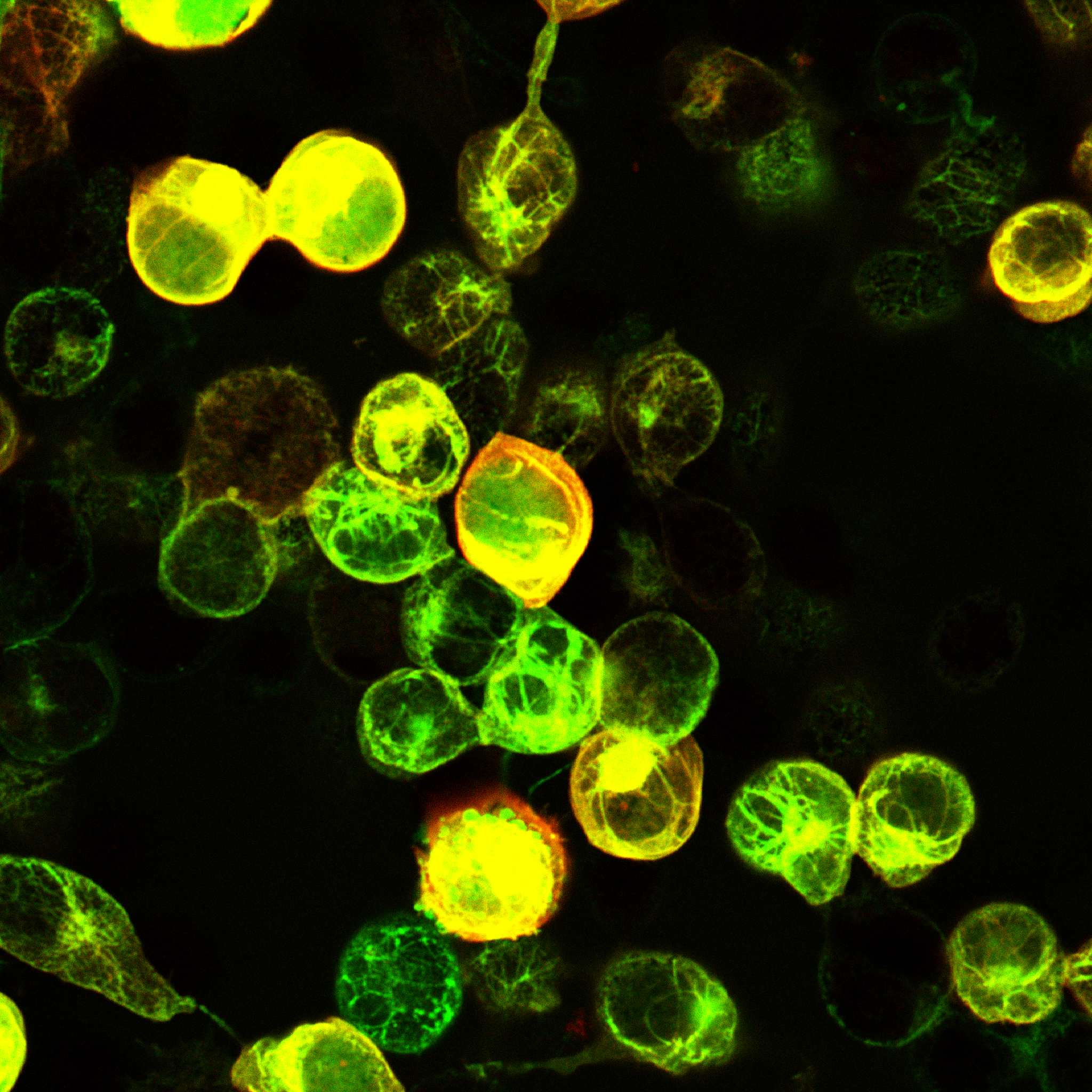

Supplement: Supplementary file 7 — Source data Fig. 4 [file 44318_2024_268_MOESM7_ESM.zip › EMBOJ-2023-114687_SourceDataForFigure4/4B/FCHO2 x Nedd4L.tif]

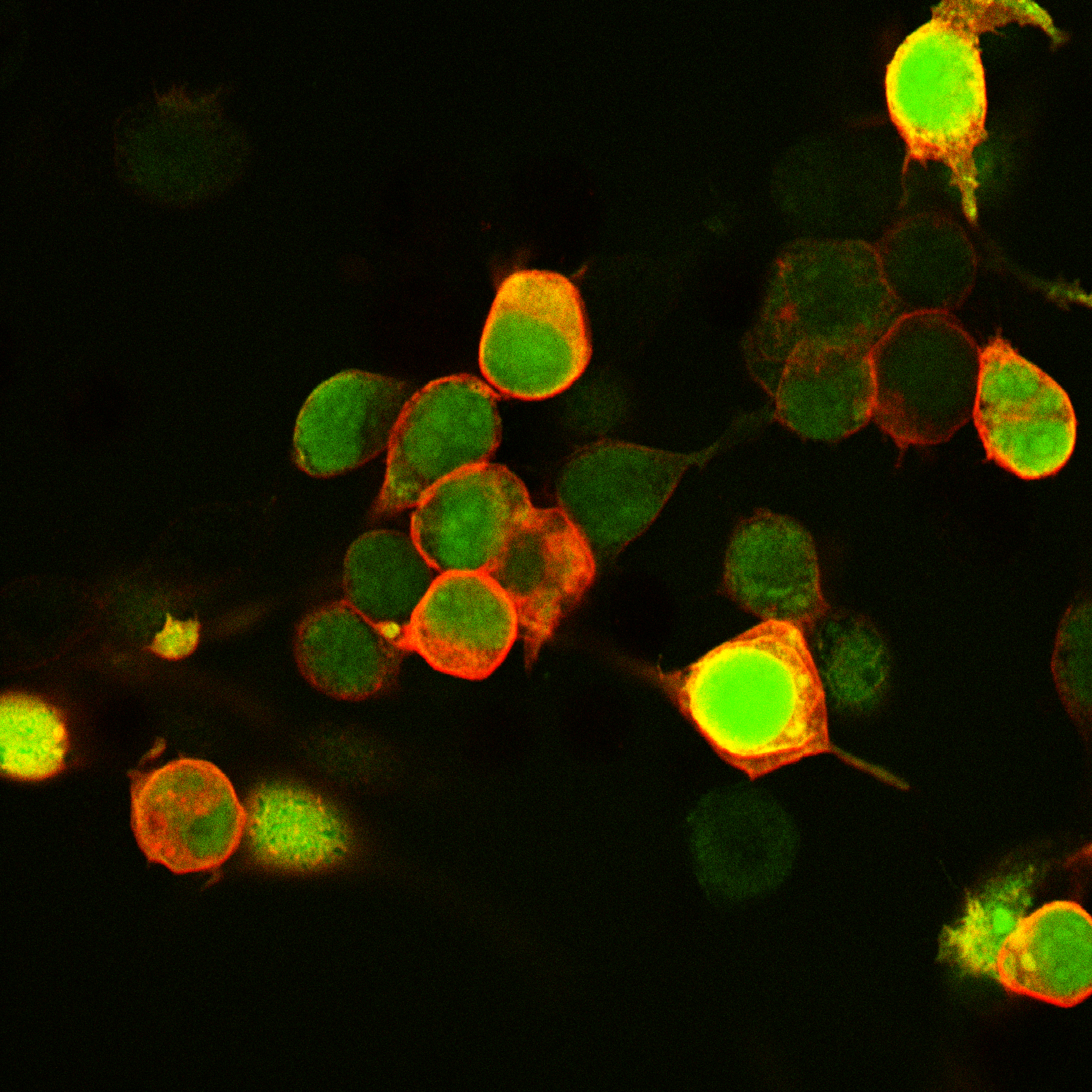

Supplement: Supplementary file 7 — Source data Fig. 4 [file 44318_2024_268_MOESM7_ESM.zip › EMBOJ-2023-114687_SourceDataForFigure4/4B/GFP x Nedd4L.tif]

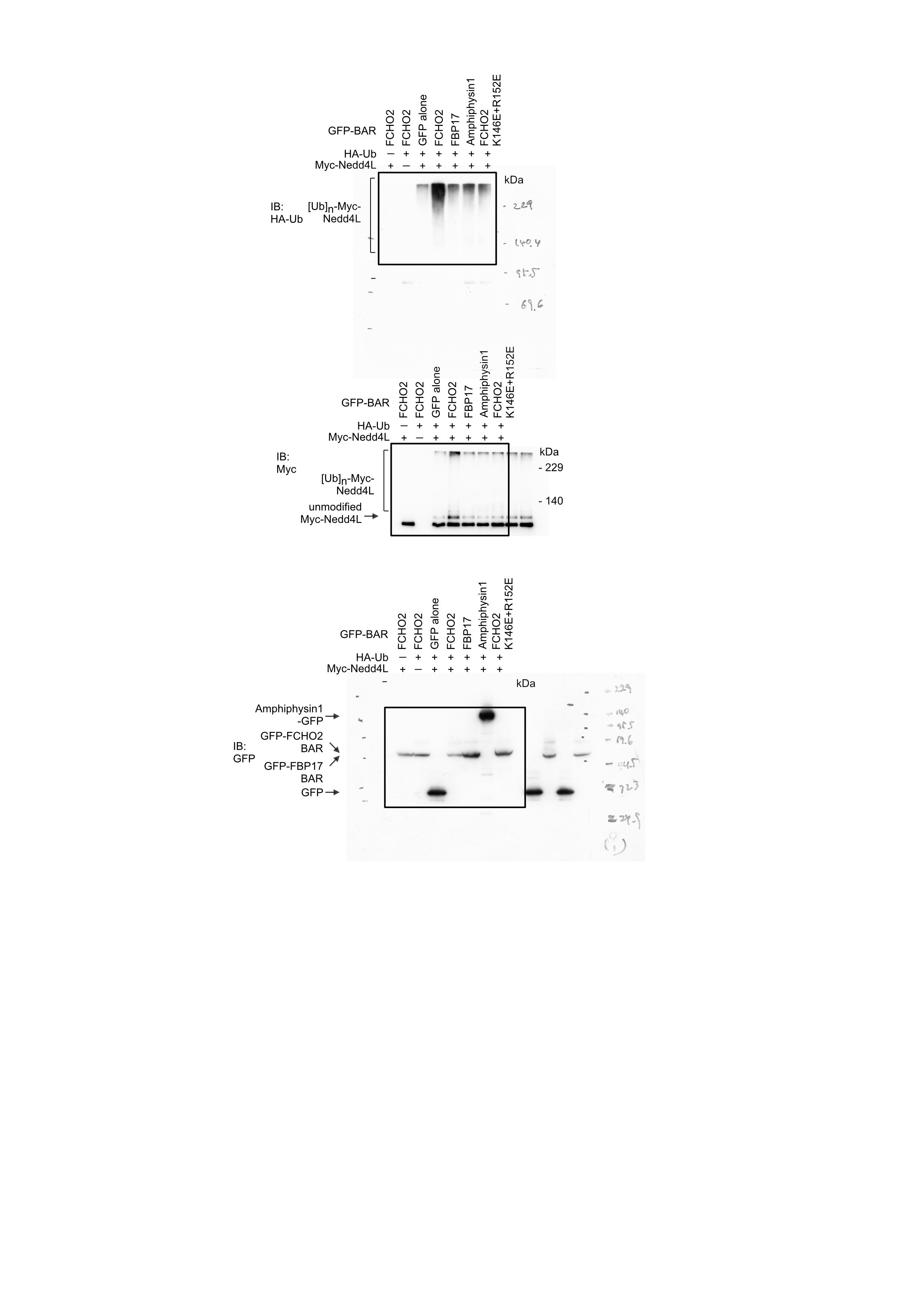

Supplement: Supplementary file 8 — Source data Fig. 5 [file 44318_2024_268_MOESM8_ESM.zip › Figure 5/5A/Fig5A.tiff]

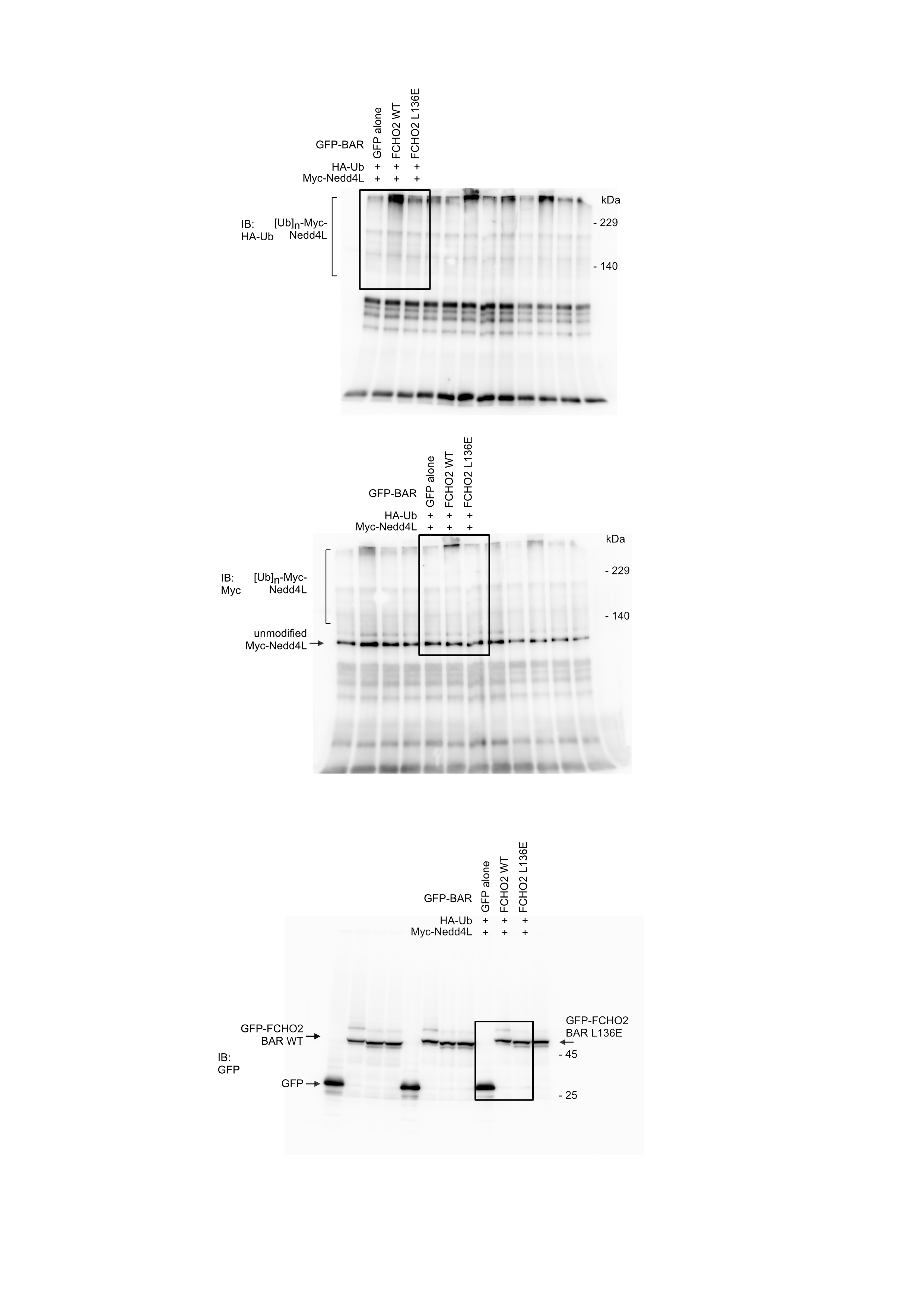

Supplement: Supplementary file 8 — Source data Fig. 5 [file 44318_2024_268_MOESM8_ESM.zip › Figure 5/5B/Fig5B.tiff]

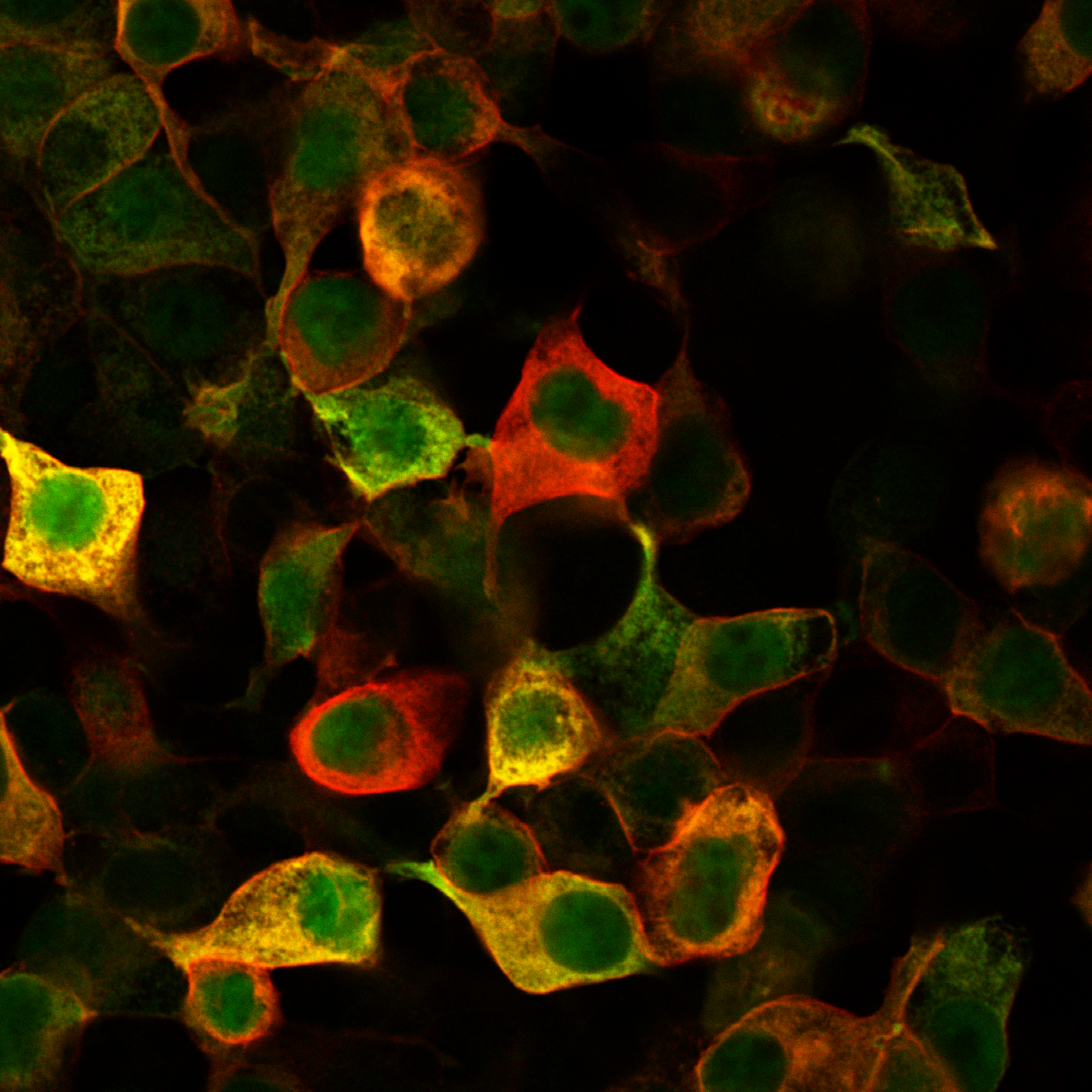

Supplement: Supplementary file 8 — Source data Fig. 5 [file 44318_2024_268_MOESM8_ESM.zip › Figure 5/5C/FCHO2K146ER152E x Nedd4L.tif]

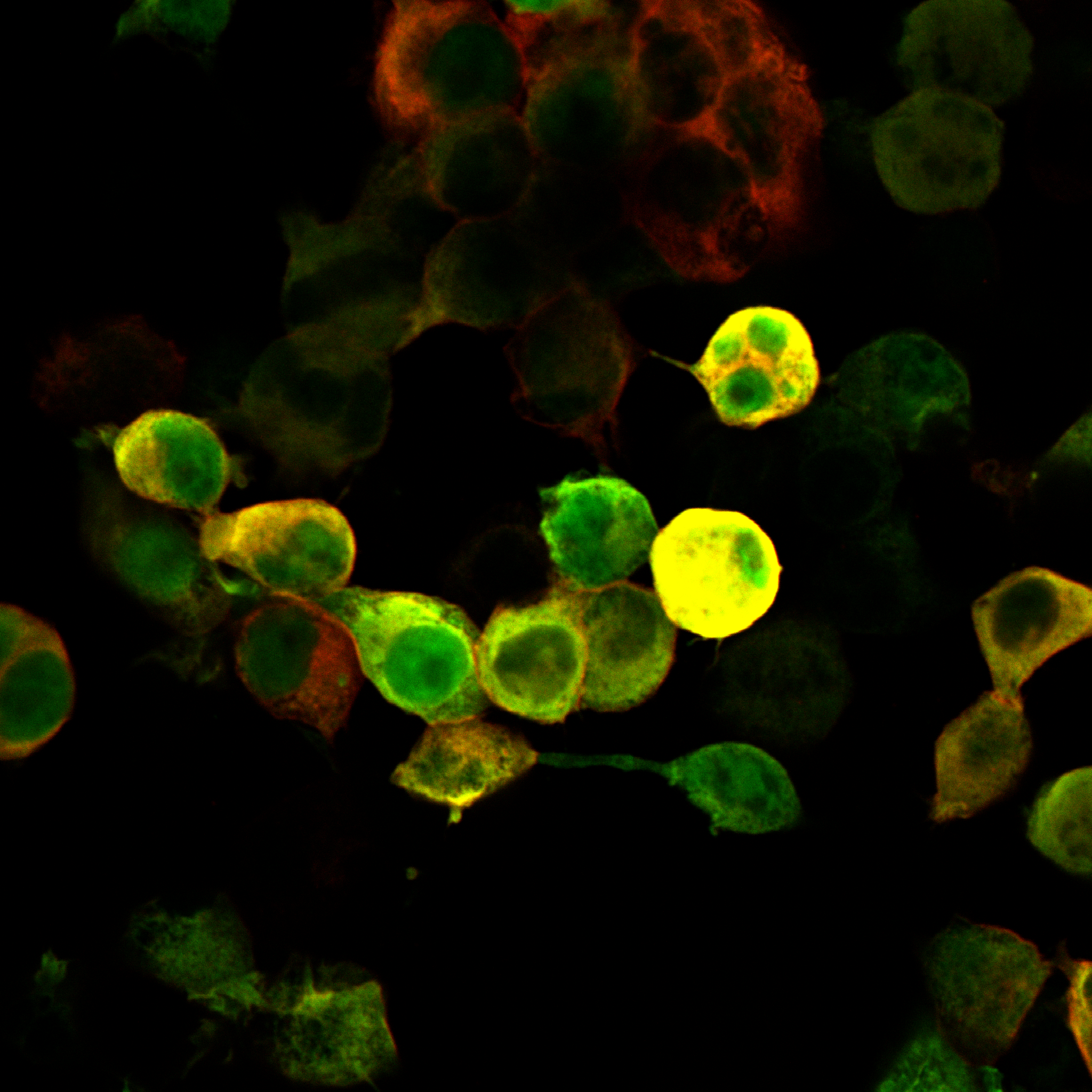

Supplement: Supplementary file 8 — Source data Fig. 5 [file 44318_2024_268_MOESM8_ESM.zip › Figure 5/5C/FCHO2L136E x Nedd4L.tif]

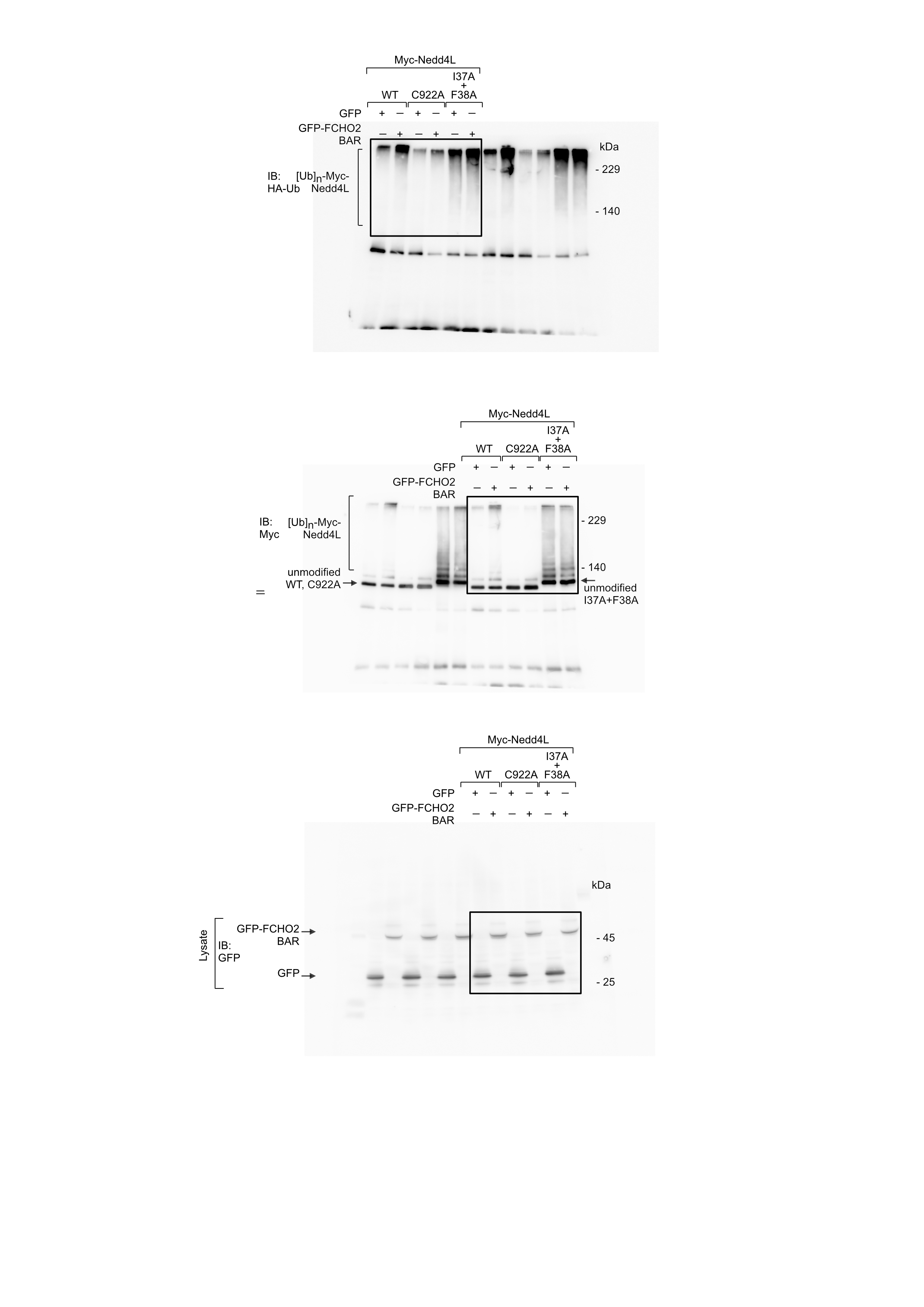

Supplement: Supplementary file 8 — Source data Fig. 5 [file 44318_2024_268_MOESM8_ESM.zip › Figure 5/5D/Fig5D.tiff]

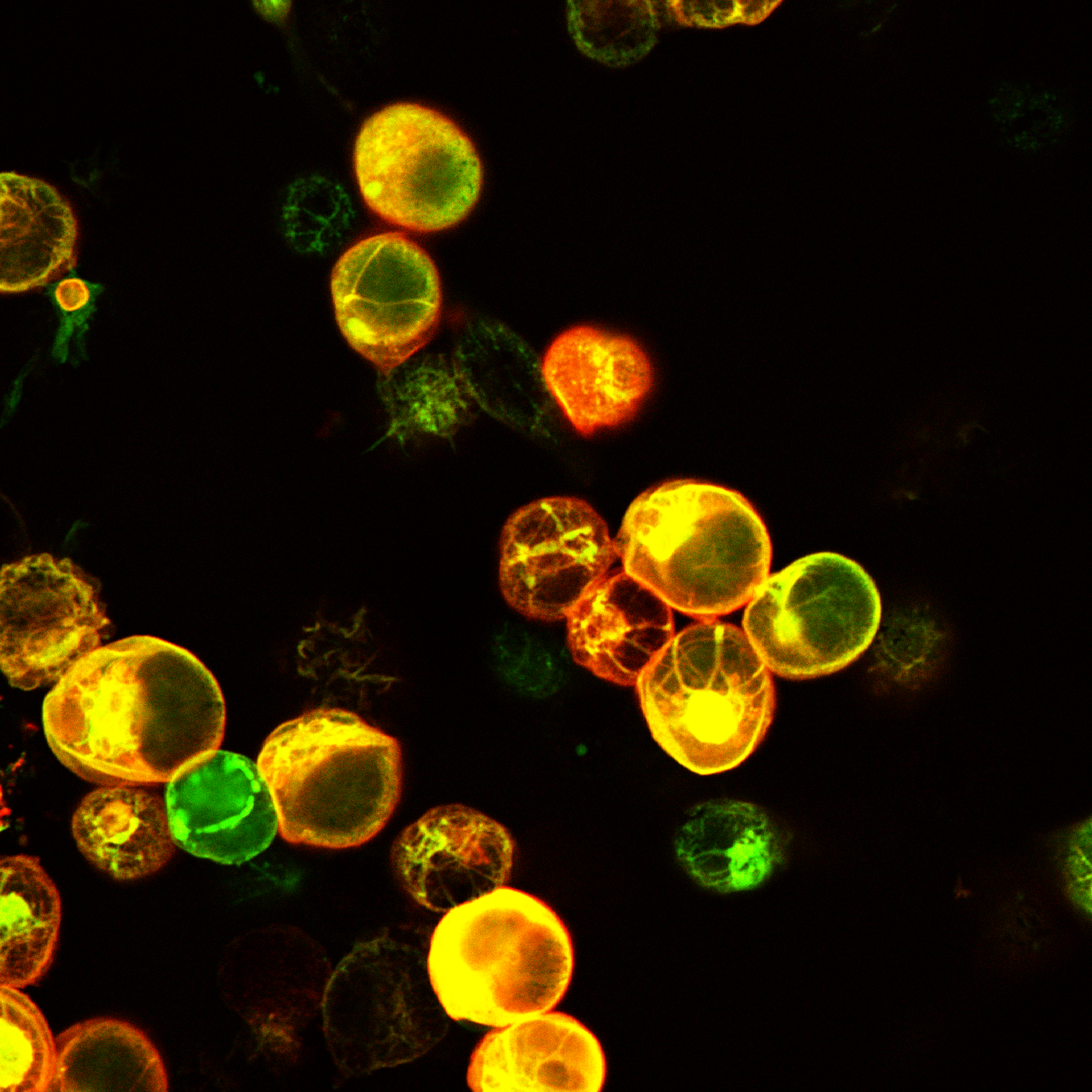

Supplement: Supplementary file 8 — Source data Fig. 5 [file 44318_2024_268_MOESM8_ESM.zip › Figure 5/5E/FCHO2 x Nedd4LC922A.tif (RGB).tif]

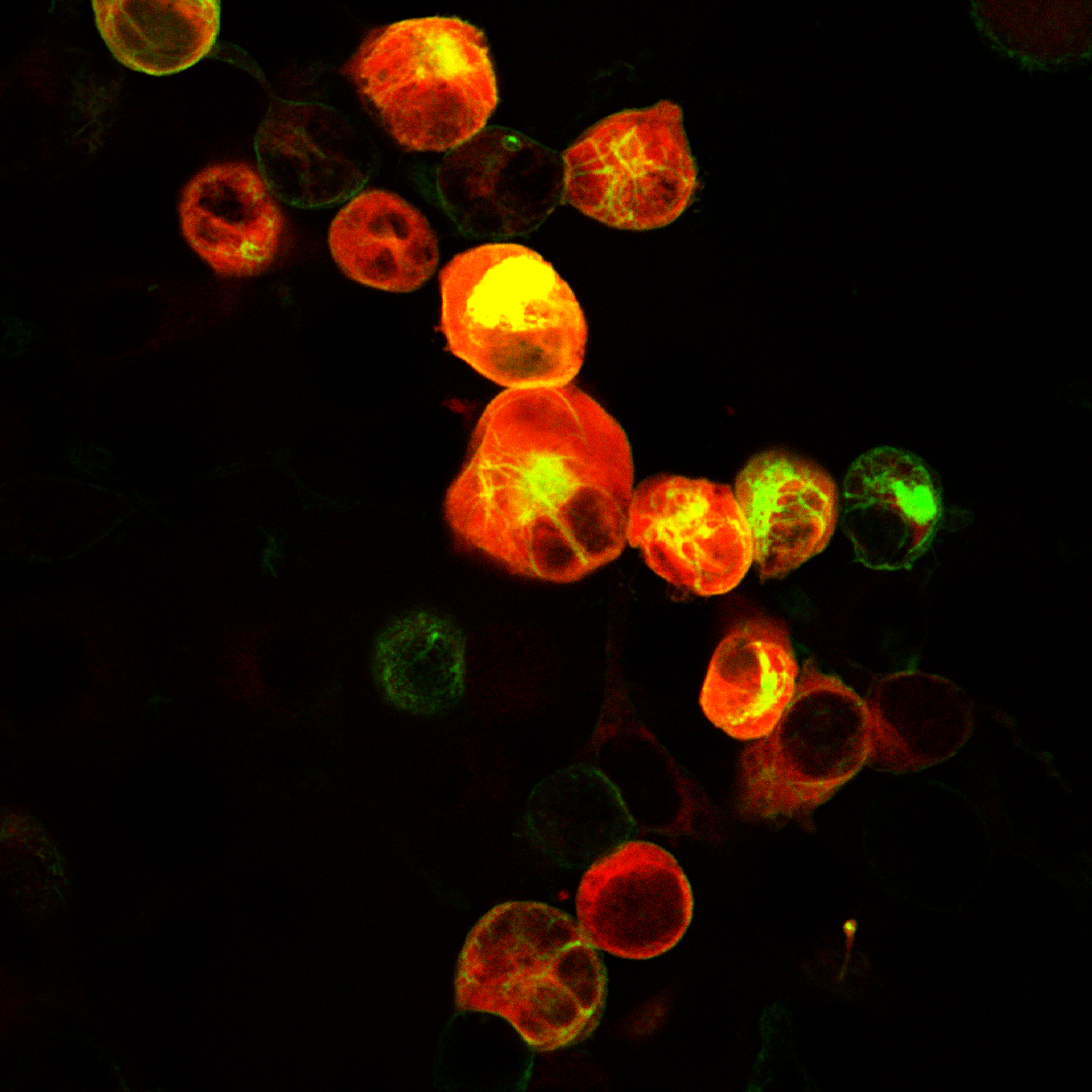

Supplement: Supplementary file 8 — Source data Fig. 5 [file 44318_2024_268_MOESM8_ESM.zip › Figure 5/5E/FCHO2 x Nedd4LI37AF38A.tif]

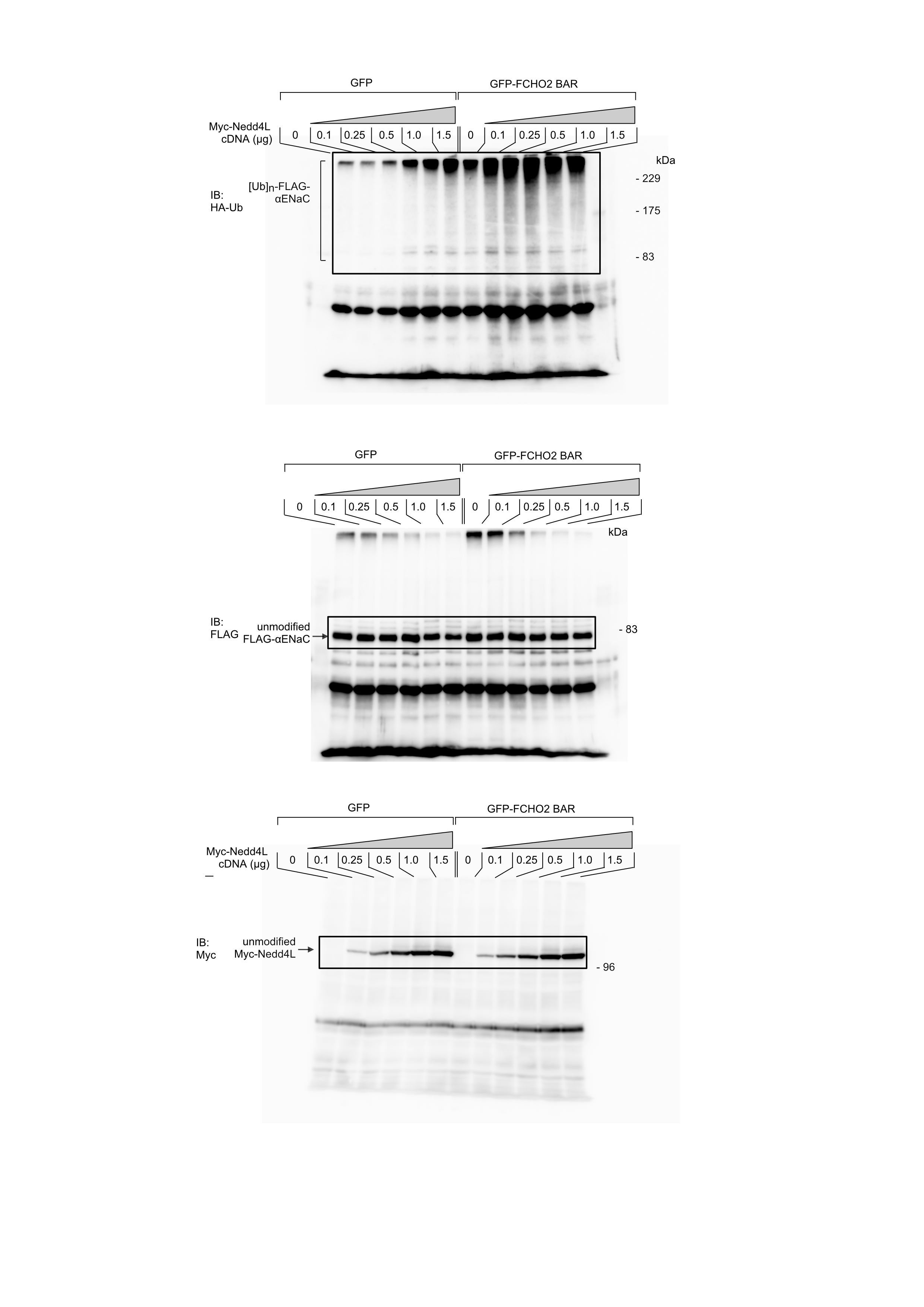

Supplement: Supplementary file 8 — Source data Fig. 5 [file 44318_2024_268_MOESM8_ESM.zip › Figure 5/5F/Fig5F_1.tiff]

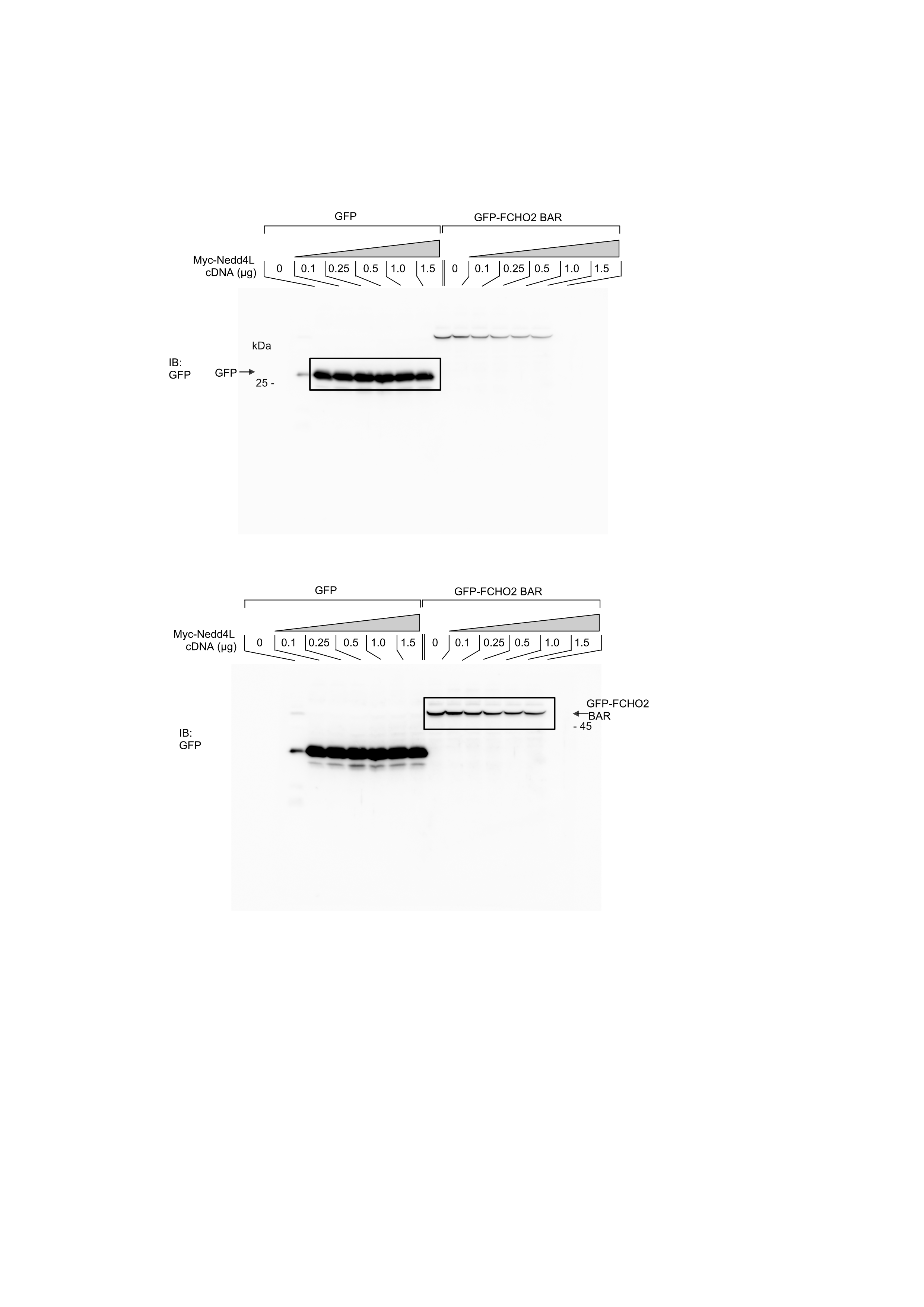

Supplement: Supplementary file 8 — Source data Fig. 5 [file 44318_2024_268_MOESM8_ESM.zip › Figure 5/5F/Fig5F_2.tiff]

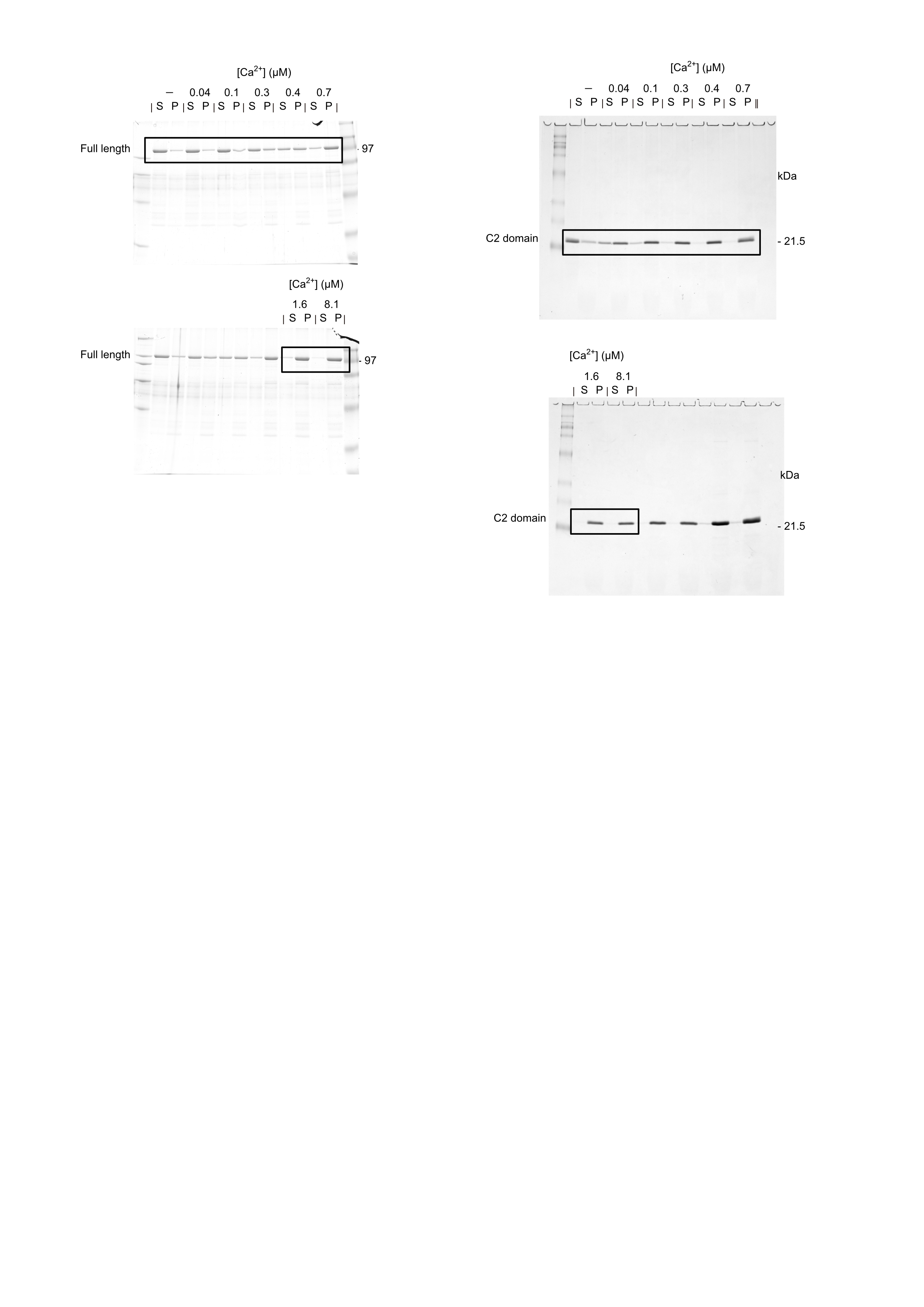

Supplement: Supplementary file 9 — Source data Fig. 6 [file 44318_2024_268_MOESM9_ESM.zip › Figure 6/6A/Fig6A.tiff]

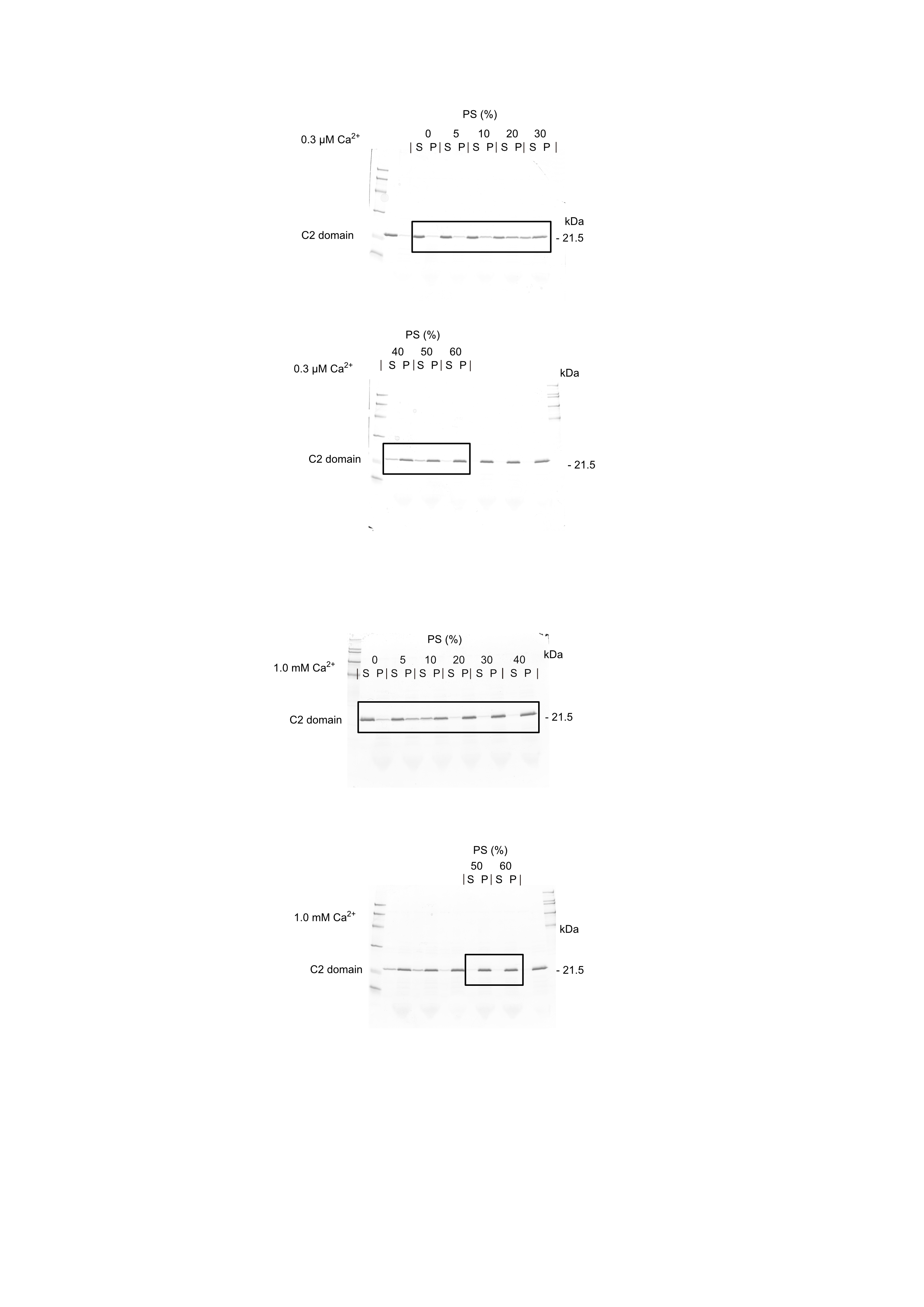

Supplement: Supplementary file 9 — Source data Fig. 6 [file 44318_2024_268_MOESM9_ESM.zip › Figure 6/6B/Fig6B.tiff]

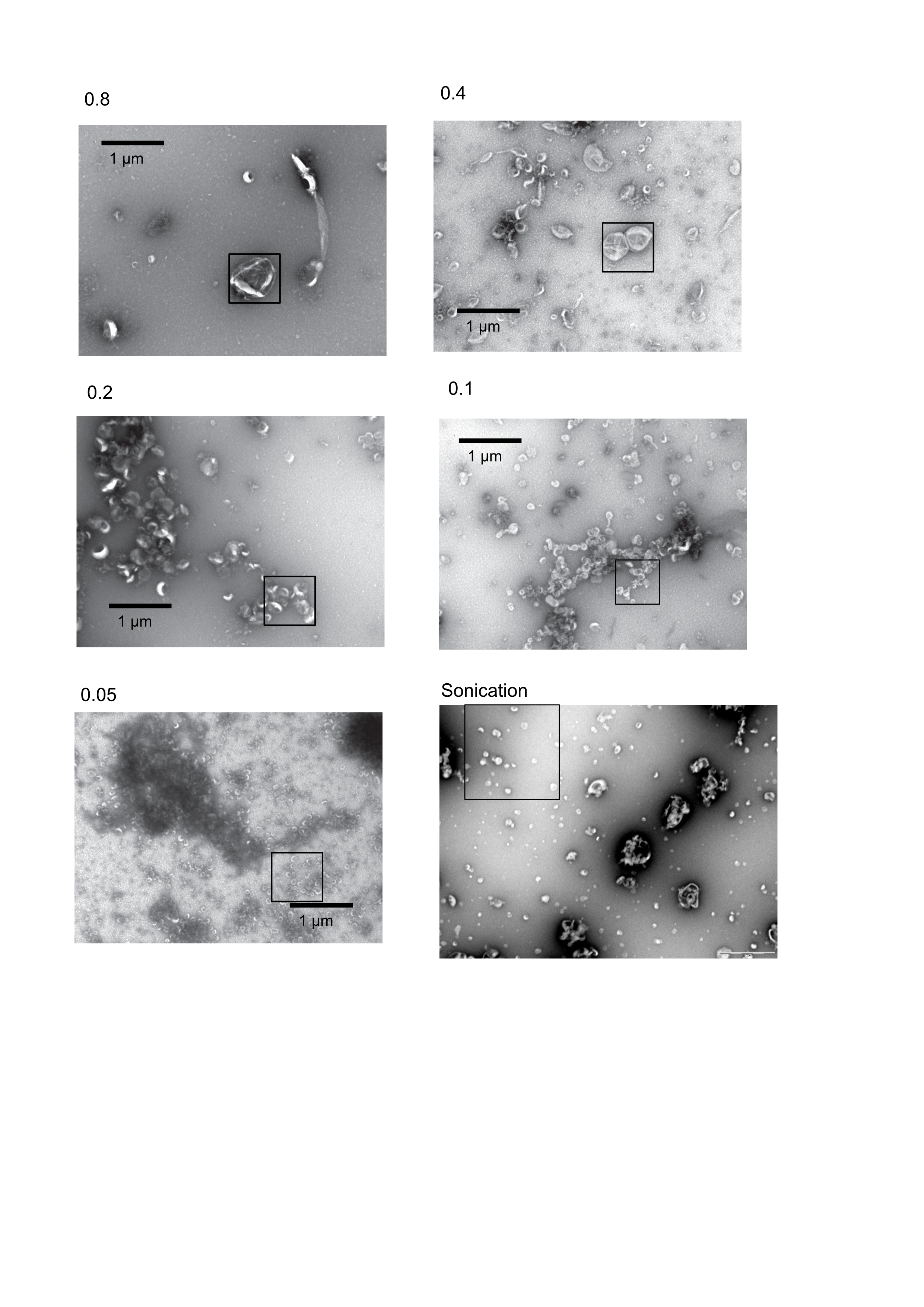

Supplement: Supplementary file 10 — Source data Fig. 7 [file 44318_2024_268_MOESM10_ESM.zip › EMBOJ-2023-114687R2_SourceDataForFig7/7A/Fig7A.tiff]

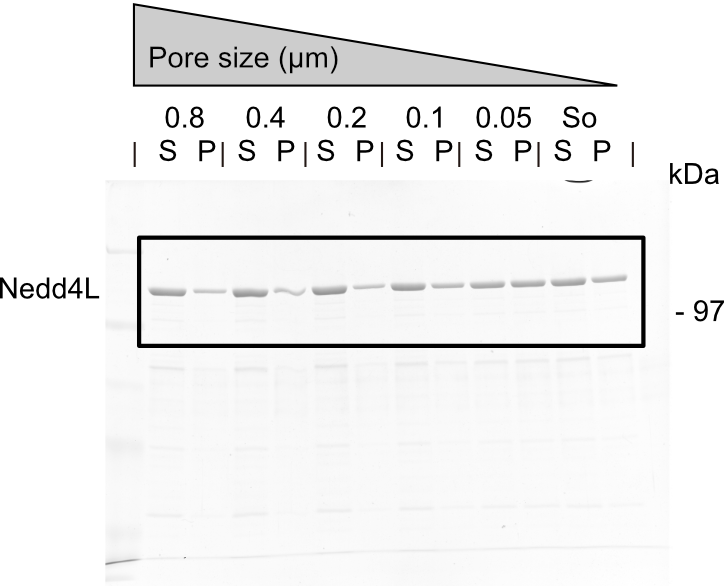

Supplement: Supplementary file 10 — Source data Fig. 7 [file 44318_2024_268_MOESM10_ESM.zip › EMBOJ-2023-114687R2_SourceDataForFig7/7B/Fig7B.tiff]

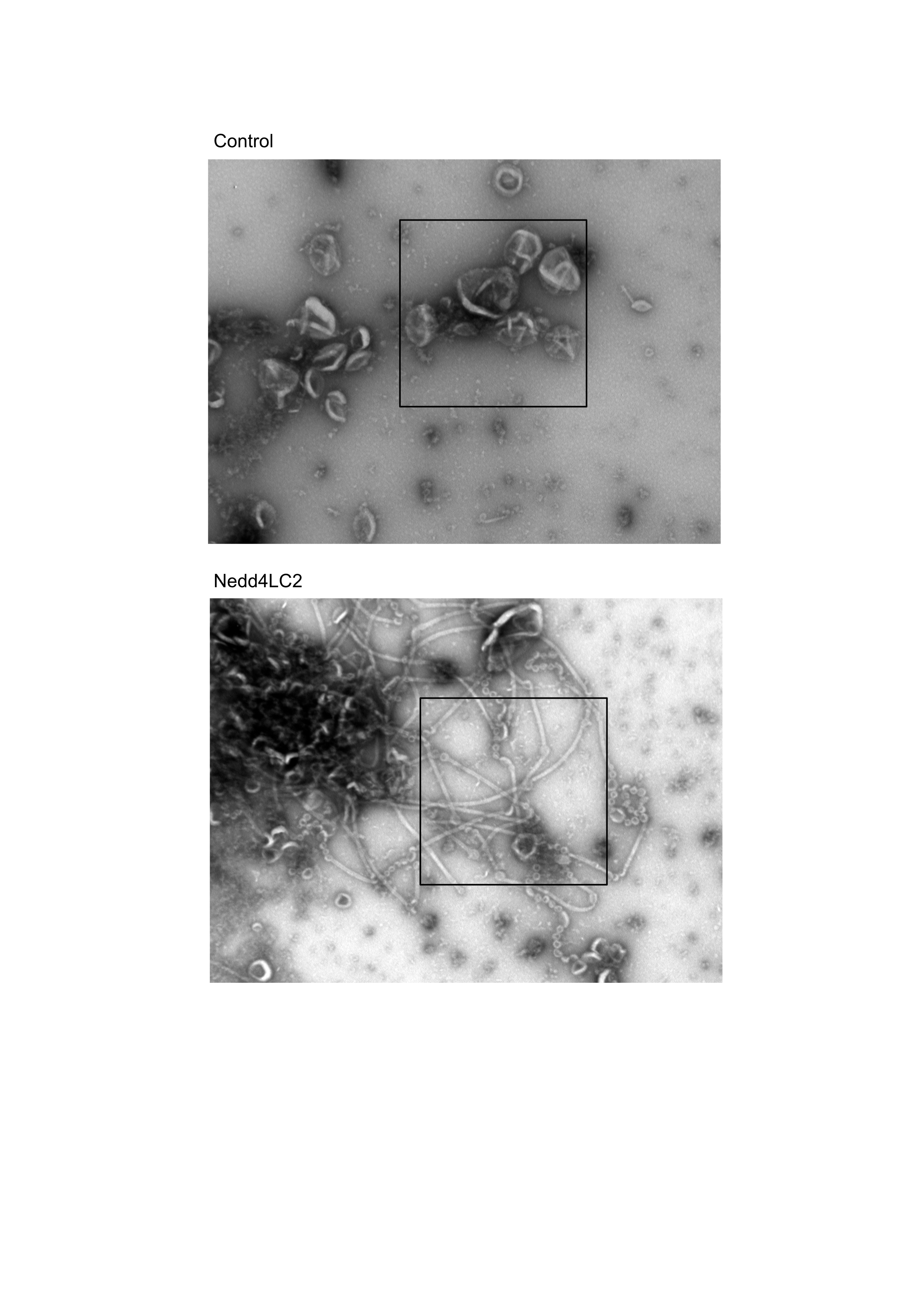

Supplement: Supplementary file 10 — Source data Fig. 7 [file 44318_2024_268_MOESM10_ESM.zip › EMBOJ-2023-114687R2_SourceDataForFig7/7C/Fig7C.tiff]

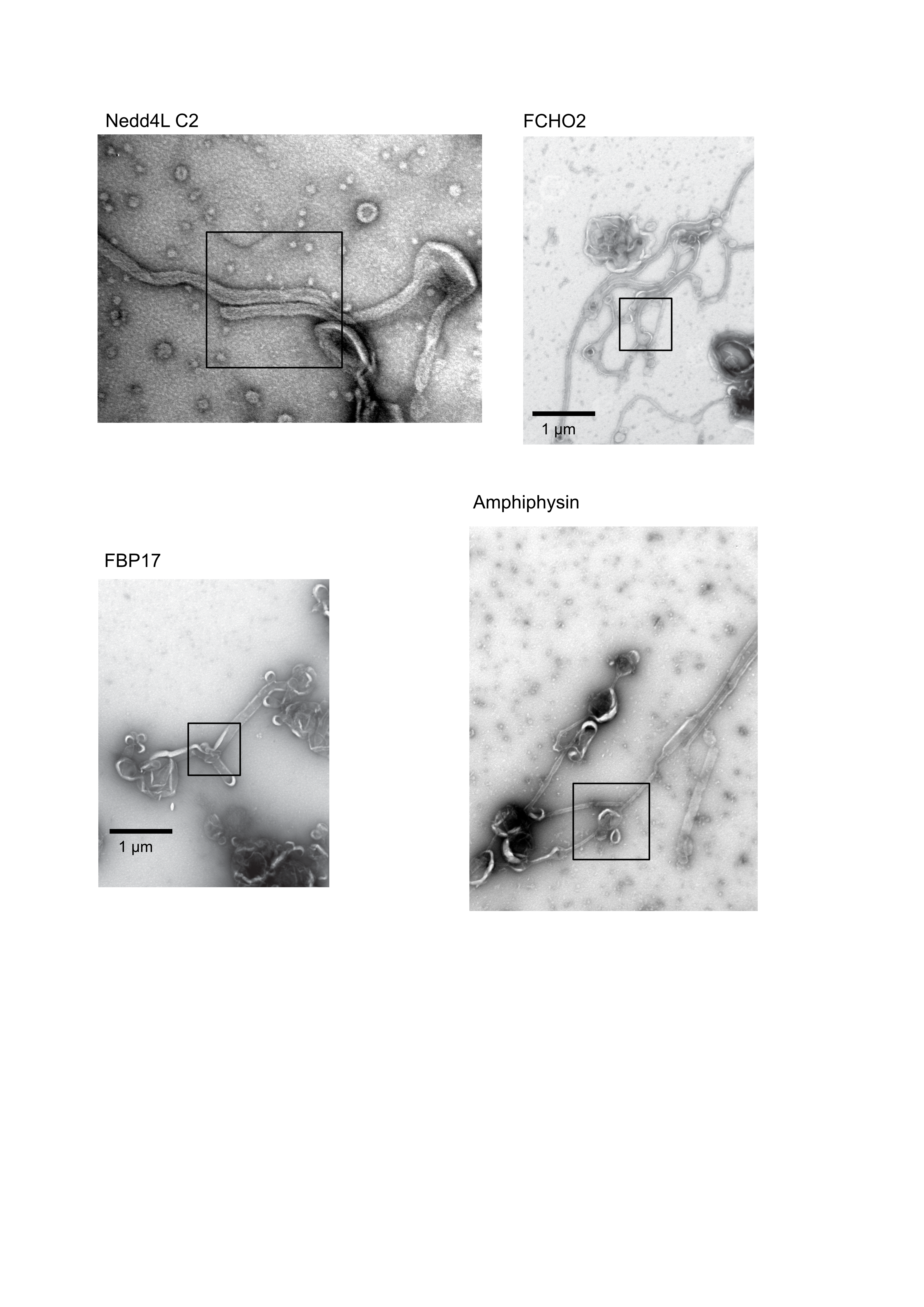

Supplement: Supplementary file 10 — Source data Fig. 7 [file 44318_2024_268_MOESM10_ESM.zip › EMBOJ-2023-114687R2_SourceDataForFig7/7D/Fig7D.tiff]

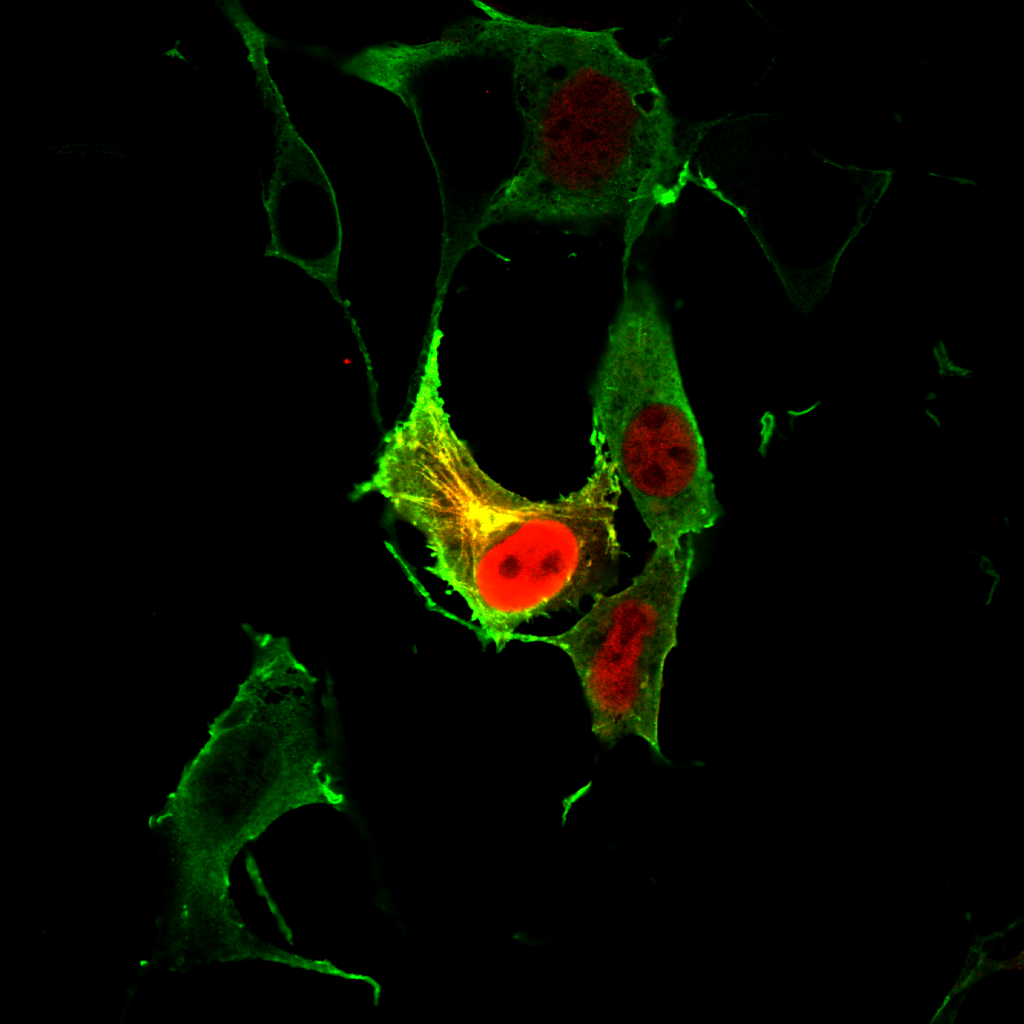

Supplement: Supplementary file 11 — Source data Fig. 8 [file 44318_2024_268_MOESM11_ESM.zip › Figure 8/8B/FCHO2 x Nedd4LC2F38A.tif]

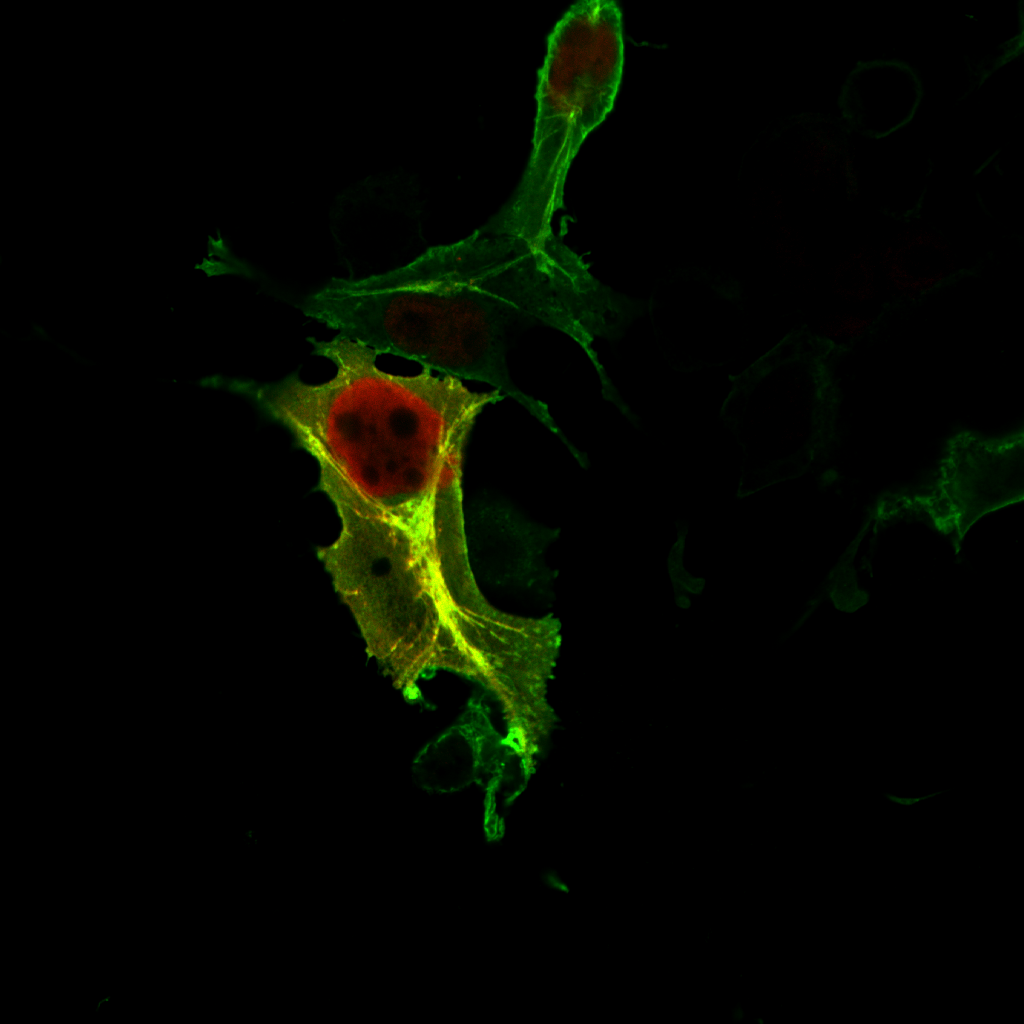

Supplement: Supplementary file 11 — Source data Fig. 8 [file 44318_2024_268_MOESM11_ESM.zip › Figure 8/8B/FCHO2 x Nedd4LC2I37A.tif]

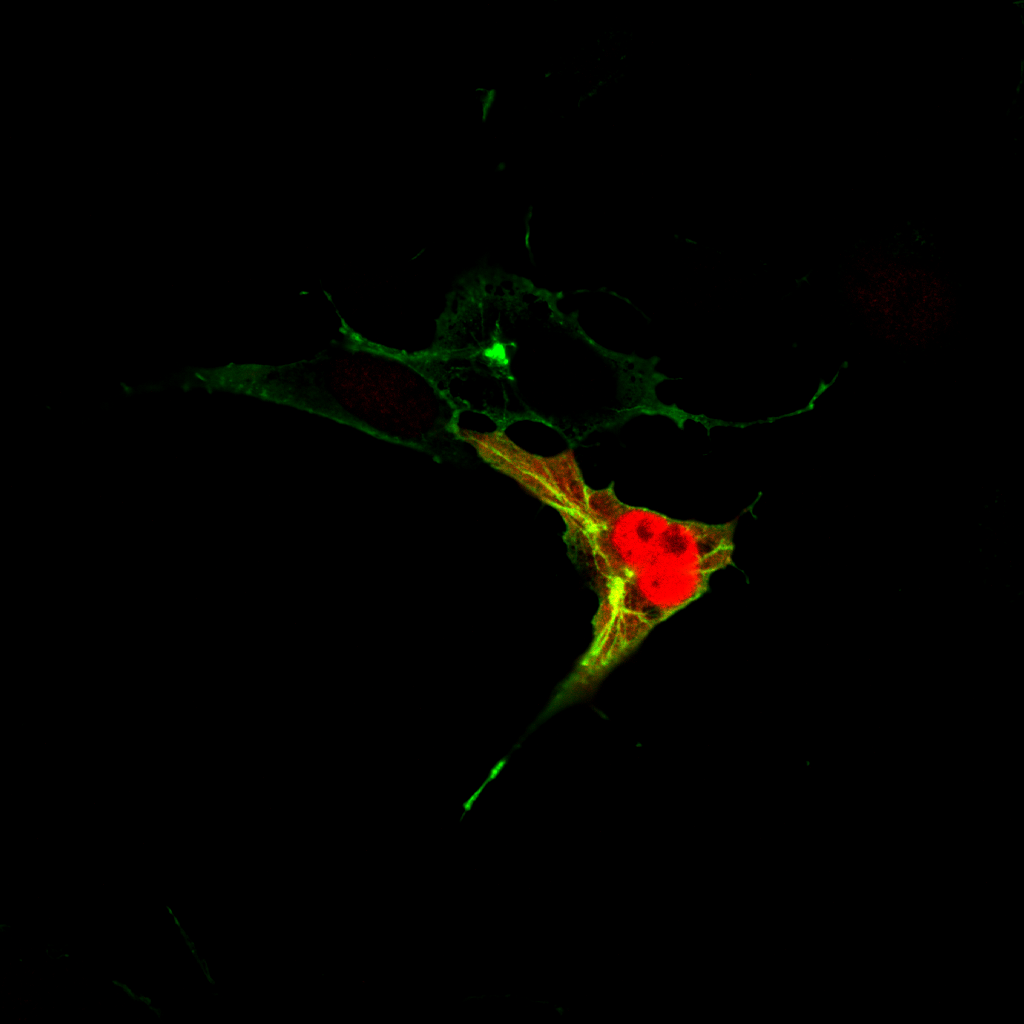

Supplement: Supplementary file 11 — Source data Fig. 8 [file 44318_2024_268_MOESM11_ESM.zip › Figure 8/8B/FCHO2 x Nedd4LC2I37AF38A.tif]

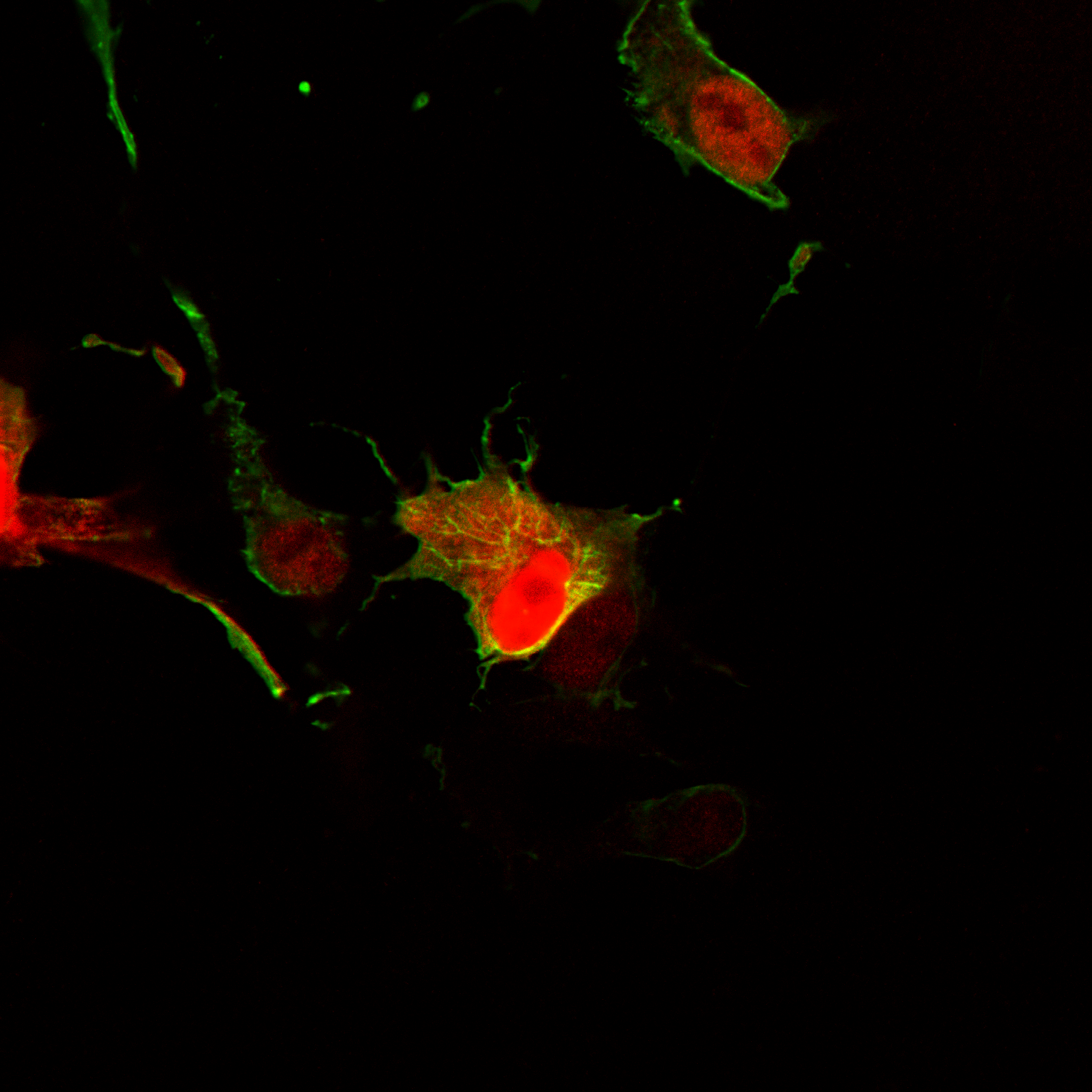

Supplement: Supplementary file 11 — Source data Fig. 8 [file 44318_2024_268_MOESM11_ESM.zip › Figure 8/8B/FCHO2 x Nedd4LC2I37AL99A.tif]

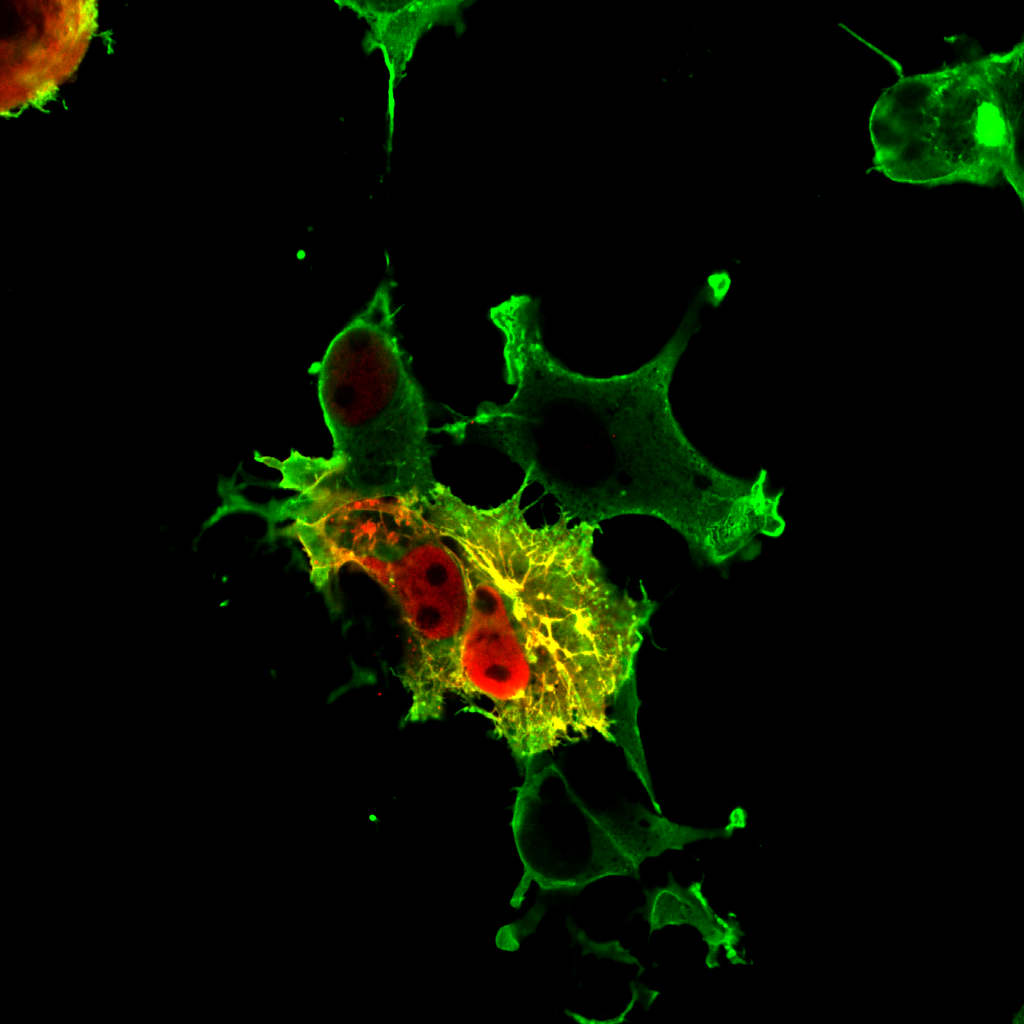

Supplement: Supplementary file 11 — Source data Fig. 8 [file 44318_2024_268_MOESM11_ESM.zip › Figure 8/8B/FCHO2 x Nedd4LC2L99A.tif]

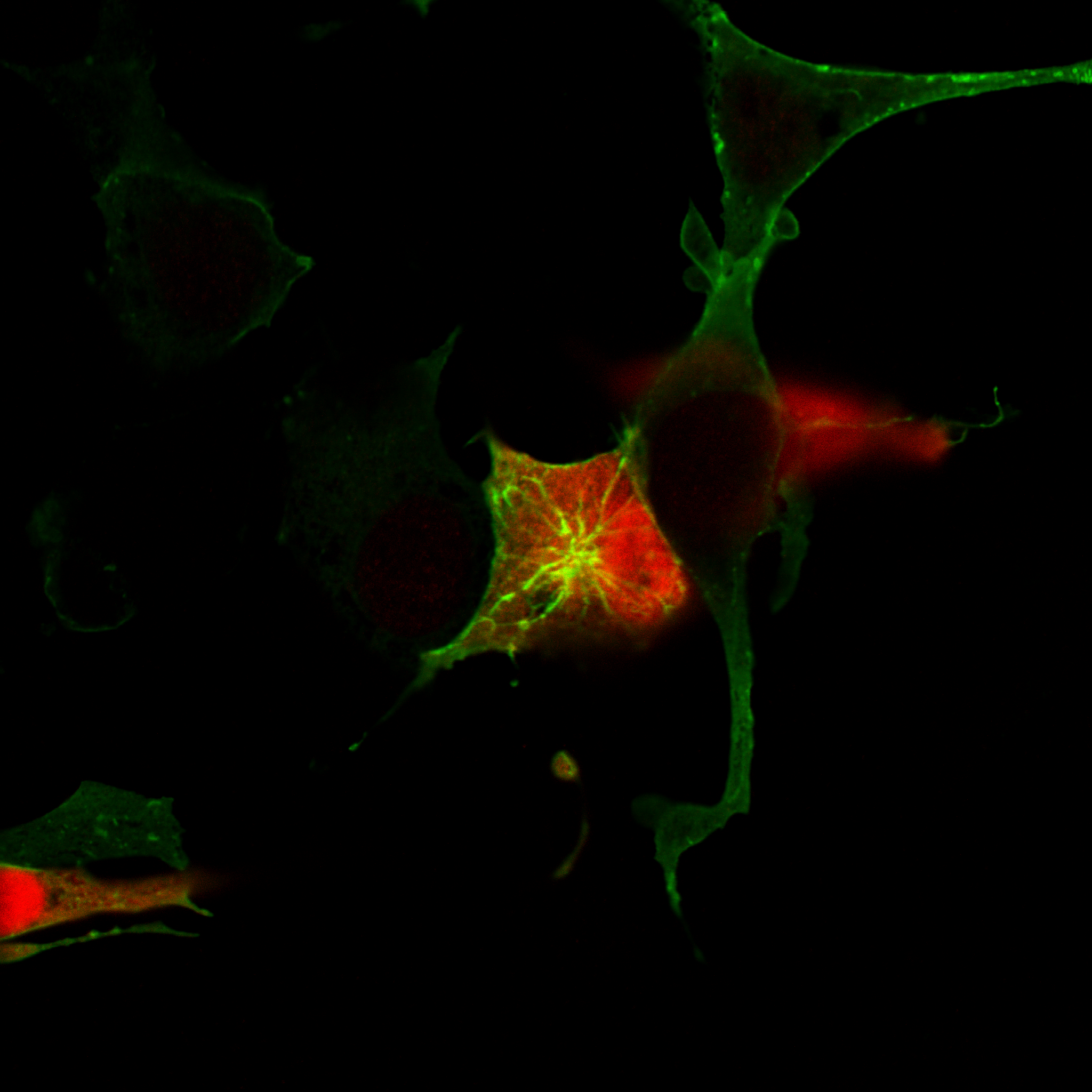

Supplement: Supplementary file 11 — Source data Fig. 8 [file 44318_2024_268_MOESM11_ESM.zip › Figure 8/8B/FCHO2 x Nedd4LC2triple.tif]

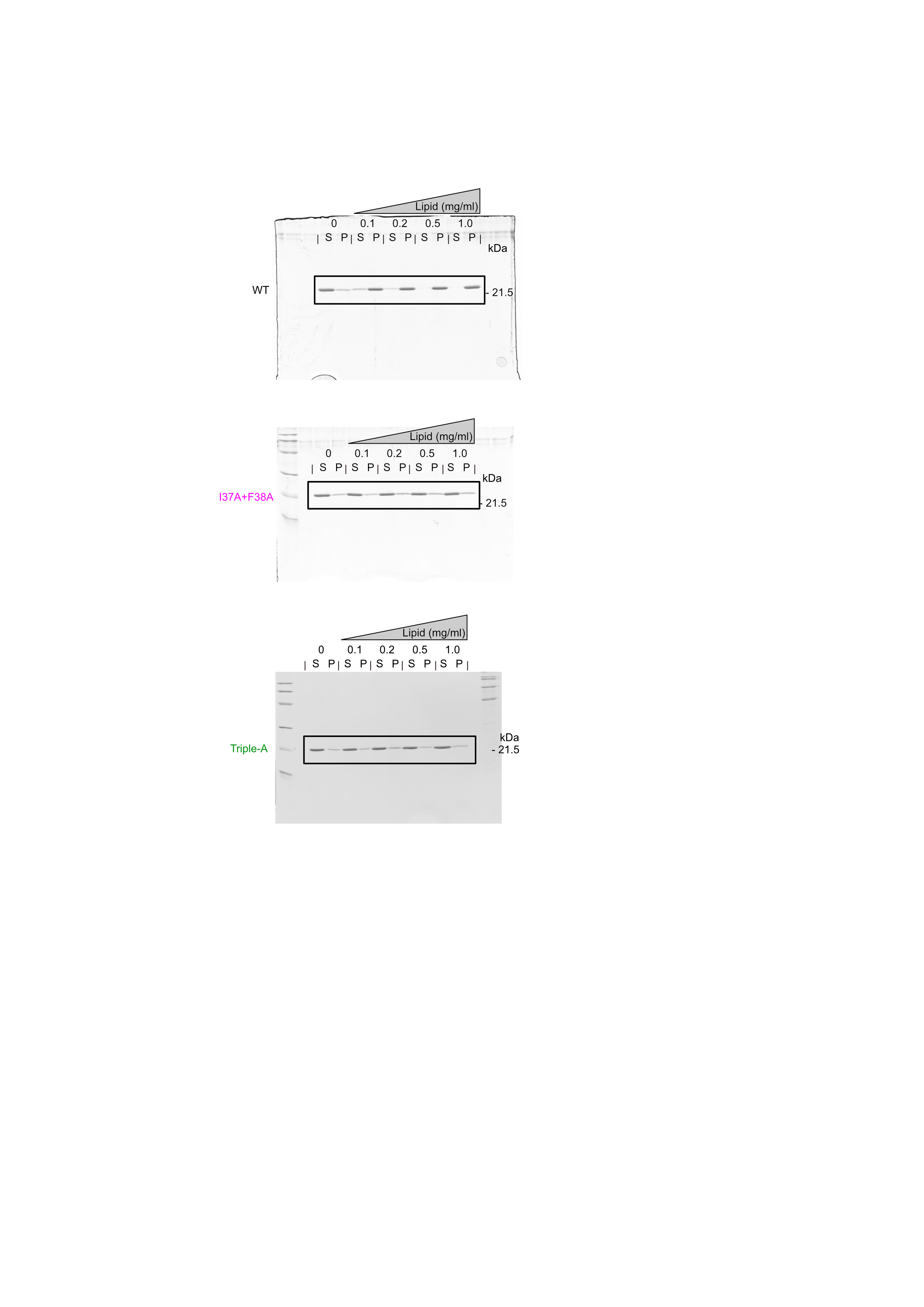

Supplement: Supplementary file 11 — Source data Fig. 8 [file 44318_2024_268_MOESM11_ESM.zip › Figure 8/8C/Fig8C.tiff]

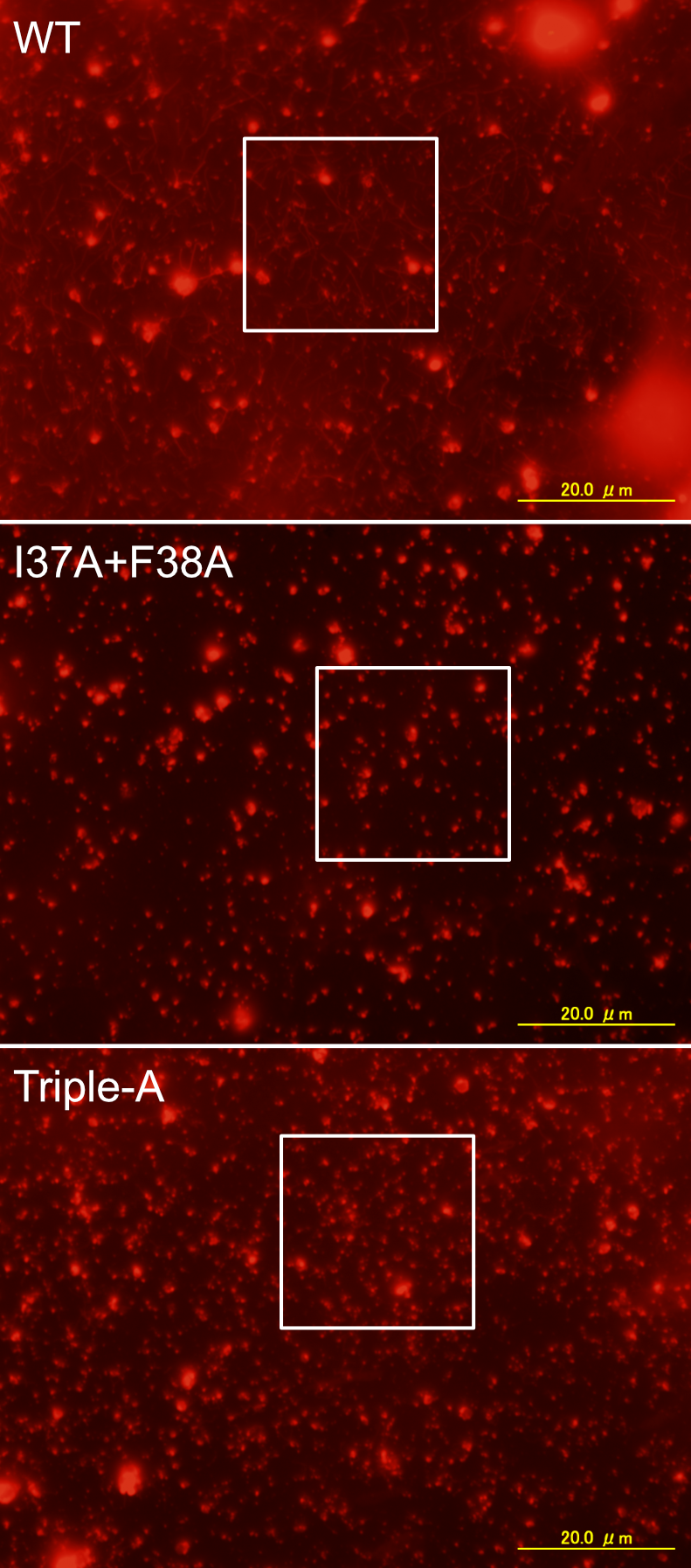

Supplement: Supplementary file 11 — Source data Fig. 8 [file 44318_2024_268_MOESM11_ESM.zip › Figure 8/8D/Fig8D.tiff]

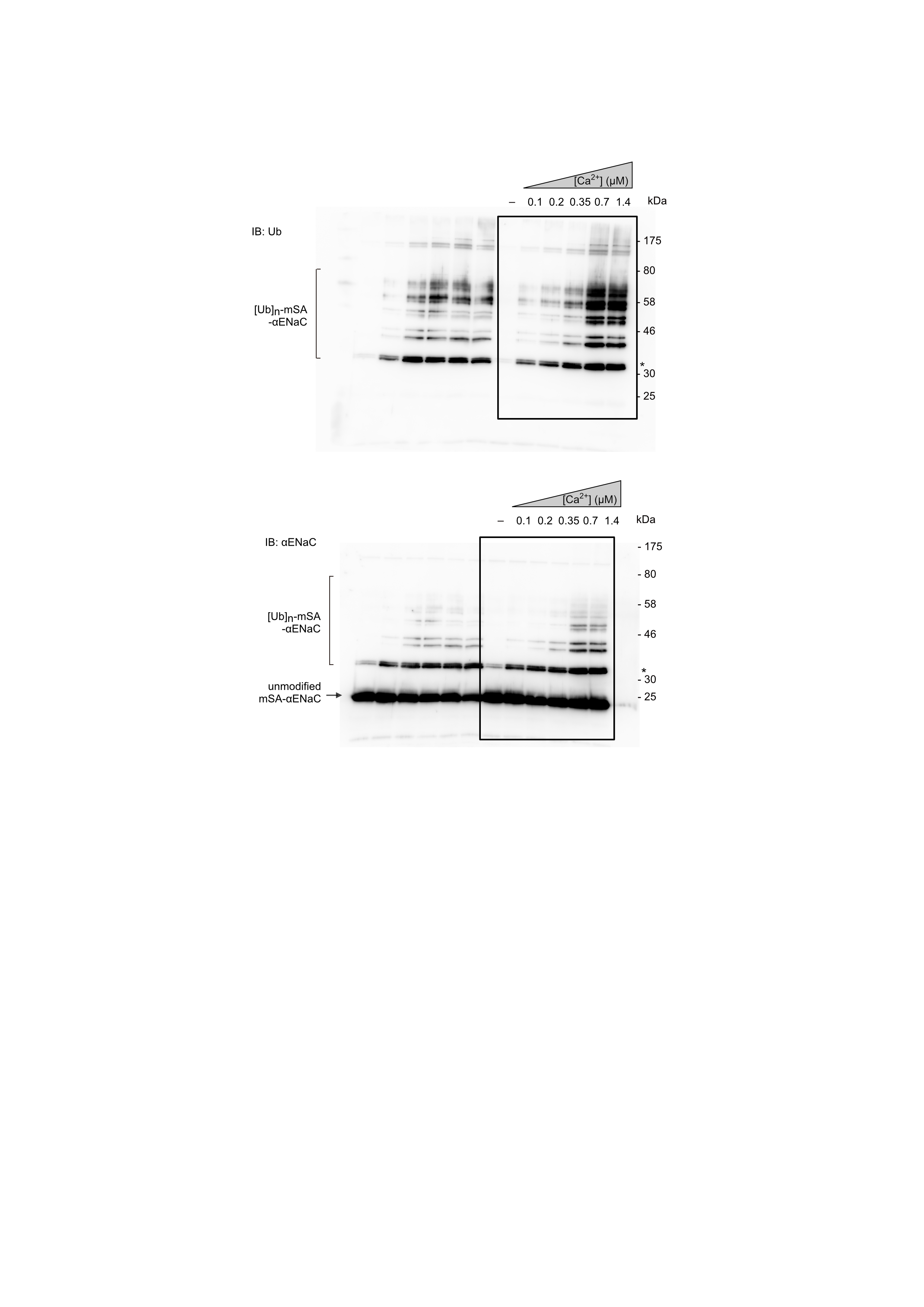

Supplement: Supplementary file 12 — Source data Fig. 9 [file 44318_2024_268_MOESM12_ESM.zip › EMBOJ-2023-114687R2_SourceDataForFig9/9C/Fig9C.tiff]

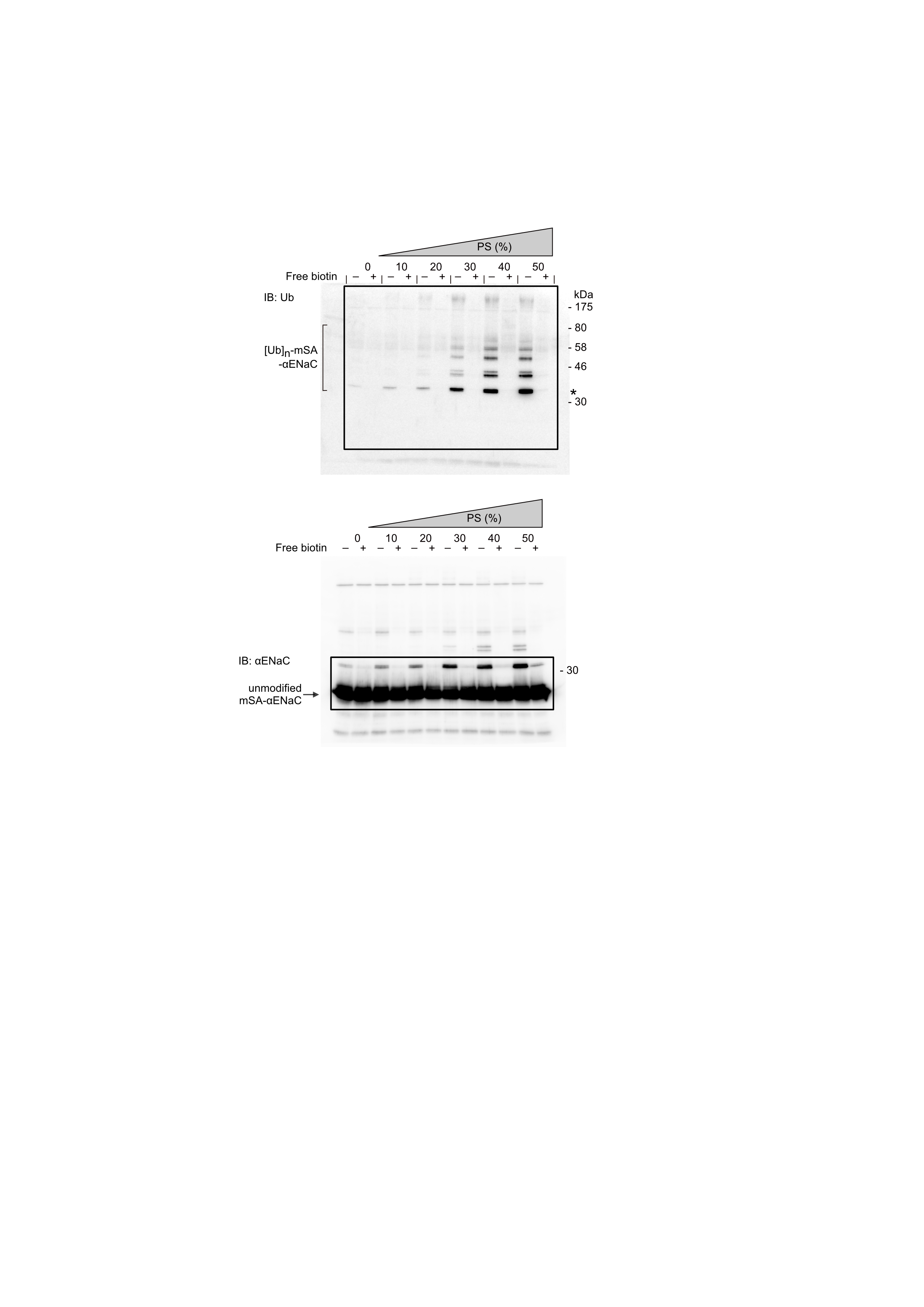

Supplement: Supplementary file 12 — Source data Fig. 9 [file 44318_2024_268_MOESM12_ESM.zip › EMBOJ-2023-114687R2_SourceDataForFig9/9D/Fig9D.tiff]

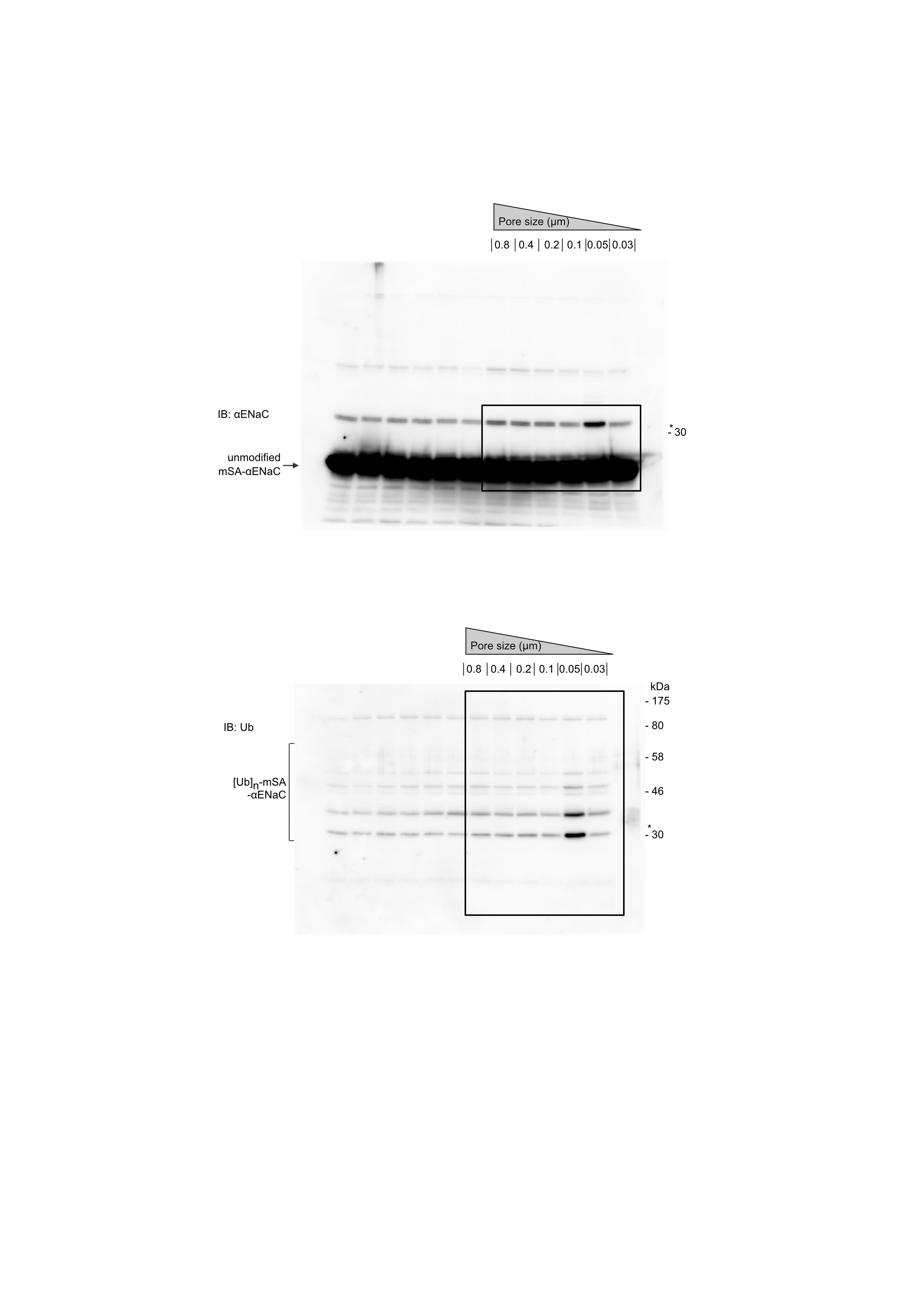

Supplement: Supplementary file 12 — Source data Fig. 9 [file 44318_2024_268_MOESM12_ESM.zip › EMBOJ-2023-114687R2_SourceDataForFig9/9E/Fig9E.tiff]

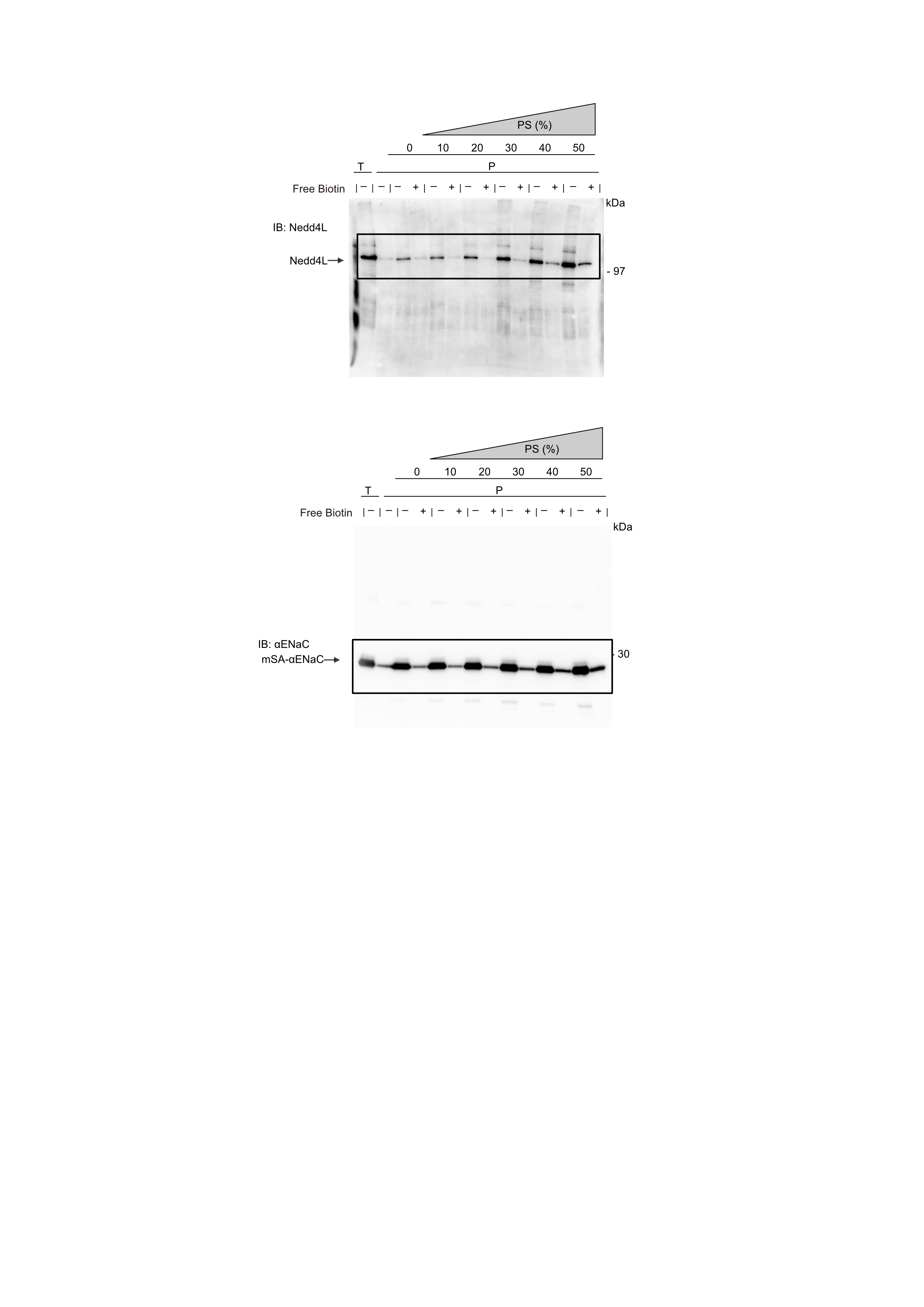

Supplement: Supplementary file 12 — Source data Fig. 9 [file 44318_2024_268_MOESM12_ESM.zip › EMBOJ-2023-114687R2_SourceDataForFig9/9F/Fig9F.tiff]

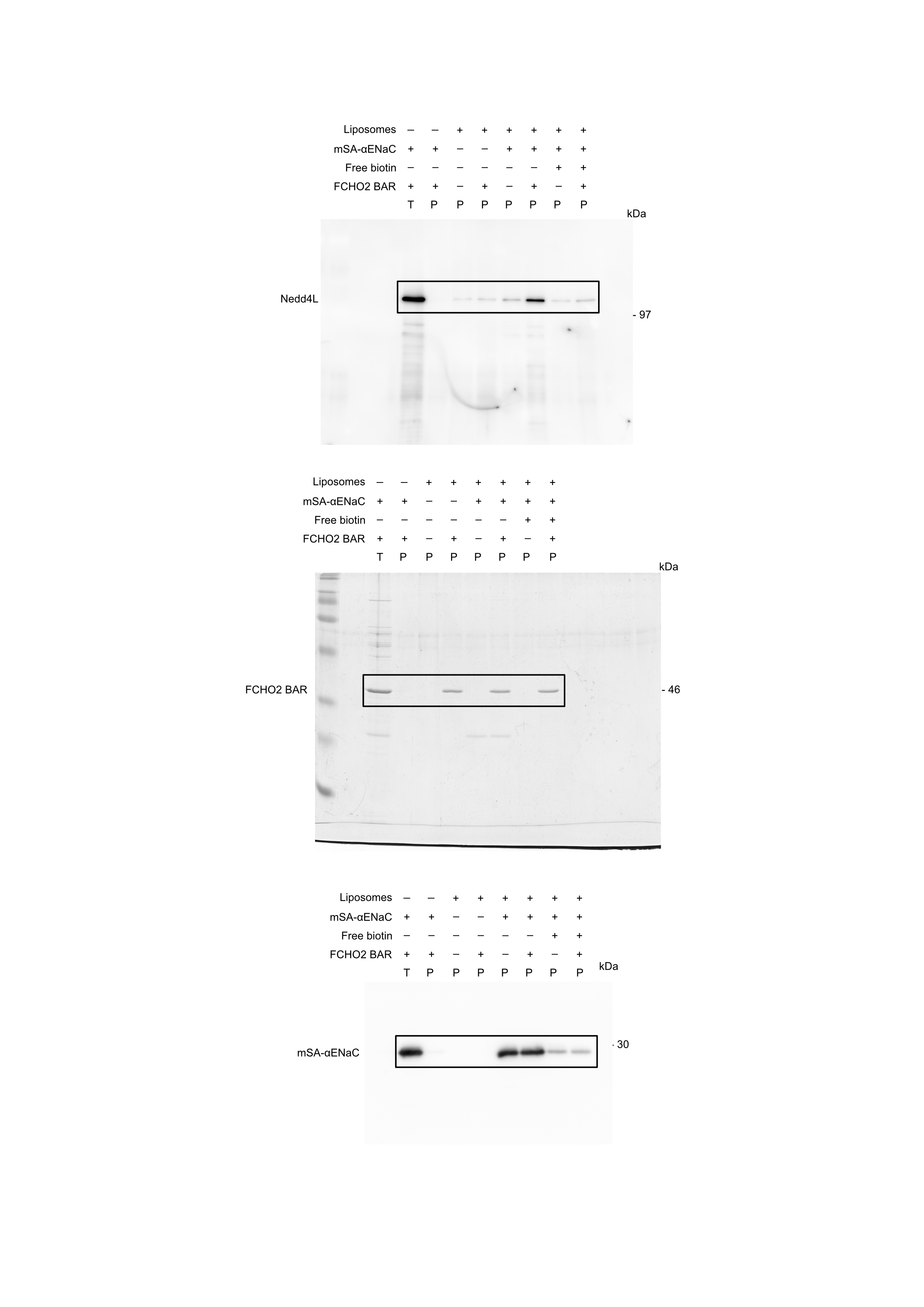

Supplement: Supplementary file 13 — Source data Fig. 10 [file 44318_2024_268_MOESM13_ESM.zip › Figure 10/10A/Fig10A.tiff]

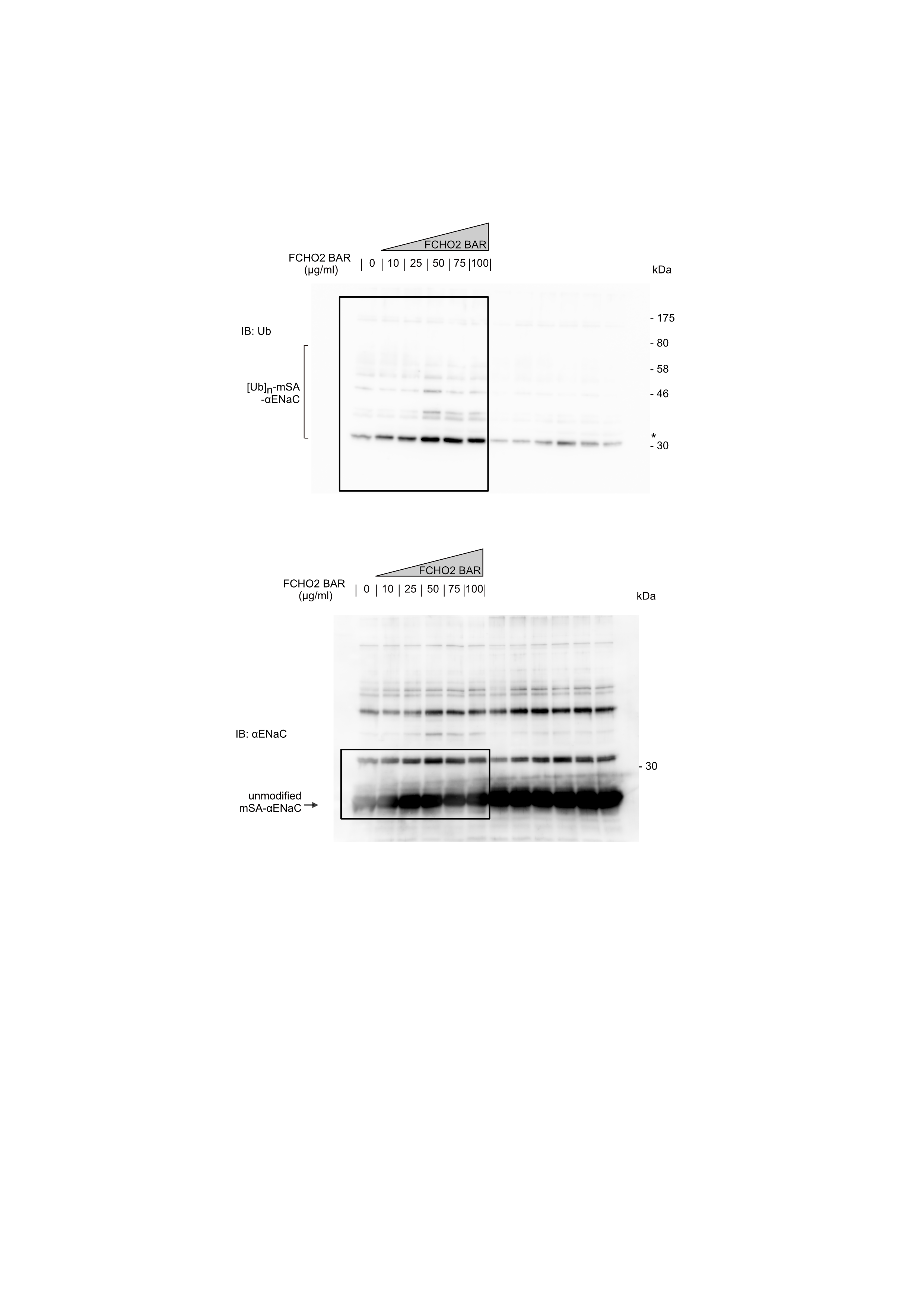

Supplement: Supplementary file 13 — Source data Fig. 10 [file 44318_2024_268_MOESM13_ESM.zip › Figure 10/10B/Fig10B.tiff]

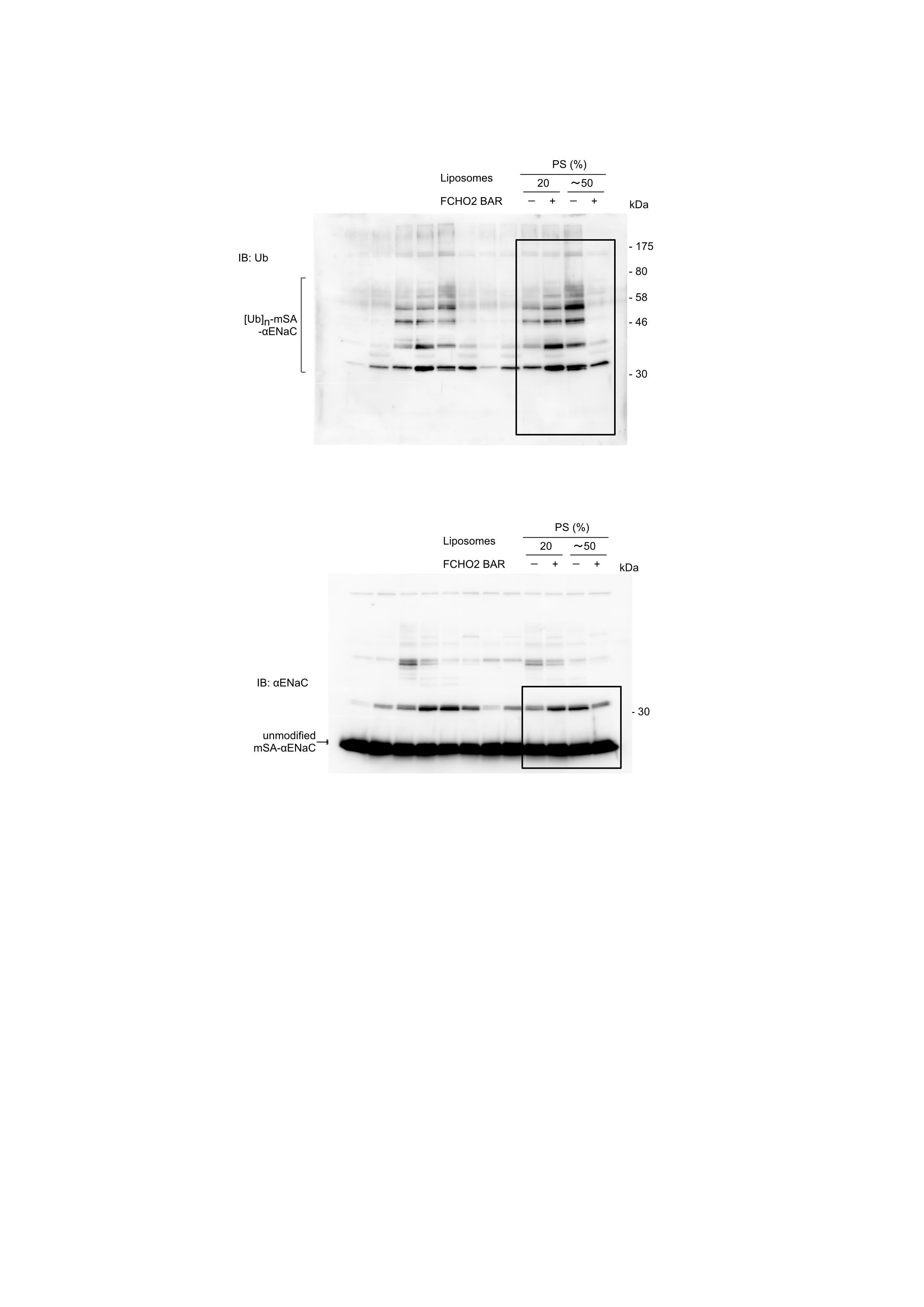

Supplement: Supplementary file 13 — Source data Fig. 10 [file 44318_2024_268_MOESM13_ESM.zip › Figure 10/10C/Fig10C.tiff]

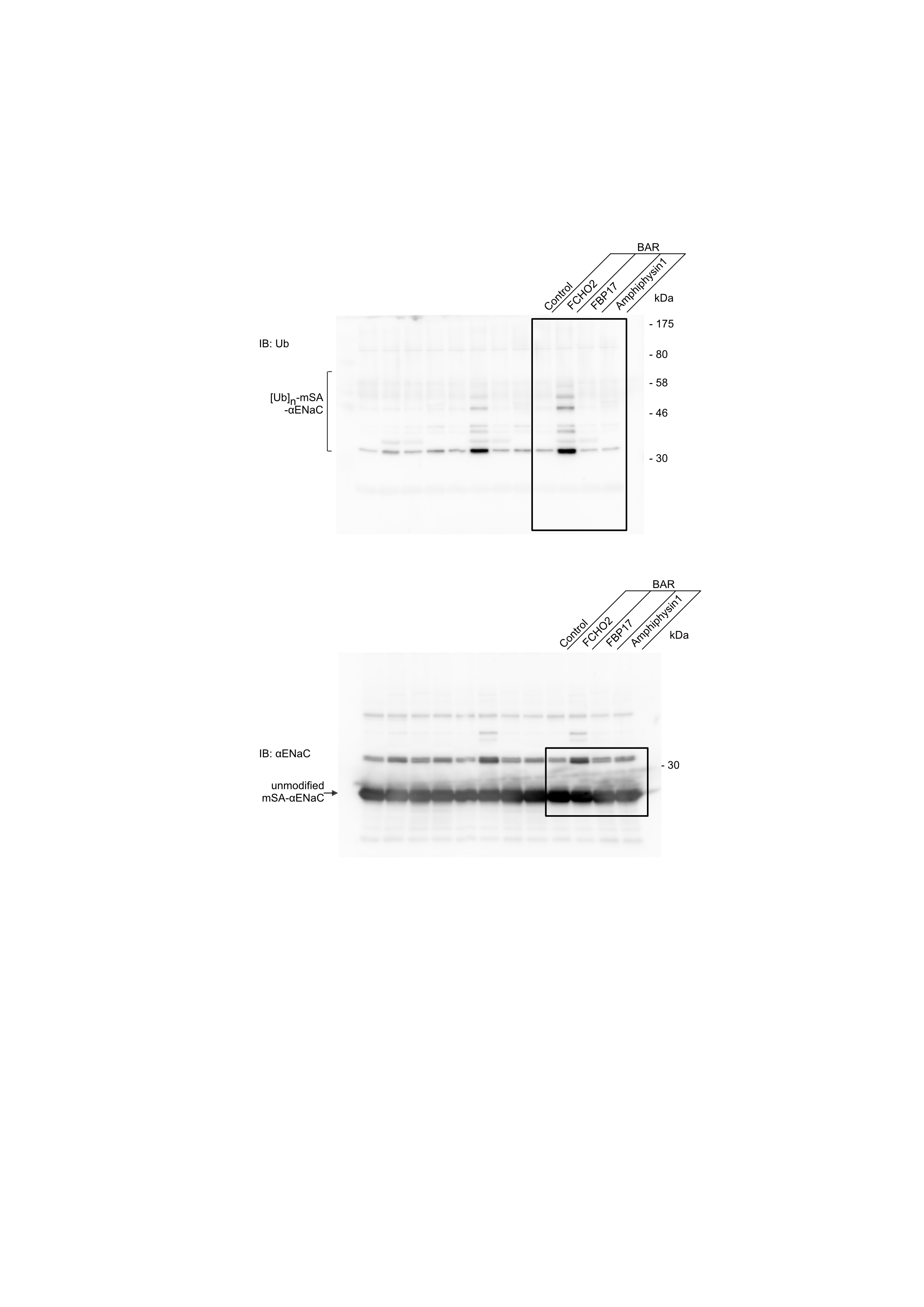

Supplement: Supplementary file 13 — Source data Fig. 10 [file 44318_2024_268_MOESM13_ESM.zip › Figure 10/10D/Fig10D.tiff]

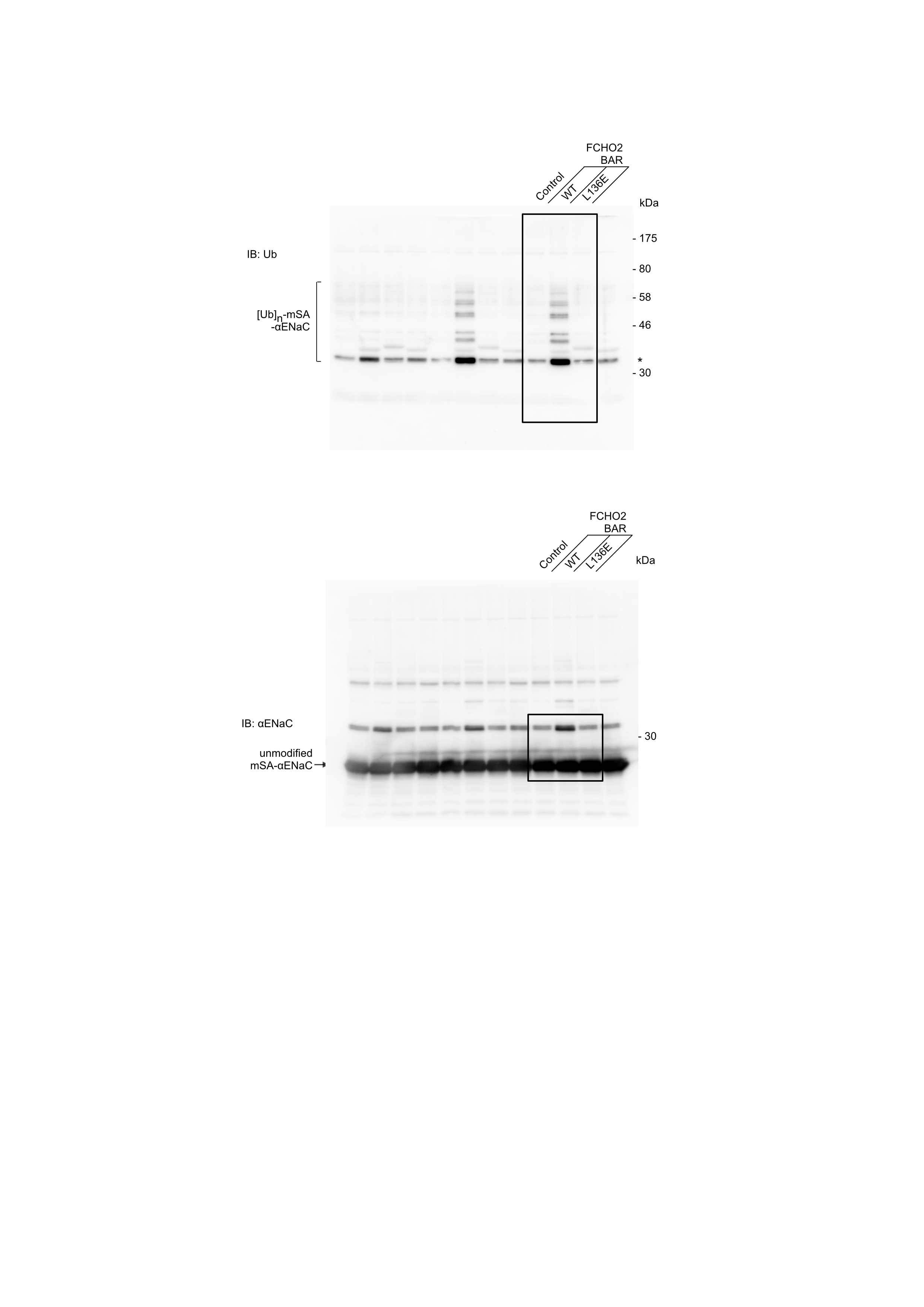

Supplement: Supplementary file 13 — Source data Fig. 10 [file 44318_2024_268_MOESM13_ESM.zip › Figure 10/10E/Fig10E.tiff]

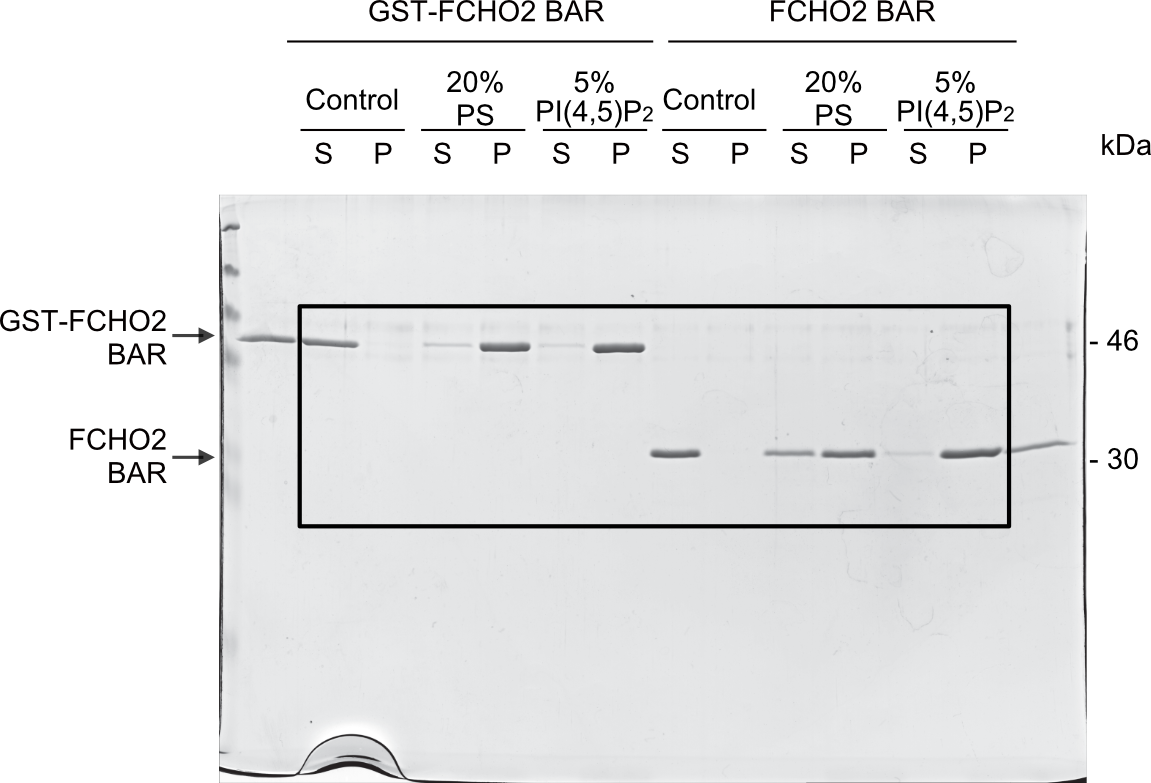

Supplement: Supplementary file 14 — Source data Fig. 11 [file 44318_2024_268_MOESM14_ESM.zip › Figure 11/11A/Fig11A.tiff]

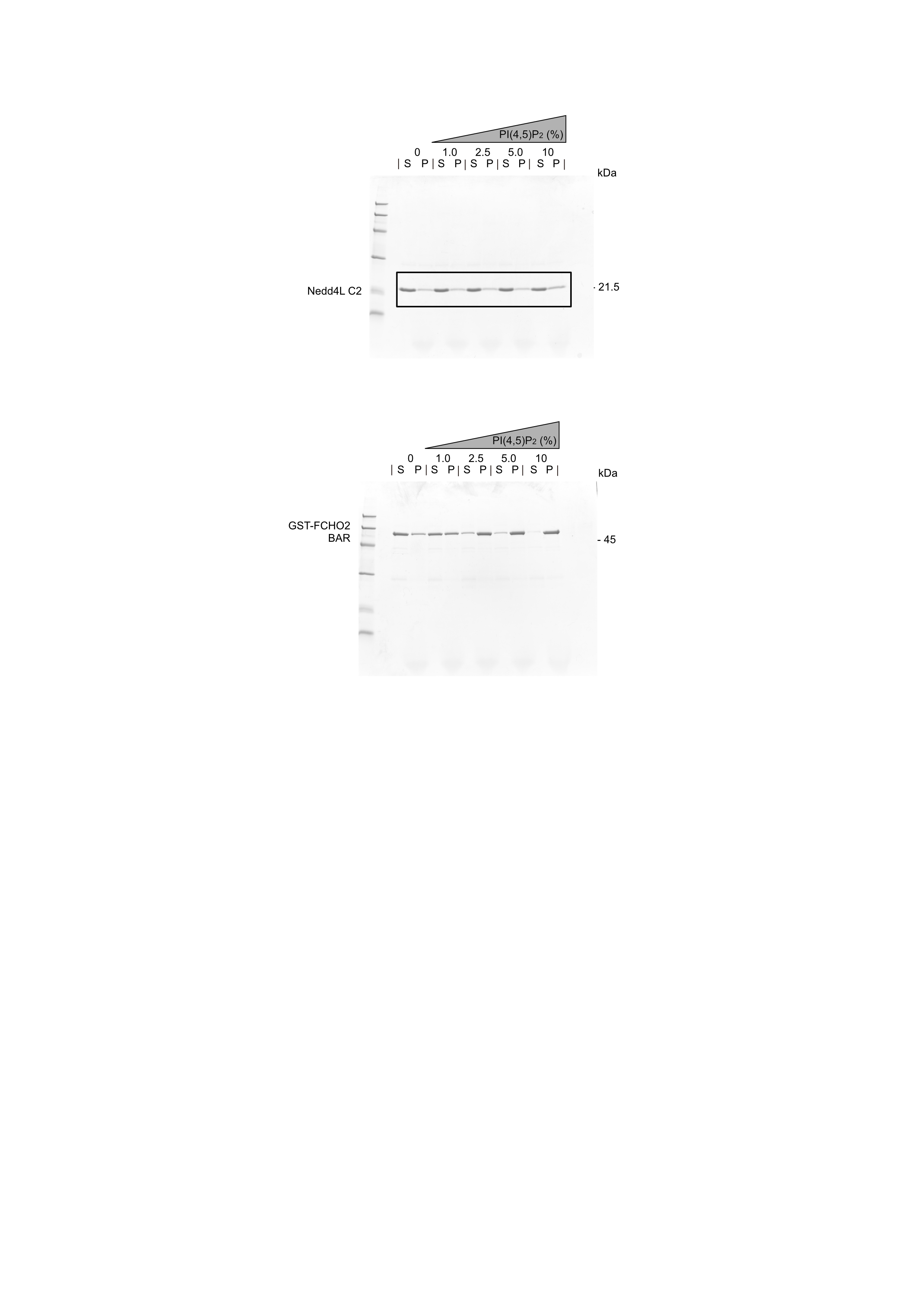

Supplement: Supplementary file 14 — Source data Fig. 11 [file 44318_2024_268_MOESM14_ESM.zip › Figure 11/11B/Fig11B.tiff]

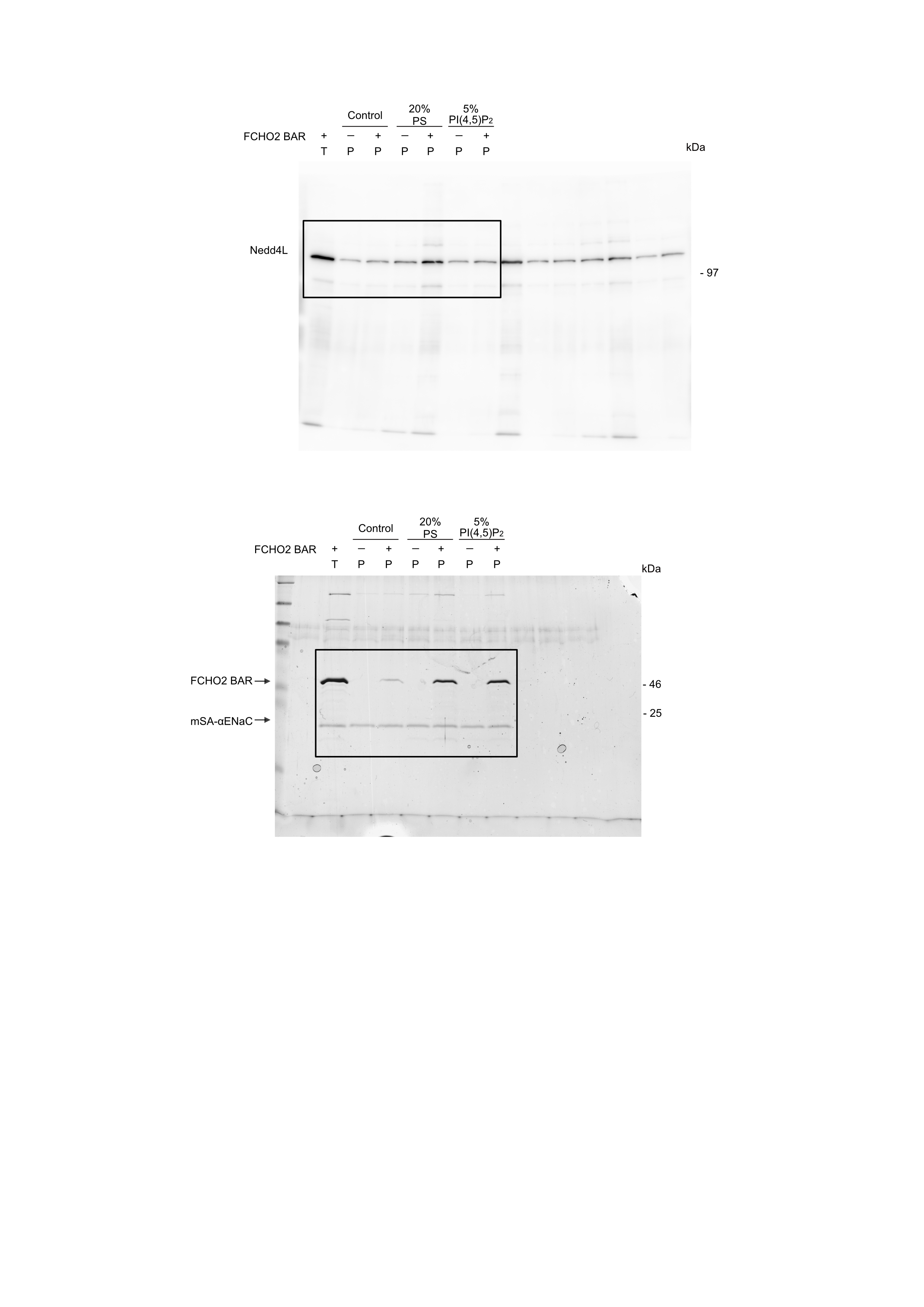

Supplement: Supplementary file 14 — Source data Fig. 11 [file 44318_2024_268_MOESM14_ESM.zip › Figure 11/11C/Fig11C.tiff]

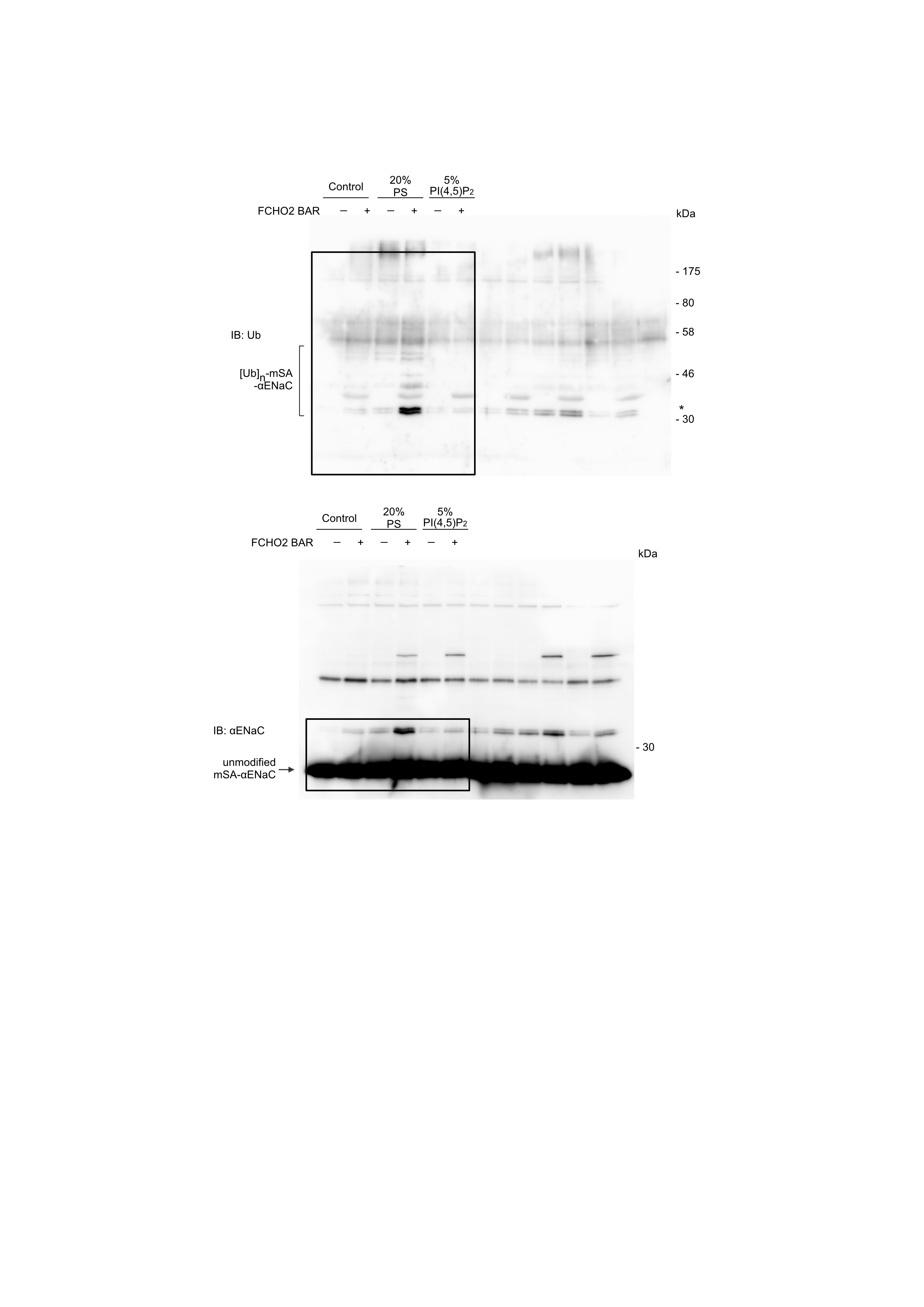

Supplement: Supplementary file 14 — Source data Fig. 11 [file 44318_2024_268_MOESM14_ESM.zip › Figure 11/11D/Fig11D.tiff]

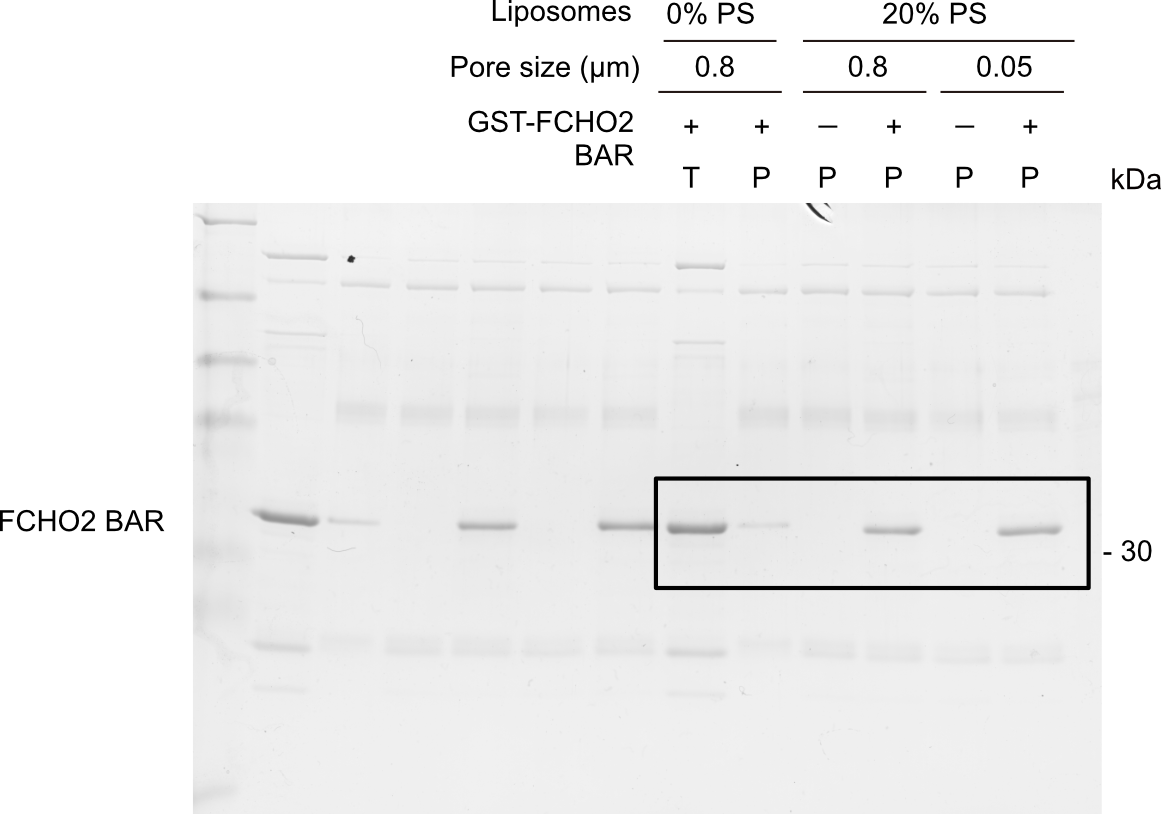

Supplement: Supplementary file 15 — Source data Fig. 12 [file 44318_2024_268_MOESM15_ESM.zip › Figure 12/12A/Fig12A.tiff]

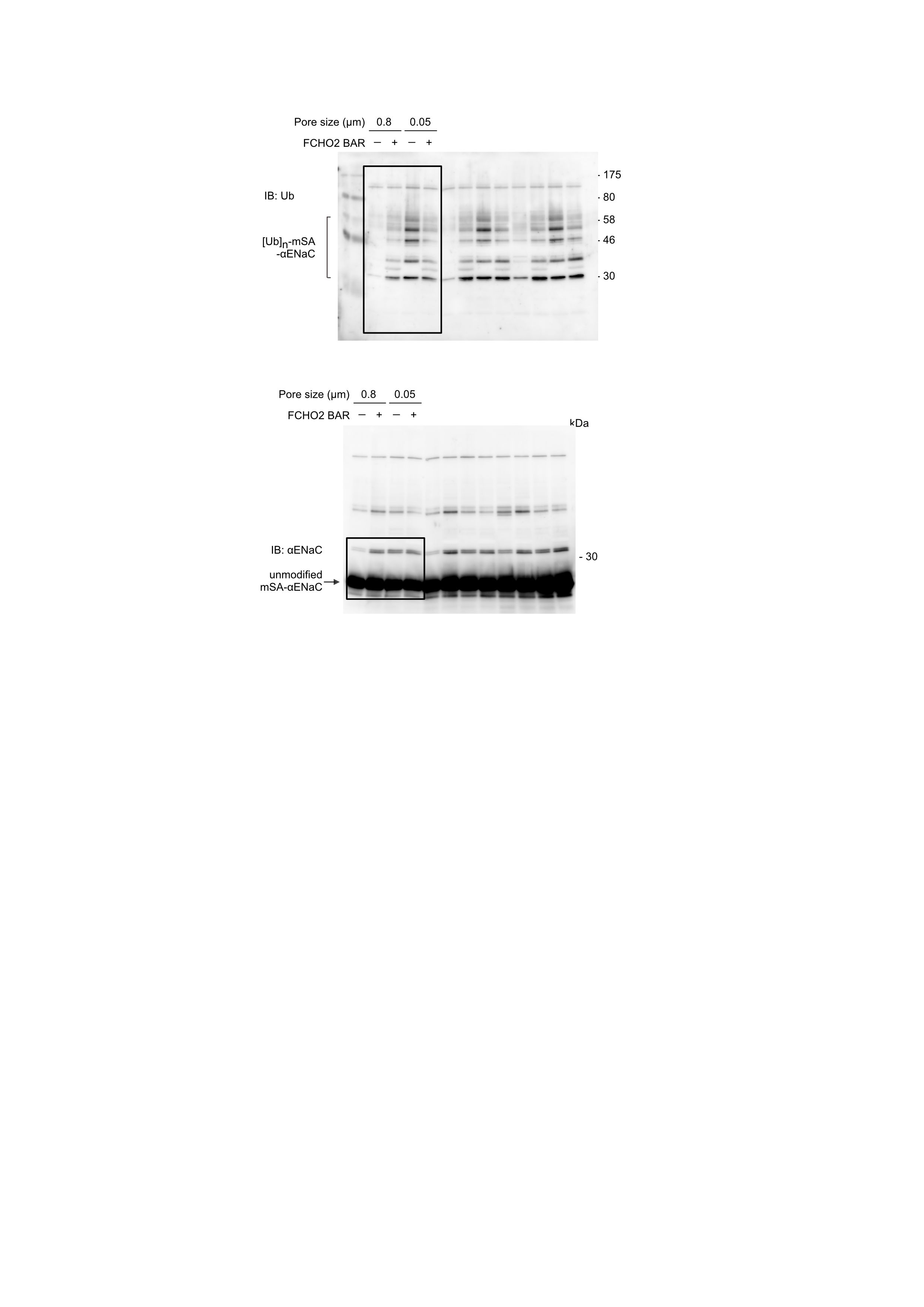

Supplement: Supplementary file 15 — Source data Fig. 12 [file 44318_2024_268_MOESM15_ESM.zip › Figure 12/12B/Fig12B.tiff]

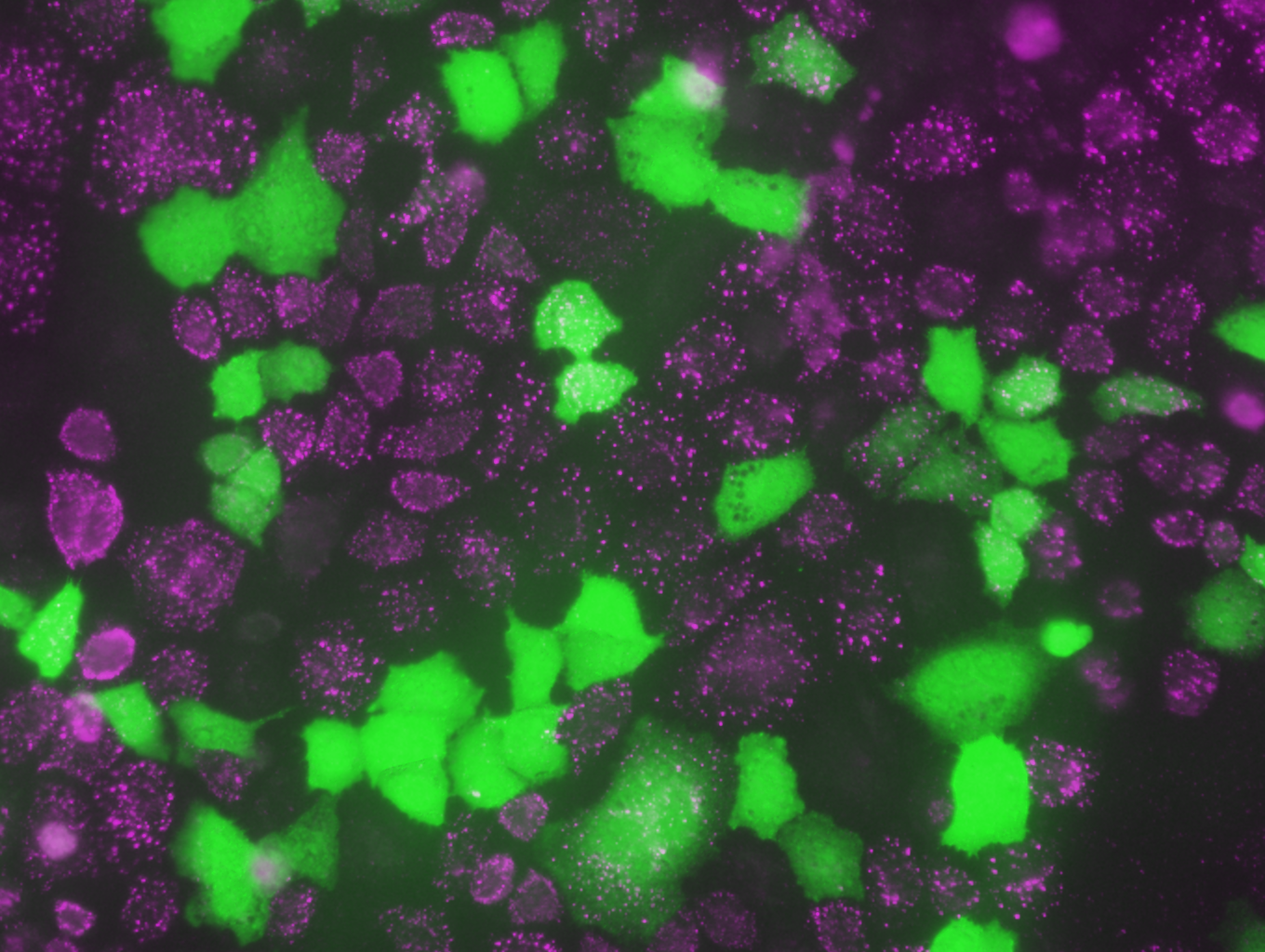

Supplement: Supplementary file 16 — Figure EV and Appendix Source Data [file 44318_2024_268_MOESM16_ESM.zip › Extended View and Appendix Source Data/Appendix Figure S1/GFP x siControl.tif]

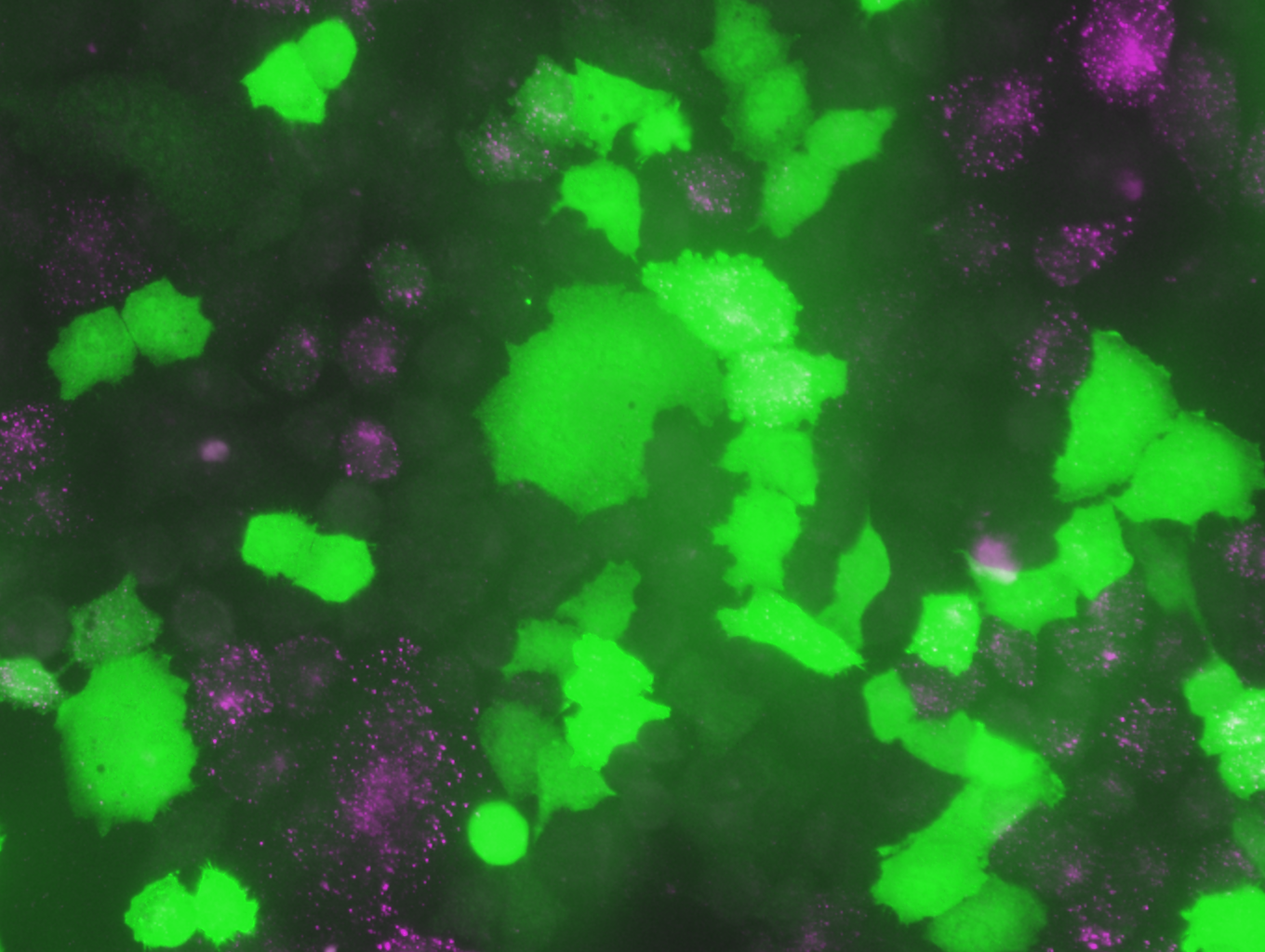

Supplement: Supplementary file 16 — Figure EV and Appendix Source Data [file 44318_2024_268_MOESM16_ESM.zip › Extended View and Appendix Source Data/Appendix Figure S1/GFP x siFCHO2.tif]

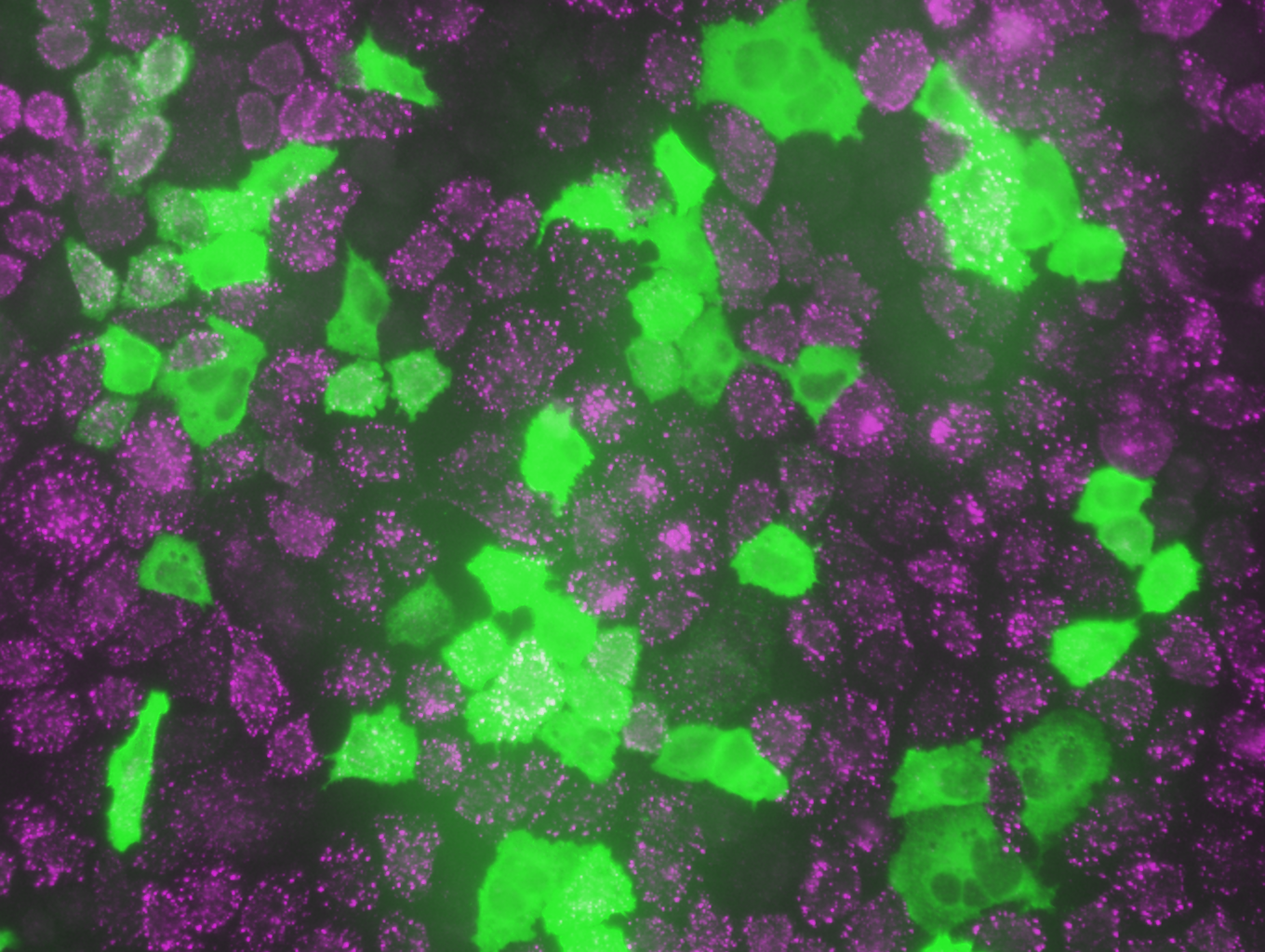

Supplement: Supplementary file 16 — Figure EV and Appendix Source Data [file 44318_2024_268_MOESM16_ESM.zip › Extended View and Appendix Source Data/Appendix Figure S1/GFPsrFCHO2 x siControl.tif]

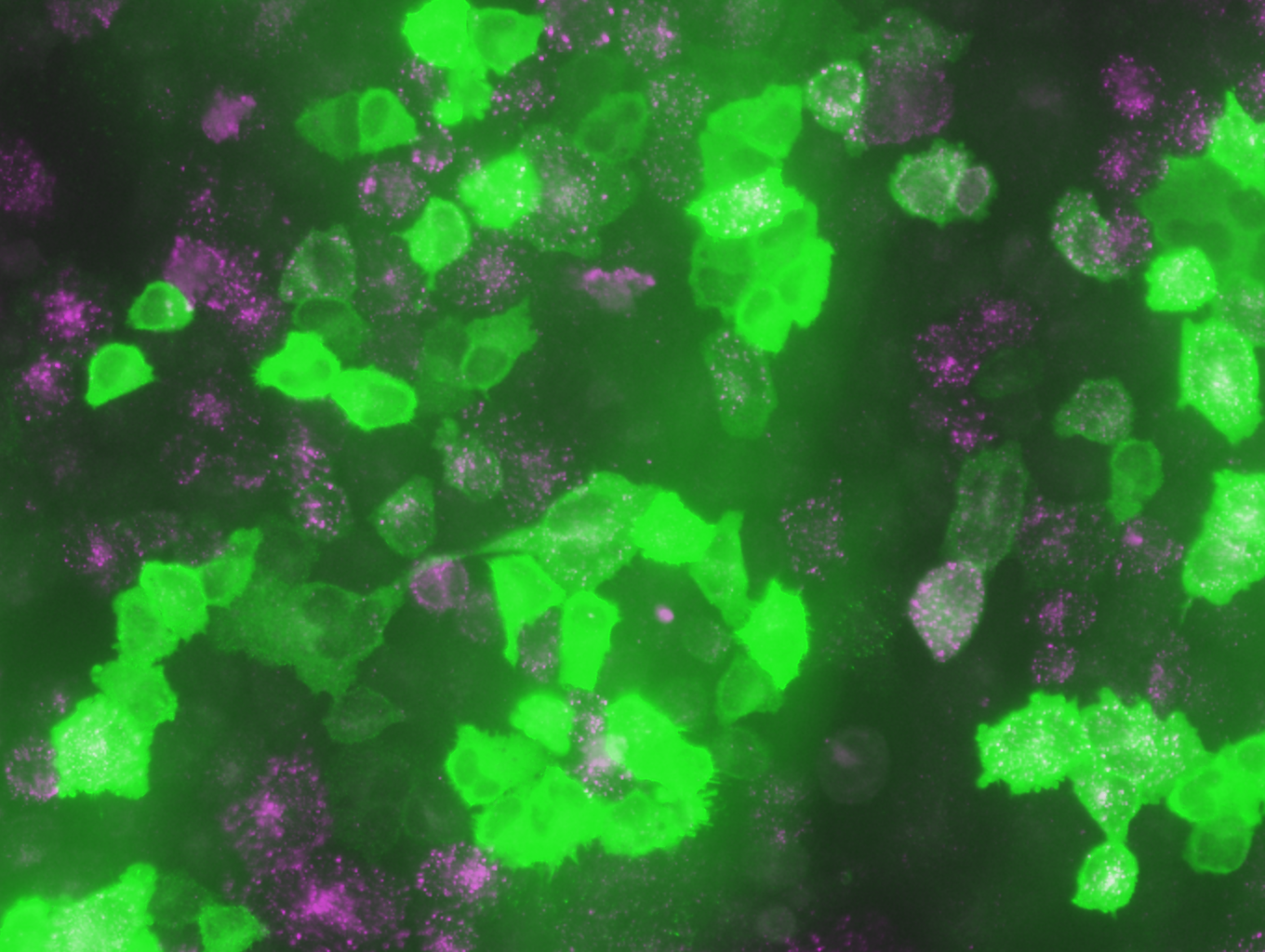

Supplement: Supplementary file 16 — Figure EV and Appendix Source Data [file 44318_2024_268_MOESM16_ESM.zip › Extended View and Appendix Source Data/Appendix Figure S1/GFPsrFCHO2 x siFCHO2.tif]

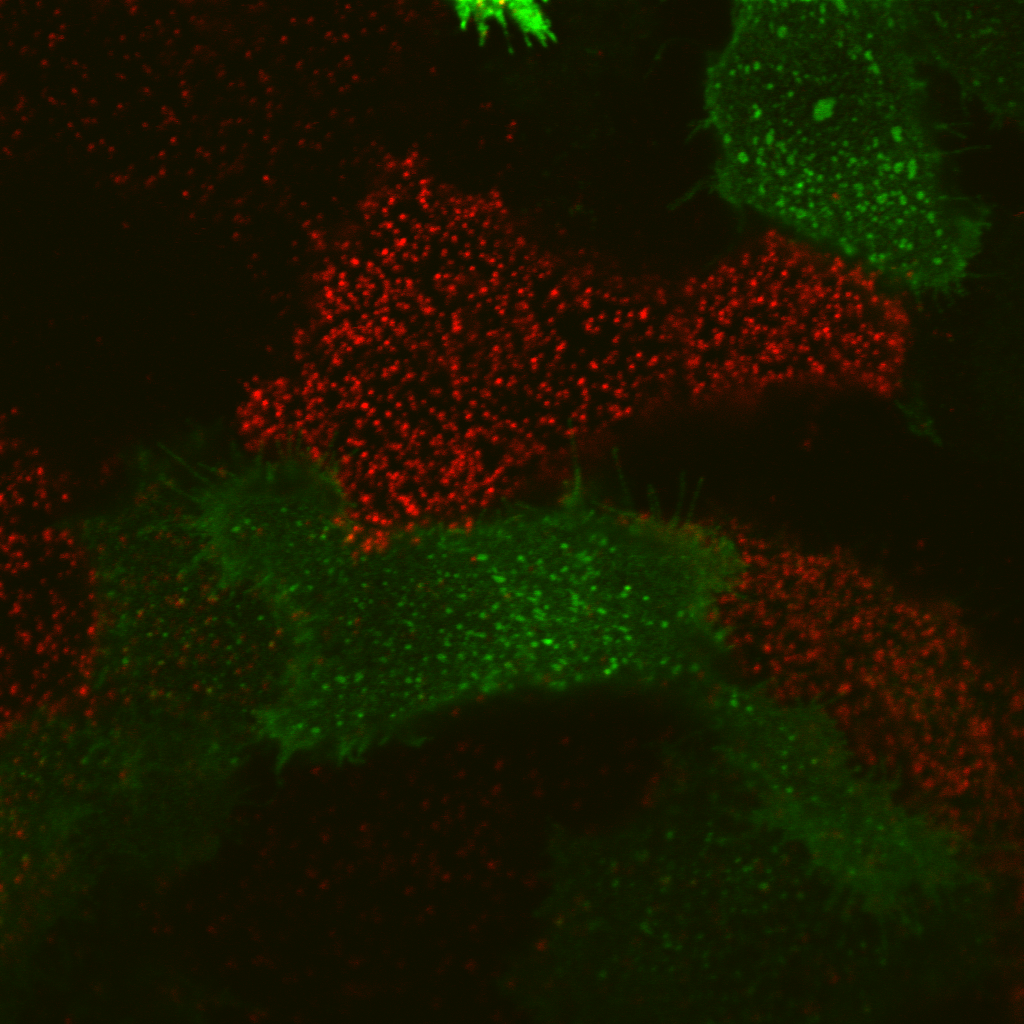

Supplement: Supplementary file 16 — Figure EV and Appendix Source Data [file 44318_2024_268_MOESM16_ESM.zip › Extended View and Appendix Source Data/Appendix Figure S2/S2A/Nedd4L x ENaC.tif]

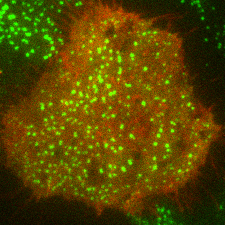

Supplement: Supplementary file 16 — Figure EV and Appendix Source Data [file 44318_2024_268_MOESM16_ESM.zip › Extended View and Appendix Source Data/Appendix Figure S2/S2B/FCHO2 x Nedd4L.tif]

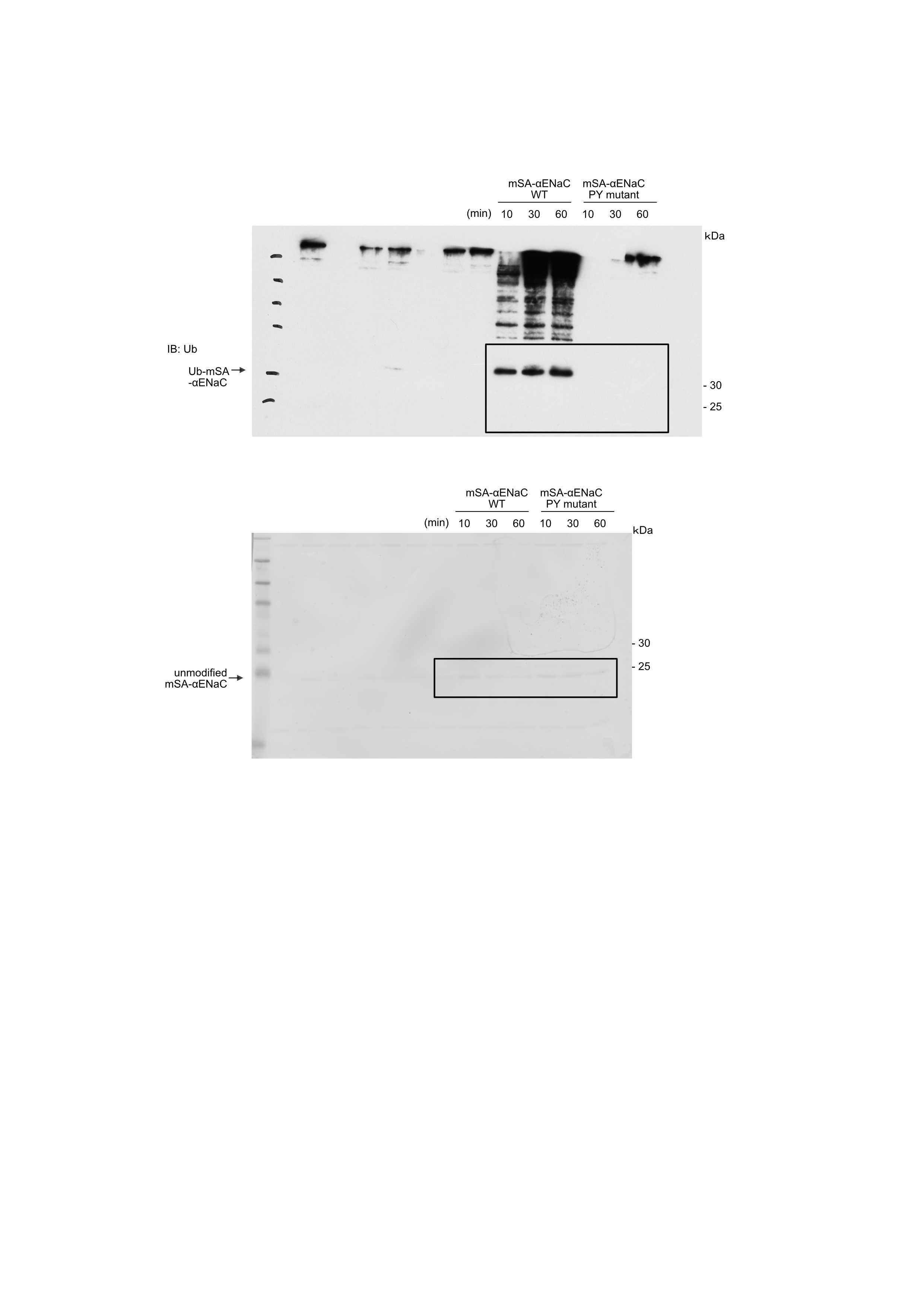

Supplement: Supplementary file 16 — Figure EV and Appendix Source Data [file 44318_2024_268_MOESM16_ESM.zip › Extended View and Appendix Source Data/Appendix Figure S3/FigS3.tiff]

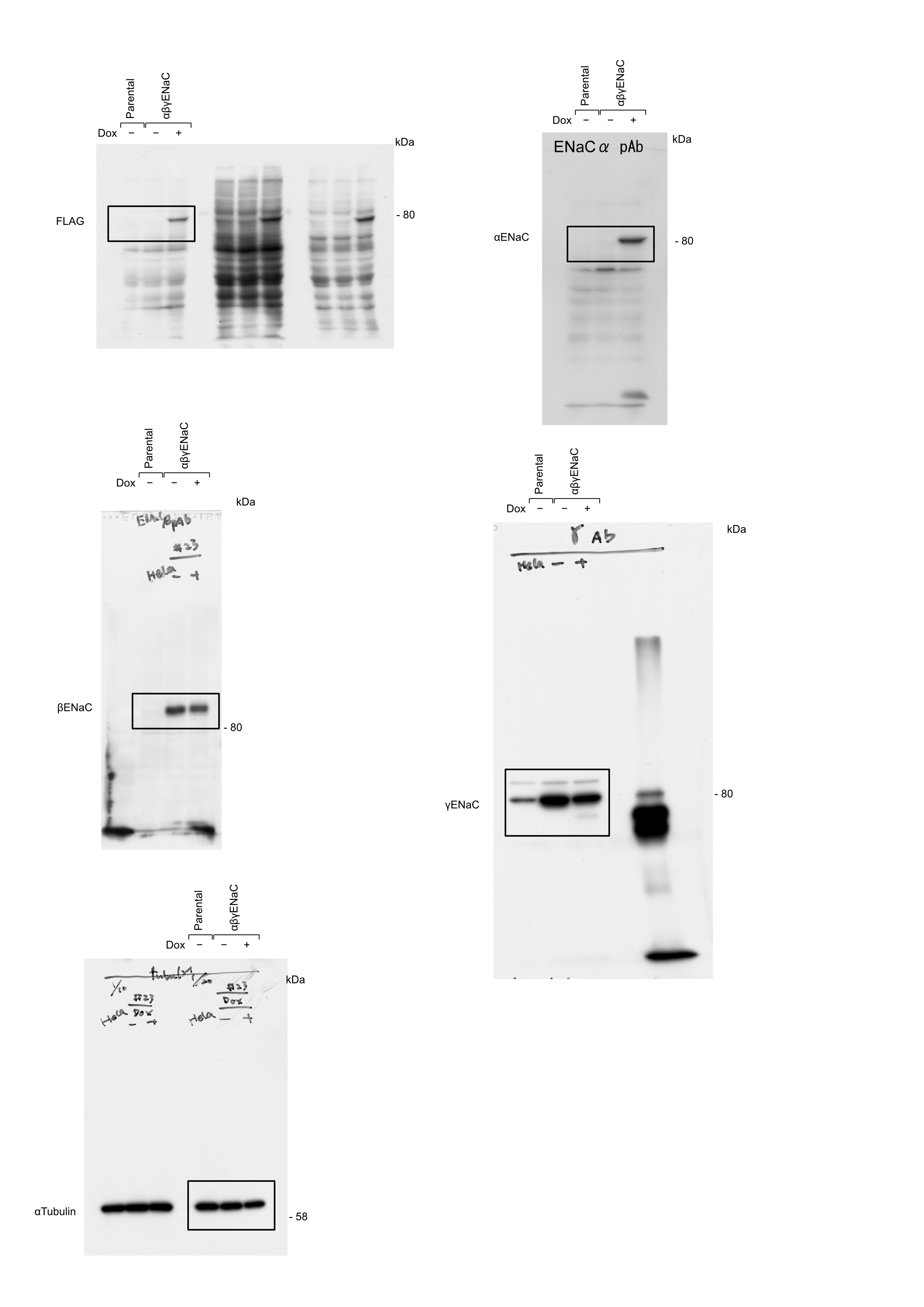

Supplement: Supplementary file 16 — Figure EV and Appendix Source Data [file 44318_2024_268_MOESM16_ESM.zip › Extended View and Appendix Source Data/Figure EV1/EV1A/FigEV1A.tiff]

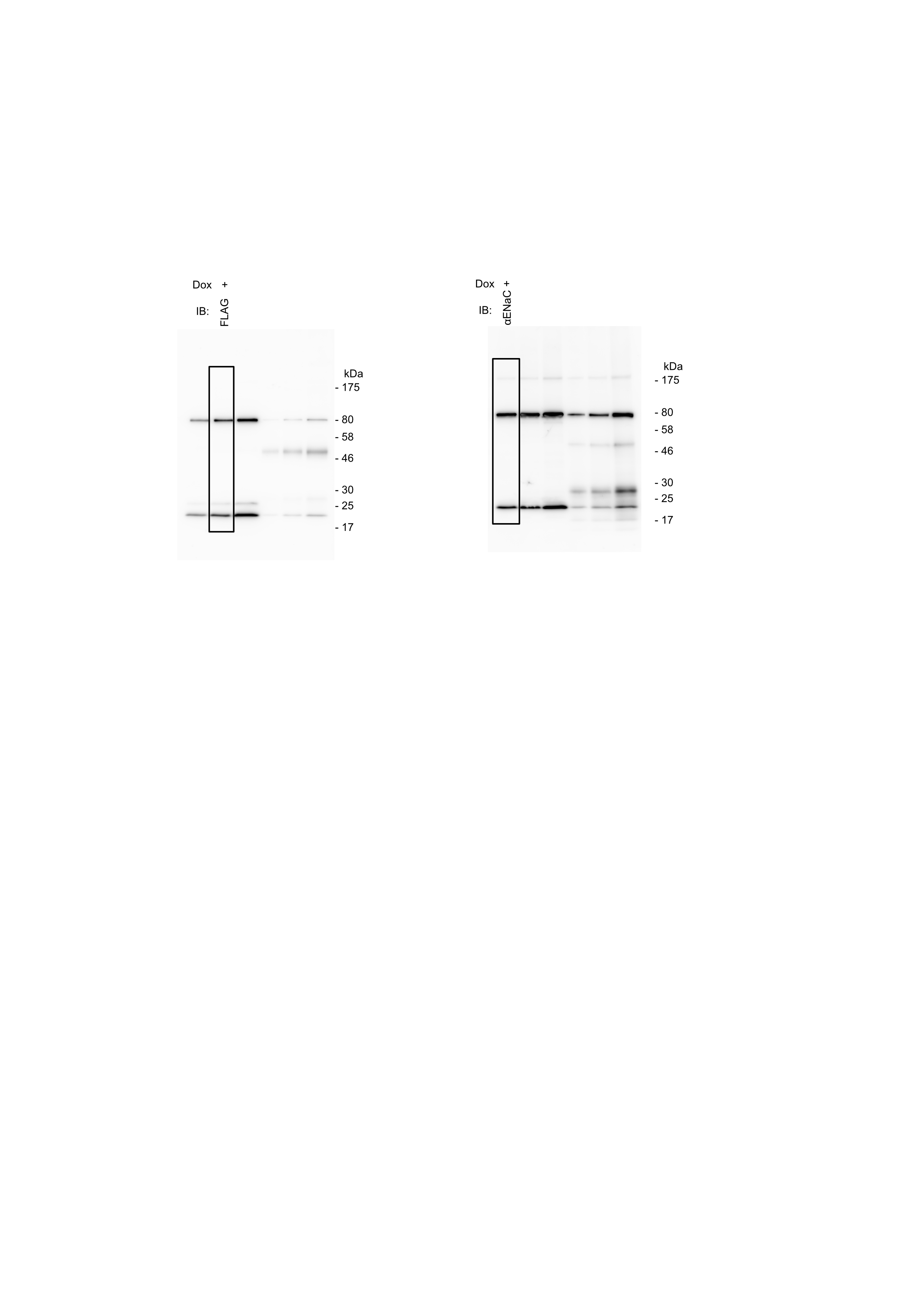

Supplement: Supplementary file 16 — Figure EV and Appendix Source Data [file 44318_2024_268_MOESM16_ESM.zip › Extended View and Appendix Source Data/Figure EV1/EV1B/FigEV1B.tiff]

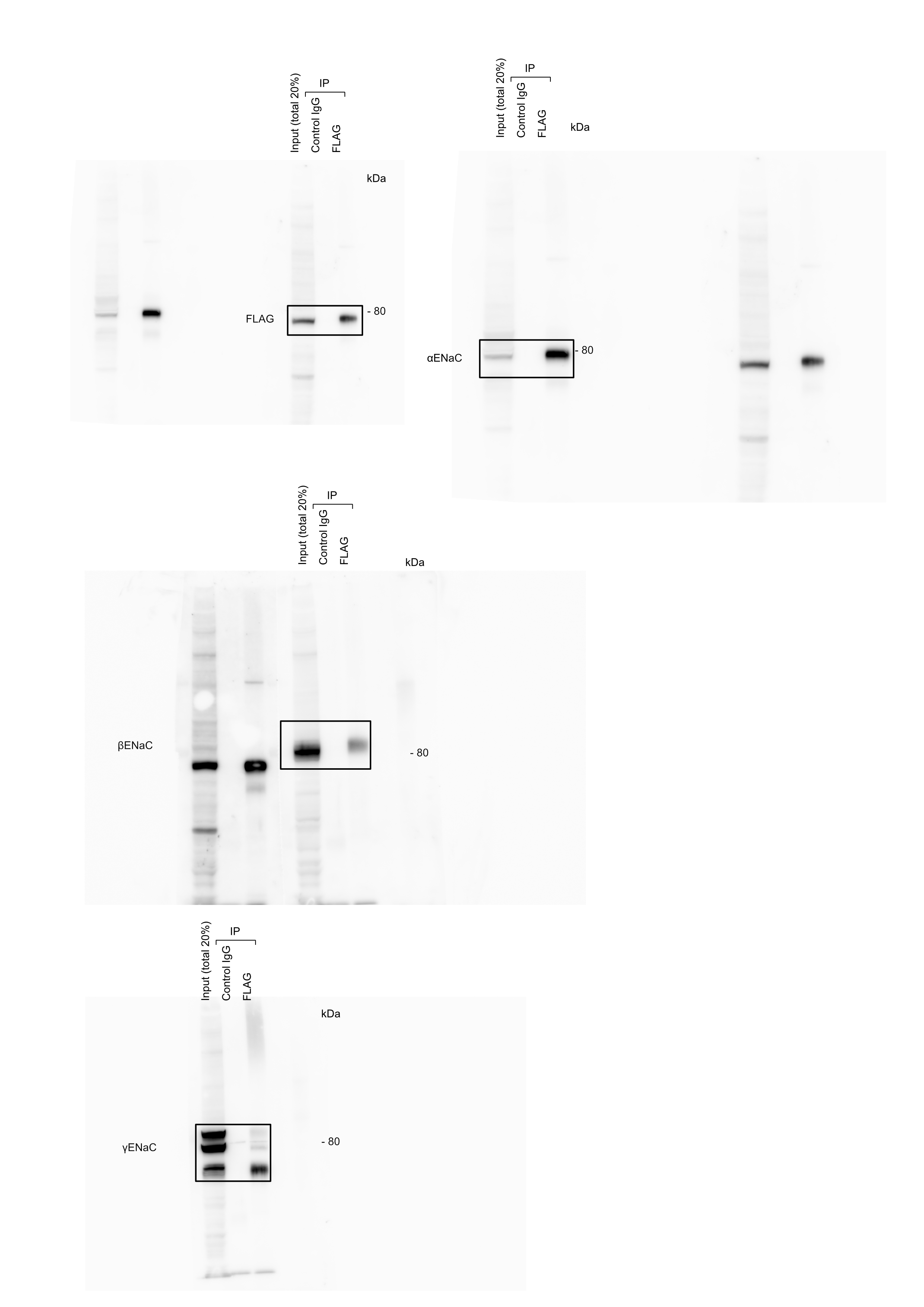

Supplement: Supplementary file 16 — Figure EV and Appendix Source Data [file 44318_2024_268_MOESM16_ESM.zip › Extended View and Appendix Source Data/Figure EV1/EV1C/FigEV1C.tiff]

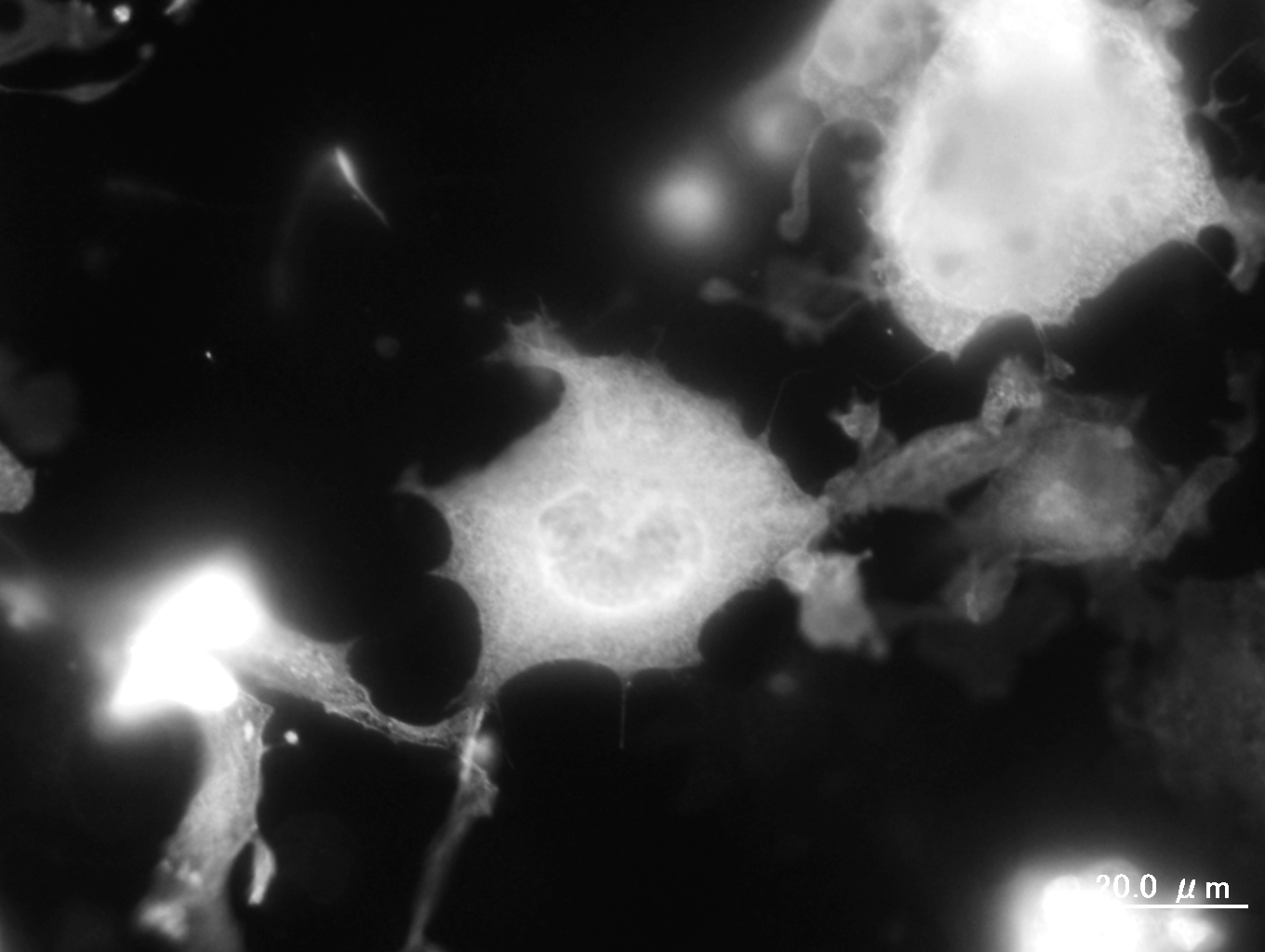

Supplement: Supplementary file 16 — Figure EV and Appendix Source Data [file 44318_2024_268_MOESM16_ESM.zip › Extended View and Appendix Source Data/Figure EV3/FCHO2K146ER152E.tif]

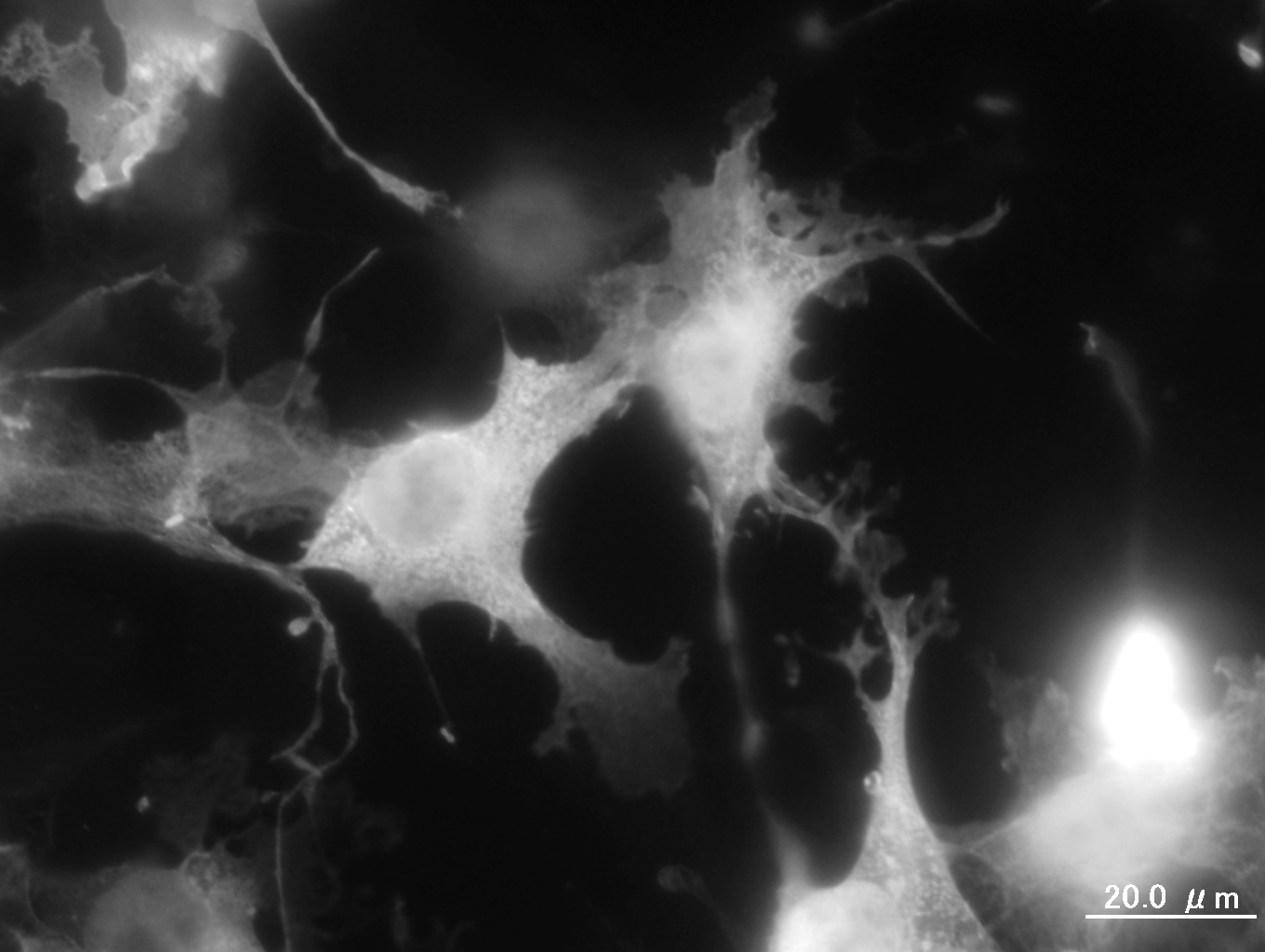

Supplement: Supplementary file 16 — Figure EV and Appendix Source Data [file 44318_2024_268_MOESM16_ESM.zip › Extended View and Appendix Source Data/Figure EV3/FCHO2L136E.tif]

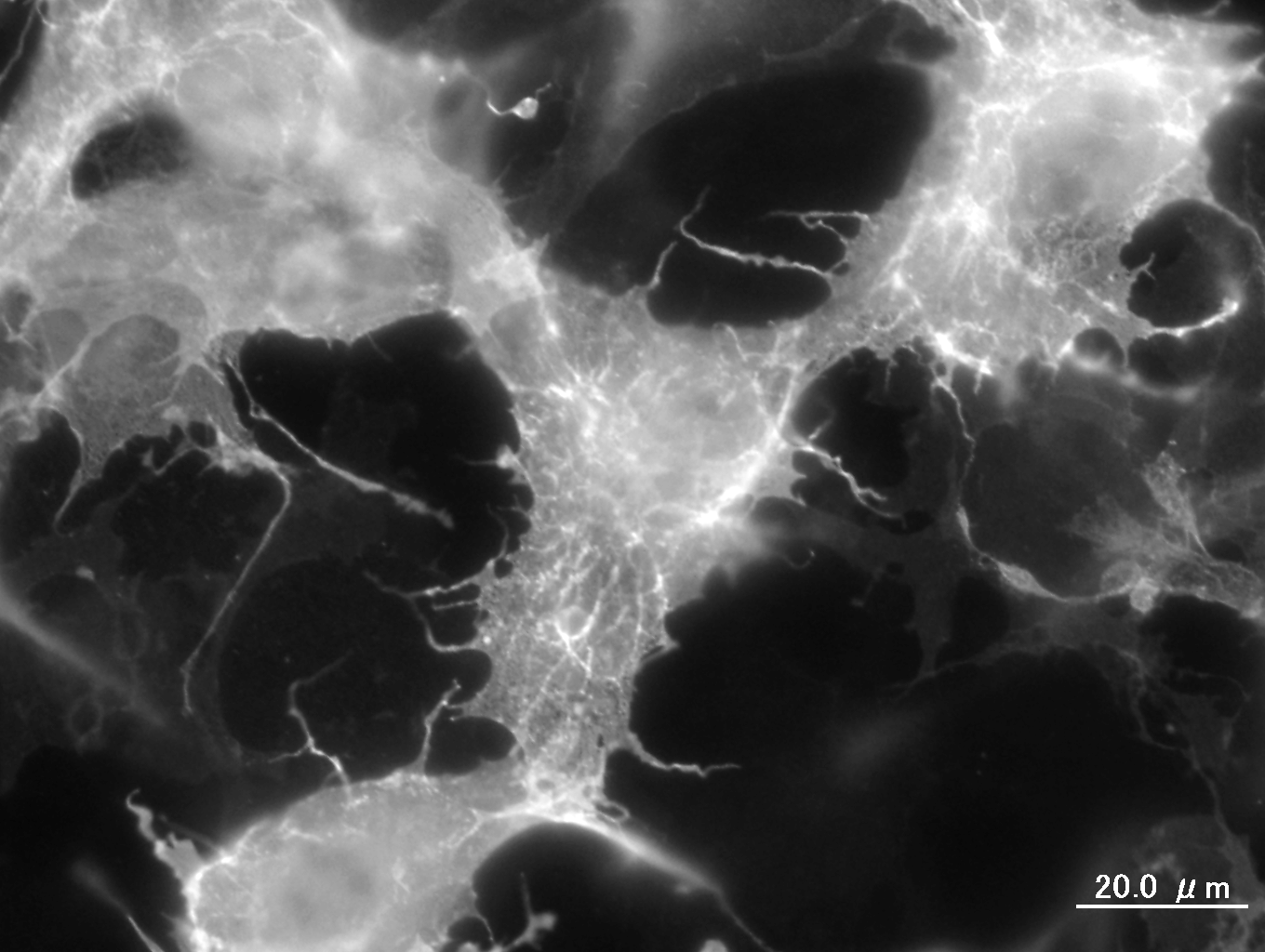

Supplement: Supplementary file 16 — Figure EV and Appendix Source Data [file 44318_2024_268_MOESM16_ESM.zip › Extended View and Appendix Source Data/Figure EV3/FCHO2wt.tif]

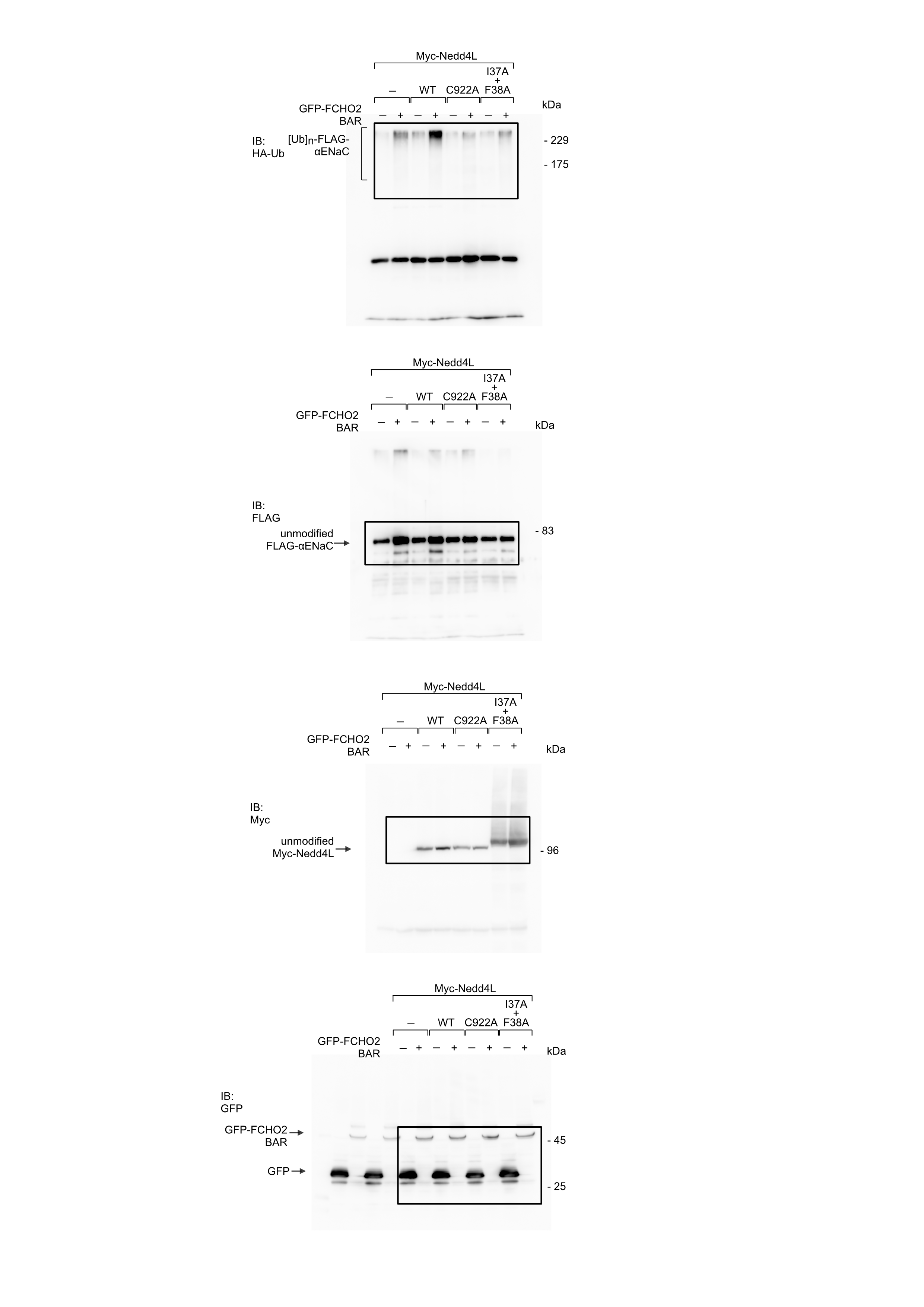

Supplement: Supplementary file 16 — Figure EV and Appendix Source Data [file 44318_2024_268_MOESM16_ESM.zip › Extended View and Appendix Source Data/Figure EV4/FigEV4.tiff]

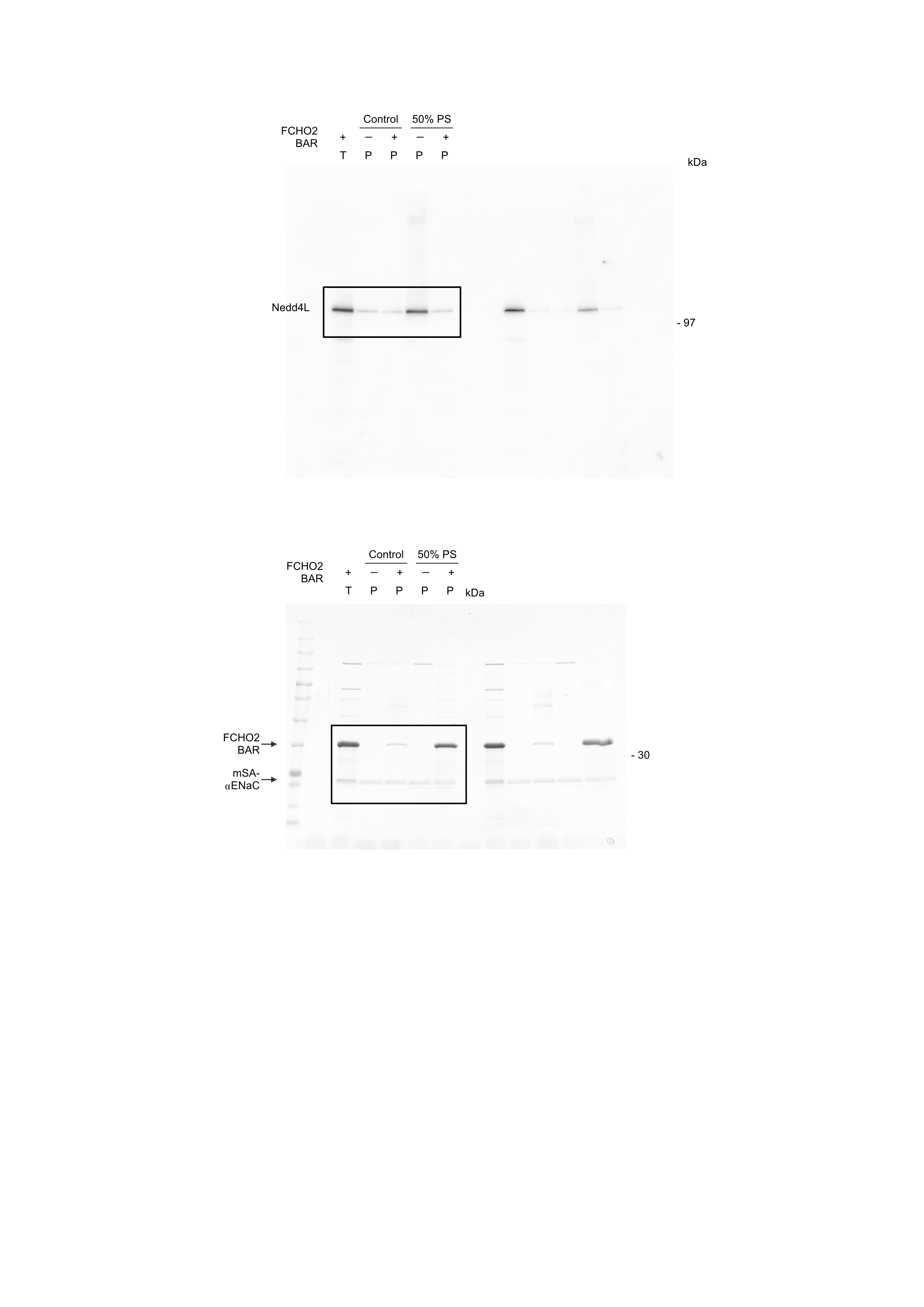

Supplement: Supplementary file 16 — Figure EV and Appendix Source Data [file 44318_2024_268_MOESM16_ESM.zip › Extended View and Appendix Source Data/Figure EV5/FigEV5.tiff]

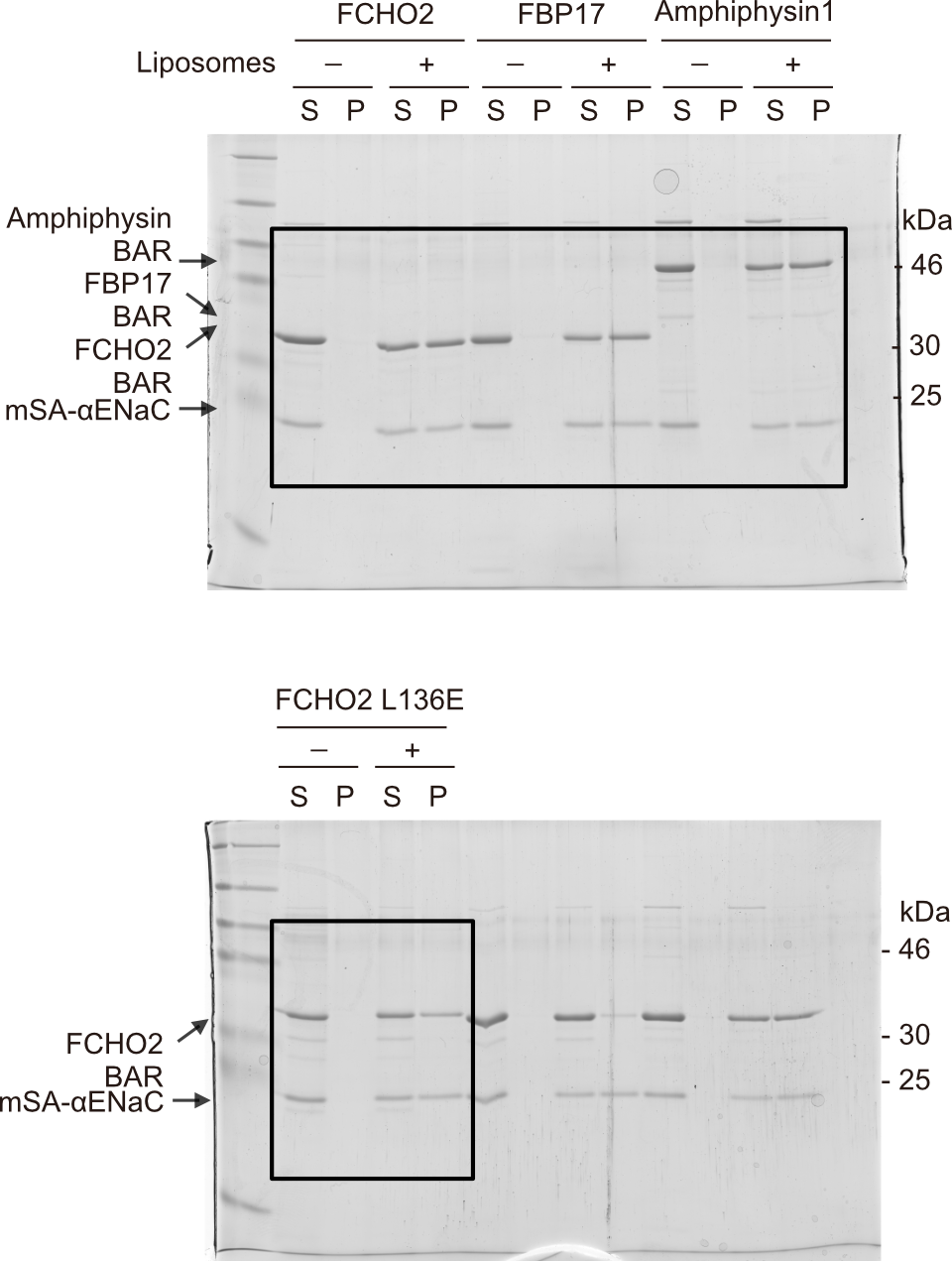

Supplement: Supplementary file 16 — Figure EV and Appendix Source Data [file 44318_2024_268_MOESM16_ESM.zip › Extended View and Appendix Source Data/Figure EV6/EV6A/FigEV6A.tiff]

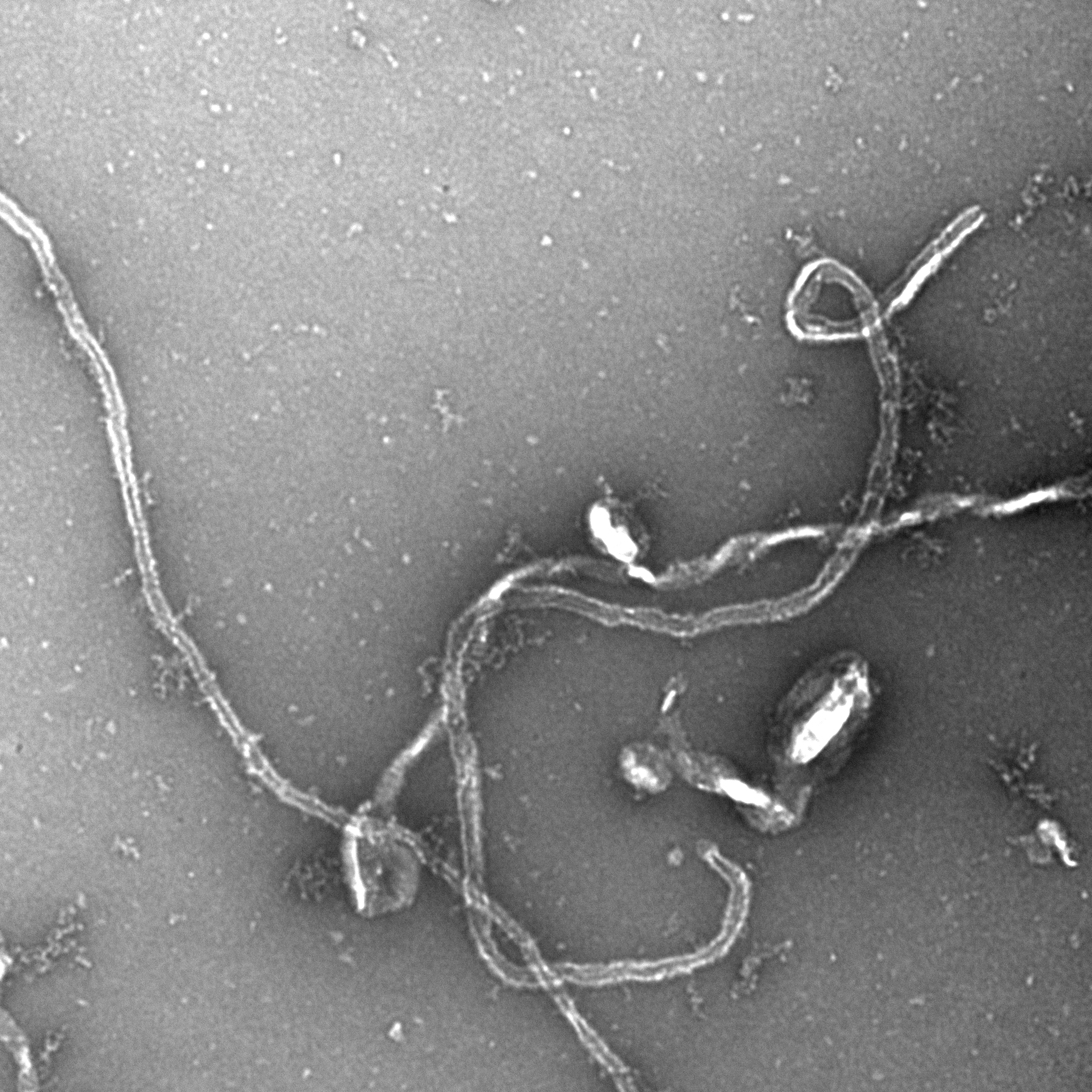

Supplement: Supplementary file 16 — Figure EV and Appendix Source Data [file 44318_2024_268_MOESM16_ESM.zip › Extended View and Appendix Source Data/Figure EV6/EV6B/Amphiphysin1.tif]

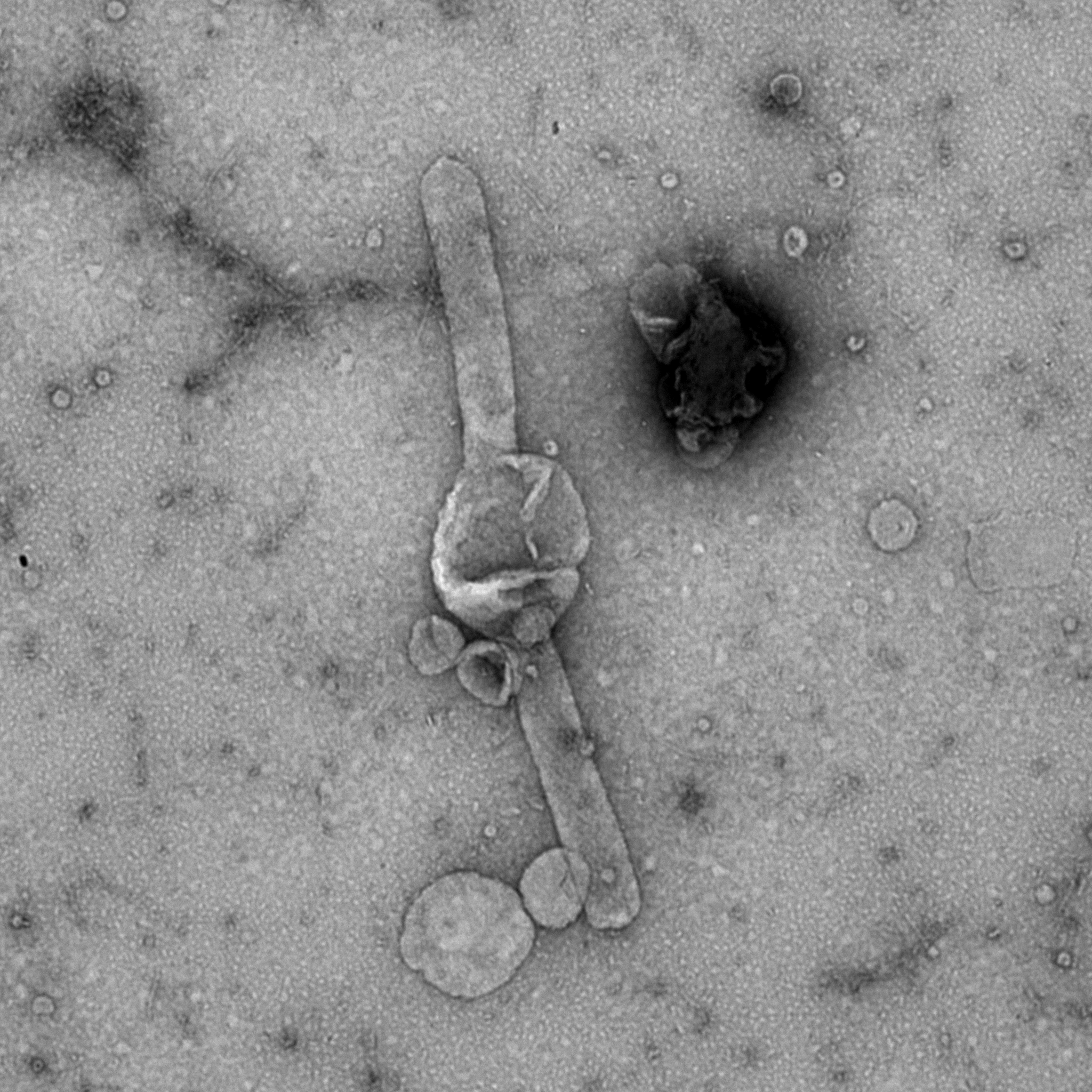

Supplement: Supplementary file 16 — Figure EV and Appendix Source Data [file 44318_2024_268_MOESM16_ESM.zip › Extended View and Appendix Source Data/Figure EV6/EV6B/FBP17.tif]

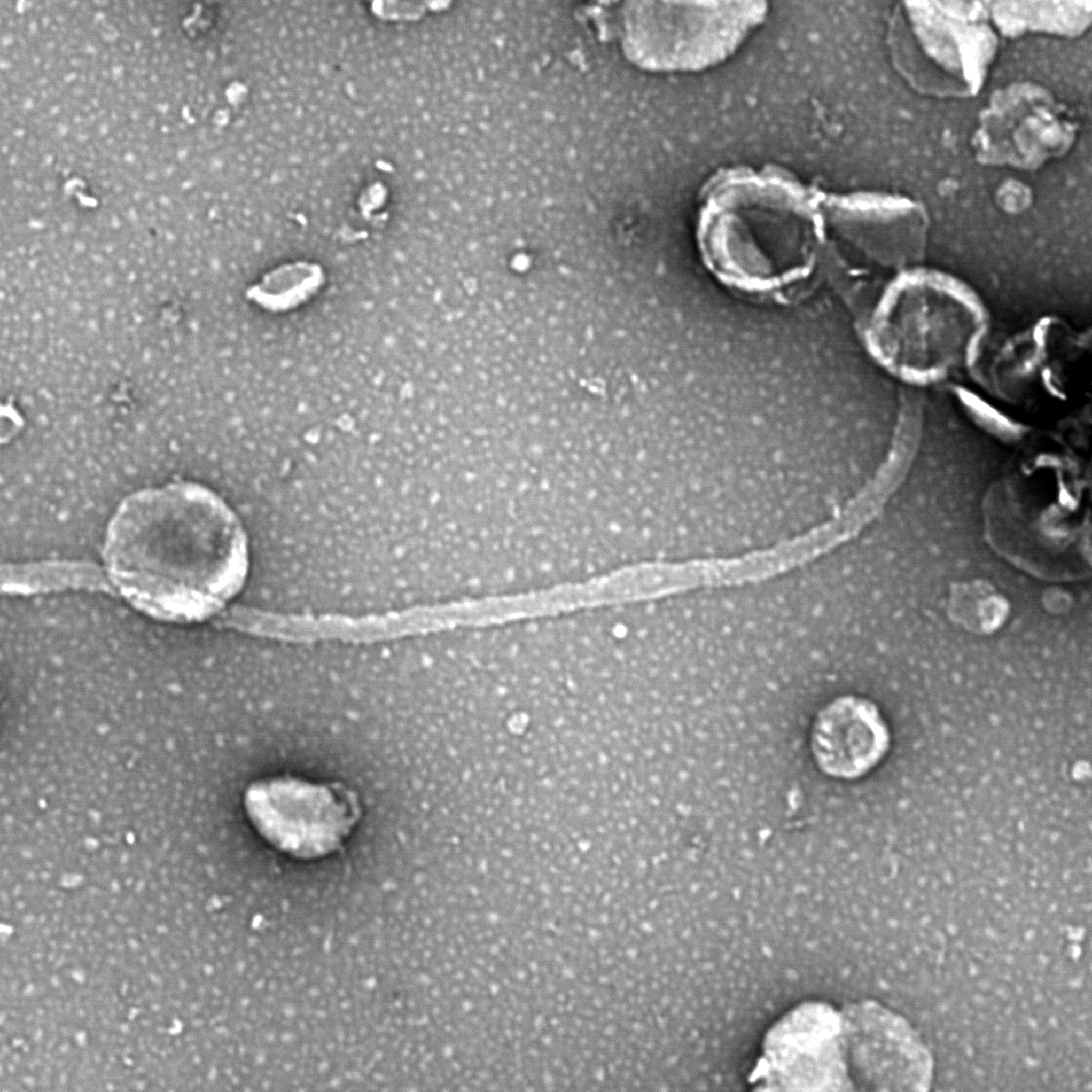

Supplement: Supplementary file 16 — Figure EV and Appendix Source Data [file 44318_2024_268_MOESM16_ESM.zip › Extended View and Appendix Source Data/Figure EV6/EV6B/FCHO2.tif]
