# Supplementary material for: The Nedd4L ubiquitin ligase is activated by FCHO2-generated membrane curvature
Source: EMBO J. 2024 Oct 14;43(23):8. doi: 10.1038/s44318-024-00268-1 (PMC11612235; doi:10.1038/s44318-024-00268-1)
Supplement: Supplementary file 17 — Expanded View Figures [file 44318_2024_268_MOESM17_ESM.pdf]

Expanded View Figures

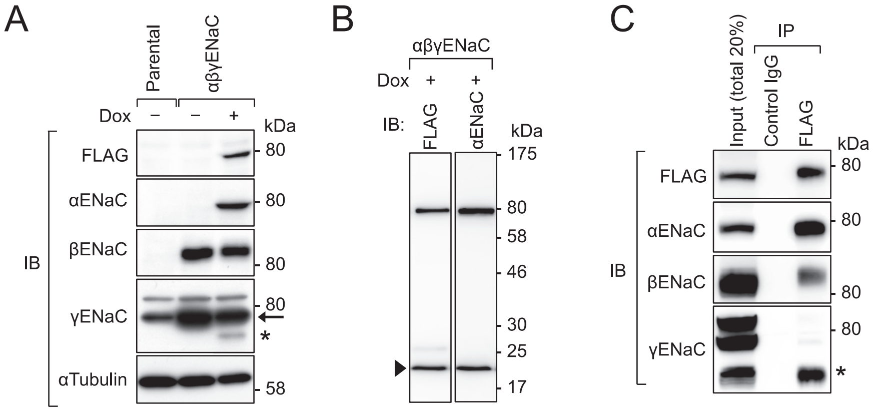

Figure EV1. Expression of ENaC subunits in αβγENaC-HeLa cells.

(**A**, **B**) Doxycycline (Dox)-induced expression of αENaC. αβγENaC-HeLa cells were cultured overnight in the presence or absence of Dox. The lysates of parental HeLa and αβγENaC-HeLa cells were subjected to IB. (**A**) 7.5% gel. (**B**) Gradient gel (5-20%). Anti-γENaC antibody cross-reacted with two bands of endogenous proteins in parental HeLa cells, the lower band of which overlapped with γENaC (arrow) in αβγENaC-HeLa cells. Upon Dox-induced αENaC expression, an additional 70-kDa γENaC band (asterisk) was detected. It has been shown that co-expression of all three subunits induces ENaC maturation, including proteolytic cleavage of α- and γENaC (Hughey et al, 2003). The 70-kDa γENaC band is likely a cleavage product comprising the C-terminal region. The 20-kDa αENaC band (arrowhead) is likely a cleavage product comprising the N-terminal region. (**C**) Association of α-, β-, and γENaC. When αENaC was immunoprecipitated with anti-FLAG antibody from αβγENaC-HeLa cells treated with Dox, β- and γENaC were co-precipitated. Asterisk, 70-kDa γENaC band. Source data are available online for this figure.

 © The Author(s)

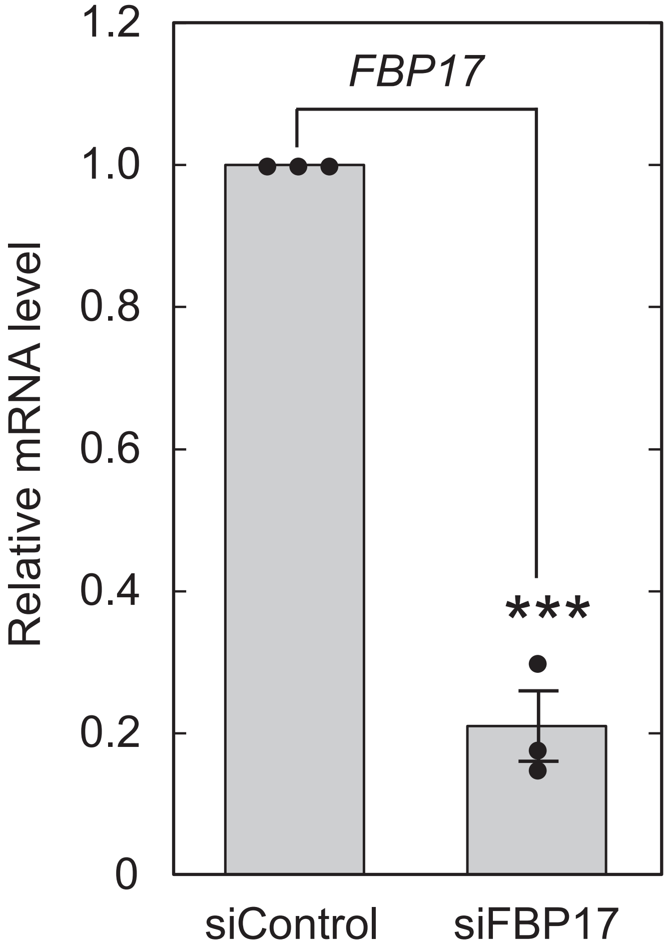

Figure EV2. Knockdown of FBP17 by siRNA.

αβγENaC-HeLa cells were treated with each siRNA, and FBP17 mRNA levels were quantified using real-time PCR. The expression of FBP17 was normalized to GAPDH mRNA levels. Data are shown as the mean ± SEM of three independent experiments. ***$P < 0.001$ (Student's t test). Source data are available online for this figure.

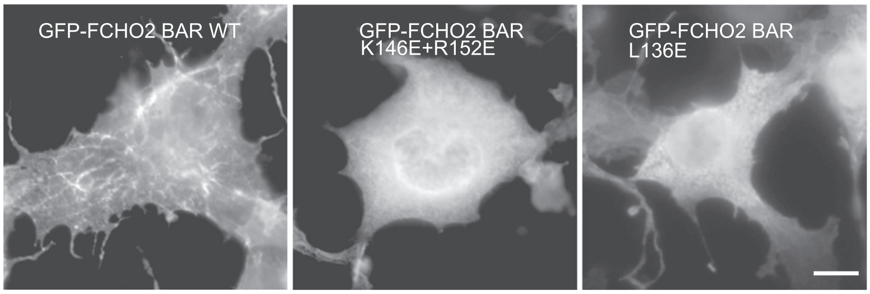

Figure EV3. Inability of FCHO2 mutants to generate membrane tubules.

GFP-FCHO2 BAR domain [wild type (WT) or mutant] was expressed in COS7 cells. Cells were then subjected to immunofluorescence microscopy. Scale bar, 10 µm. Source data are available online for this figure.

 © The Author(s)

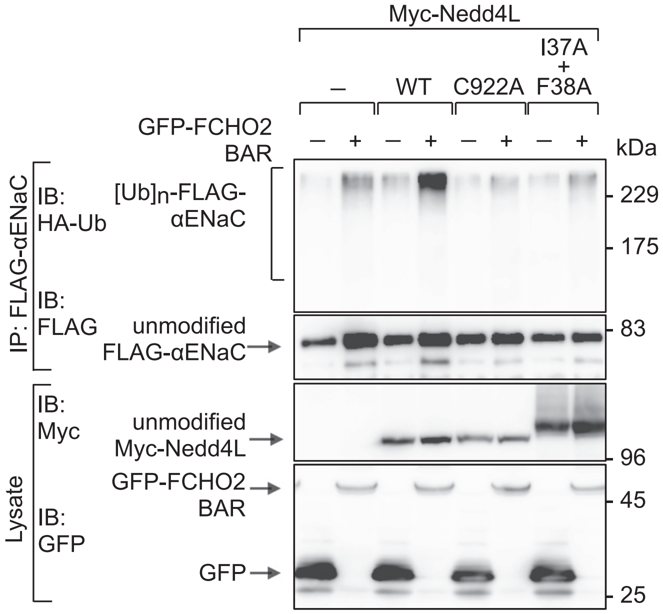

Figure EV4. Inability of Nedd4L mutants to ubiquitinate αENaC.

An in vivo ubiquitination assay was performed with various Nedd4L constructs (each 0.5 μg) using FLAG-αENaC as a substrate in the presence or absence of GFP-FCHO2 BAR domain. Cell lysates were subjected to IP. Samples were analyzed by IB. Source data are available online for this figure.

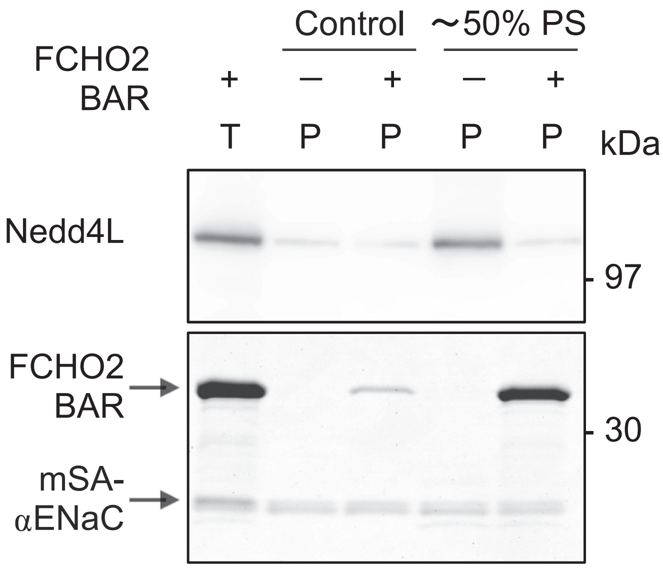

Figure EV5. Inhibition of the liposome binding of Nedd4L by the FCHO2 BAR domain.

A co-sedimentation assay was performed at 0.7 μM Ca^{2+} with control liposomes (0% PS) or brain-lipid liposomes (~50% PS) in the presence or absence of the FCHO2 BAR domain. The total sample (T) and pellets (P) were subjected to SDS-PAGE, followed by IB (upper panel) and CBB staining (lower panel). Source data are available online for this figure.

 © The Author(s)

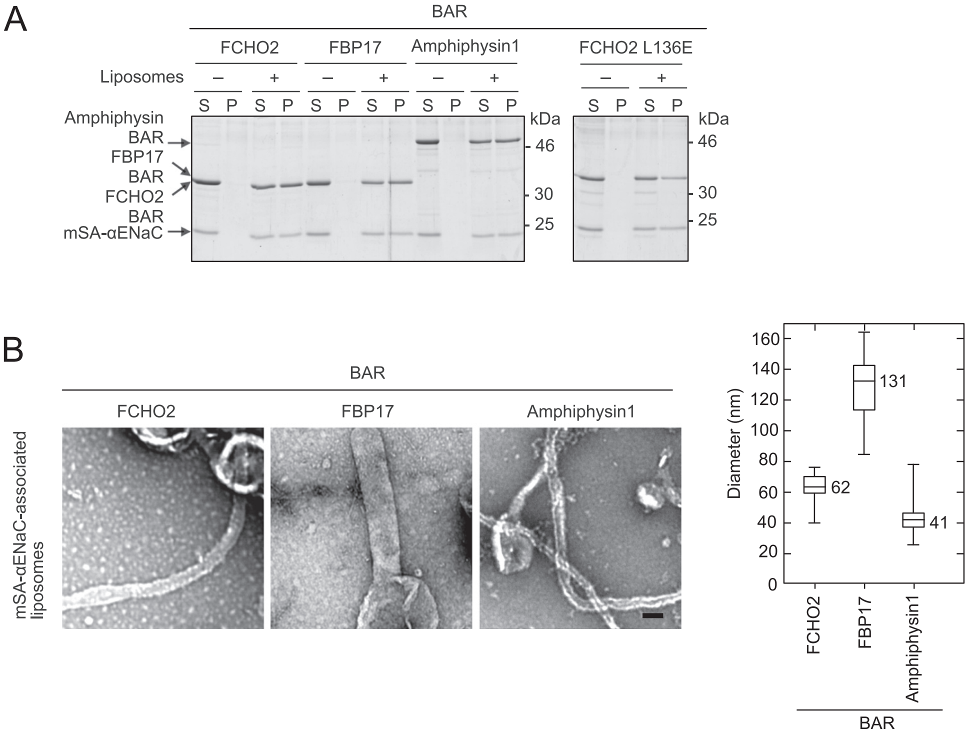

Figure EV6. Membrane binding and curvature generation of BAR domains and an FCHO2 mutant.

Co-sedimentation (**A**) and in vitro tubulation (**B**) assays were performed at 0.7 μM Ca^{2+} with the indicated BAR domains using brain-lipid liposomes (20% PS) that were associated with mSA-αENaC. (**A**) Membrane binding. The supernatants (S) and pellets (P) were subjected to SDS-PAGE followed by CBB staining. (**B**) Curvature generation. Left panel, electron microscopic image. Scale bar, 100 nm. Right panel, distribution of tubule diameters shown by boxplots (number of observations per protein = 22–26). The center line inside the box corresponds to the median, the bounds of the box encompass data points between the first and third quartiles, and the whiskers extend to the minimum and maximum values including outliers. Source data are available online for this figure.
